# Multilevel Residual Complexity Analysis Reveals Origin of Nanomolar Antiviral Bioactives of “Isoquercitrin”

**DOI:** 10.1021/acs.jnatprod.6c00077

**Published:** 2026-04-10

**Authors:** Daniela Rebollar-Ramos, Annie V. Roy, J. Brent Friesen, Guy Harris, Shao-Nong Chen, Michael Chan, Shihua He, Guodong Liu, Wenjun Zhu, Logan Banadyga, James B. McAlpine, Michel Chrétien, Majambu Mbikay, Guido F. Pauli

**Affiliations:** 1 Pharmacognosy Institute and Department of Pharmaceutical Sciences, 14681 University of Illinois Chicago, Chicago, Illinois 60612, United States; 2 Functional Endoproteolysis Laboratory, Montreal Clinical Research Institute, Montreal, Quebec H2W1R7, Canada; 3 Physical Sciences Department, 6903 Dominican University, River Forest, Illinois 60305, United States; 4 Special Pathogens Program, National Microbiology Laboratory, Public Health Agency of Canada, Winnipeg ,Manitoba R3E3R2, Canada; 5 Department of Medical Microbiology and Infectious Diseases, University of Manitoba, Winnipeg, Manitoba R3E0J9, Canada

## Abstract

The remarkable reported *in vitro* and *in vivo* antiviral activity of a commercial, naturally derived, isoquercitrin sample (IQC90) against Ebola (EBOV), Zika virus (ZIKV), and SARS-CoV-2 could not be confirmed with a greater purity isoquercitrin (IQC). To resolve this discrepancy, IQC90 was subjected to a two-step, quantitative bioassay-guided fractionation employing countercurrent separation and gel filtration monitored by inhibition of syncytium formation in HEK293 cells transfected with SARS-CoV-2 spike protein and ACE2. This process revealed the IQC90 antiviral activity to be due to a new family of 21-hydroxyoleanane-3-*O*-oligosaccharides, named dicitriosides, present at <1 mol %, rather than IQC. The two dominant dicitriosides, the hexoside, dicitrioside A_1_ (**1**), and the pentoside, dicitrioside B_1_ (**2**), inhibited syncytia formation with an IC_50_ = 0.530 μM; 25-fold more active than IQC90 (IC_50_ = 12.8 μM). Beyond anti-SARS-CoV-2 activity, dicitrioside B_1_ (**2**) also prevented EBOV infection of Vero E6 cells, supporting the conclusion that the dicitriosides inherit the promising potential of IQC90 as antiviral leads for clinical translation. Ultrahigh field 1.1 GHz NMR spectroscopy, particularly 1D selective TOCSY experiments and nuclear genotyping via quantum-mechanical spin analysis, enabled structure elucidation and provided definitive reference points for the dicitriosides as complex oligoglycoside esters.

## Graphical abstract

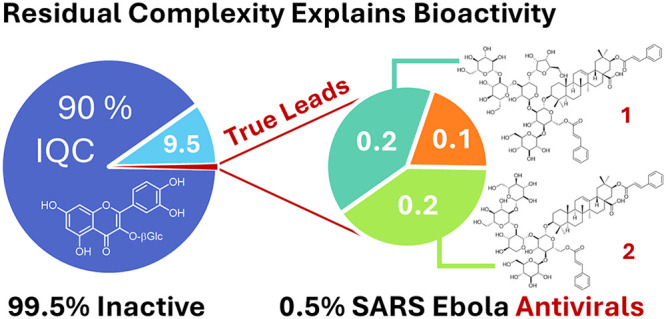

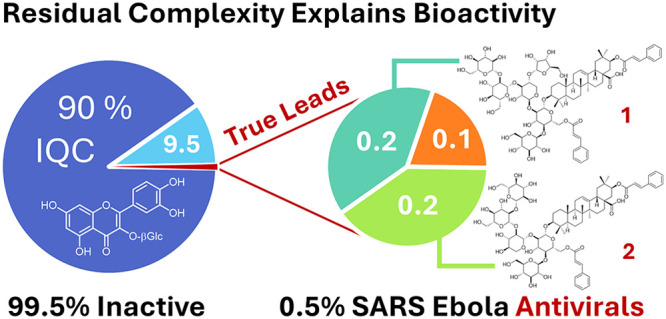

## Introduction

The flavonol glycoside, isoquercitrin (IQC; quercetin-3-*O*-β-d-glucoside; sometimes referred to as isoquercetin; Figure 1)^1^ along with a wide range of congeners occur extensively in vascular plants. Earlier reports proposed that IQC had promising antiviral activities against influenza A and B^2^ and Dengue virus (DENV2/DENV3);^3^ this was later extended to EBOV^4,5^ and ZIKA virus.^6,7^ A remarkable finding from our initial assays was the 100% survival rate of mice infected with lethal doses of EBOV and treated with a commercial, naturally derived, “isoquercitrin” material containing at least 90% IQC (IQC90).^4^ Encouraged by these results, and prompted by urgent healthcare needs, antiviral studies of IQC90 were expanded to SARS-CoV-2, and IQC90 confirmed to be active *in vitro*. However, when later evaluated for efficacy against SARS-CoV-2 infection, “isoquercitrin” activity depended upon batch and supplier (see Purity–Activity Relationship of “Isoquercitrin” Substances).

**Figure 1:**
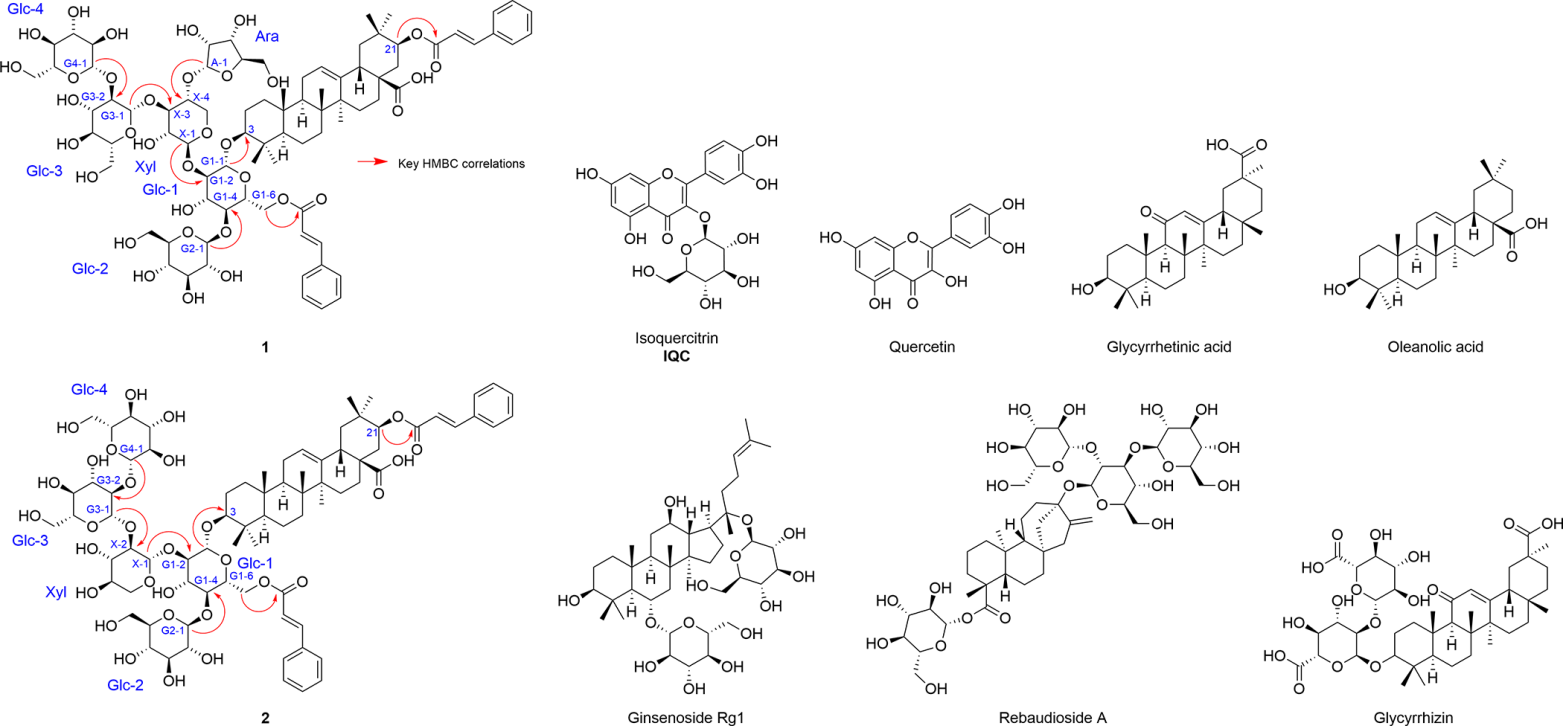
Chemical structures of the compounds studied, including key HMBC correlations in **1** and **2**.

IQC’s near-ubiquitous occurrence, its very many alleged bioactivities,^8,9^ and lack of structural activity relationships (SARs) raised the question of the potential role of Residual Complexity (RC)^9–11^ in both our and reported observations: could even small differences between substances labeled “isoquercitrin” and the chemically uniform IQC explain the observed bioactivities?

Reductionist bioassay-guided research has led to many apparent panaceas: compounds highly abundant in their natural source(s), with hundreds of reported bioactivities and purported utilities as potential drugs, yet consistently failing hit-to-lead translation despite major efforts. Curcumin, resveratrol, β-sitosterol, quercetin, and rutin are such prototypic Invalid/Improbable/Interfering Metabolic Panacea (IMP) candidates.^9^ As IQC combines many of these IMP characteristics, and being a very close analogue of quercetin and rutin, the observed *in vivo* antiviral activity of IQC90 was surprising, challenging the reductionist and/or the panacea paradigms.

Thus, the starting hypothesis for this study was that the true antiviral activity of “isoquercitrin” was hidden in its RC. This was nurtured by two experiments: (a) high-purity IQC (98.5% by relative ^1^H NMR [rel-qHNMR]) was devoid of antiviral activity; (b) rel-qHNMR analysis of the bioactive sample of IQC used for the prior biological experiments, designated as 90% pure by HPLC (“IQC90”), confirmed 89.8% IQC. Unsurprisingly, rel-qHNMR also found ∼9.4% congeneric flavonoid glycosides plus, more significantly, ∼0.80% of aliphatic glycosidic components, which escaped reliable quantification due to the lack of molecular weight information.

Combining these results with the above considerations about process, source, and reductionism paradigms led to the working hypothesis that IQC90, prototypic for IQC and other IMPs, contains very minor residual constituents that are, in this case, highly potent antiviral agents and are not flavonoid congeners. Bioassay-guided fractionation of IQC90 revealed that multiple layers of RC had to be overcome to uncover the identity of the true SARS-CoV-2 and EBOV antiviral active principles.

Herein, we report the application of a rigorous, quantitative bioassay-guided fractionation approach to address the RC of IQC90. This resulted in the isolation and structure elucidation of a new family of triterpenoid oligoglycosides present in the sample at sub 1 mol % that fully explains the apparent “isoquercitrin” phenotype.

## Results and Discussion

### Purity–Activity Relationship of “Isoquercitrin” Substances

The known pathogenesis of SARS-CoV-2 involves interaction of the spike glycoprotein on the viral surface with the angiotensin-converting enzyme 2 (ACE2) receptor, leading to syncytium formation, entry of the virus, and RNA release in the host cell. Syncytialization marks the polynucleated stage of the host cell infection process of several enveloped viruses^12^ and was evaluated during the present study. In recent studies, a sample containing the flavonol aglycone, quercetin (Figure 1), inhibited the syncytium formation in an assay mimicking the starting step of SARS-CoV-2 infection by cotransfecting the viral spike protein (S protein) and the human ACE2, its receptor, into human embryonic kidney 293 (HEK293) cells (HEK293 Syncytialization Assay; HEKSA).^13^ However, the limited oral bioavailability of quercetin inspired the use of its glucoside, IQC, as a potential prodrug.

Testing IQC from Supplier A (IQC-SA) for its ability to block syncytialization in HEKSA (Figure 2A) revealed an IC_50_ of 12.8 μM (Figure 2B). Additionally, IQC-SA inhibited SARS-CoV-2 infection in Vero E6 cells, reducing both the quantity of infectious virus and viral RNA produced. IQC-SA demonstrated an IC_50_ of 9.6 μM via the Median Tissue Culture Infectious Dose (TCID_50_) assay (Figure 2C) and 7.3 μM by quantitative reverse transcription polymerase chain reaction (RT-qPCR; Figure 2D). With a half cytotoxic concentration (CC_50_) of 71.4 μM in Vero E6 cells (Figure 2E), IQC-SA showed a satisfactory selectivity index (SI = CC_50_/IC_50_) of 7 to 10, indicating its potential therapeutic value as an anti-COVID-19 drug candidate. Separately, to confirm the suitability of this assay, we evaluated the inhibitory efficacy of the broad-spectrum antiviral drug galidesivir (also known as BCX4430) against SARS-CoV-2.^14^ The drug reduced SARS-CoV-2 replication, although less effectively than treatment with IQC-SA (Figure S1A). No cytotoxicity was observed (Figure S1C).

**Figure 2:**
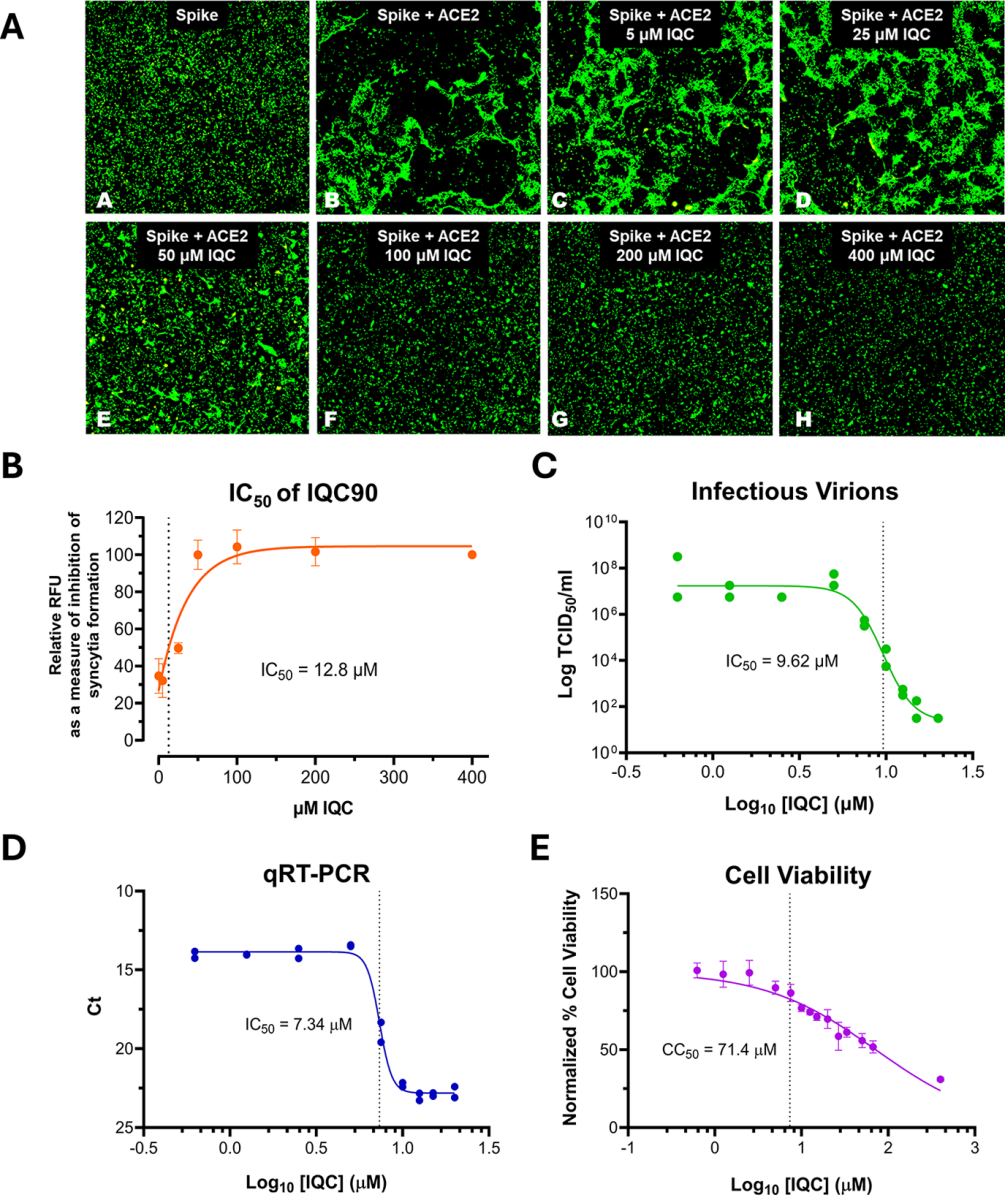
Preliminary biological activity of IQC-SA. (A) HEK293 cells were transfected with an expression vector for SARS-CoV-2 Spike protein linked to GFP AND an empty vector (panel A) OR an expression for hACE2 (panels B–H). After a 4 h incubation, the transfection medium was replaced with fresh media without (panels A and B) or with IQC at different concentrations (panels C–H). Incubation was resumed for 16 h, and cell morphology and fluorescence were captured. (B) Half inhibitory concentration (IC_50_) of IQC in HEKSA (biological triplicates; mean ± SD; linear regression 95% CI= 5.7 to 29.4 μM). (C) Determination of the concentration that provides half the maximal infection in infected Vero E6 cells with SARS-CoV-2 (technical duplicates). (D) Determination of the amount of SARS-CoV-2 RNA by RT-qPCR after 2 days postinfection of Vero E6 cells with SARS-CoV-2 (technical duplicates). (E) Uninfected Vero E6 cells were treated with varying concentrations of IQC for 48 h, and cell viability was assessed using the CyQUANT XTT assay kit (biological quadruplicates; mean ± SD; linear regression 95% CI= 59.5 to 87.9 μM).

These promising *in vitro* results motivated *in vivo* experiments with Syrian hamsters,^15^ a common model for evaluating the therapeutic efficacy of potential anti-COVID-19 drugs. However, in this case, the IQC was obtained from Supplier B (IQC-SB). The treatment of SARS-CoV-2 infected hamsters with this IQC-SB completely failed to ameliorate their clinical profile relative to untreated counterparts: survival, weight loss, and clinical signs, as well as swab and tissue viral loads per gender were all statistically similar between treated and untreated groups (Figure S2).

According to the material data sheets, the HPLC-UV purities of IQC-SA vs IQC-SB were ≥ 90% and ≥ 99%, respectively. Biological comparison of IQCs from additional suppliers using HEKSA showed that only the 90% pure IQC-SA (hereafter labeled IQC90) inhibited syncytialization, while all other IQC samples of ≥ 96% purity were inactive. Table 1 summarizes these findings as well as the rel-qHNMR assessment of the IQC samples from the different suppliers; images of the syncitialization and ^1^H NMR spectra are shown in Figures S3–S9.

**Table 1:** Summary of Key Characteristics of the IQC Materials from Different Suppliers: Labeled Purity, rel-qHNMR Purity, and HEKSA Bioactivity

IQC suppliers	labeled purity [%]	rel-qHNMR purity	HEKSA bioactivity
IQC-SA-90	≥90	89.8	active
IQC-SA-98	≥98	>99	inactive
IQC-SB	99.6	98.8	inactive
IQC-SB-2^a^	99.6	98.1	inactive
IQC-SC	99	96.1	inactive
IQC-SD	98	98.2	inactive
IQC-SE	99	NA^b^	inactive
IQC-SF	97.7	97.1	inactive
IQC-SG	99.7	98.8	inactive

a Identified as quercetin 3-*O*-β-d-glucofuranoside.

b Not available due to depletion of supply by bioassay.

The ability of IQC from different sources to inhibit infectious SARS-CoV-2 was also assessed (Figure S10) by treating Vero E6 or Caco-2 cells with increasing concentrations of IQC from suppliers A-C, followed by infection with SARS-CoV-2 at an MOI of 0.1. At day 2 postinfection, only IQC90 showed a decrease in virus RNA levels, with IC_50_ values ranging from 7.6 to 12.6 μM. Neither IQC-SB nor IQC-SC showed any inhibitory effect in either cell line, consistent with the lack of efficacy of IQC-SB in the SARS-CoV-2 hamster model (Figure S2). Additional experiments compared the ability of IQC-SA/B/C to inhibit EBOV infection (Figure S11). Vero E6 cells were treated with increasing concentrations of drug and then infected with a recombinant EBOV expressing EGFP (EBOV-EGFP) at an MOI of 0.1. At day 4 postinfection, IQC-SA (IQC90) was once again the only substance that showed a decrease in EGFP fluorescence, indicating a decrease in virus infection, with IC_50_ values of 6.2 and 5.4 μM.

Initial chemical evaluation utilized ^1^H qNMR analysis to compare IQC90 with an IQC substance of 98% purity (IQC98; Figure S12). NMR detection revealed the presence of very minor nonflavonoid compounds in IQC90, suggesting that the Residual Complexity (RC) was indeed responsible for the antiviral activity. This set of results convinced us to initiate the purification and identification of the minor active ingredient of IQC90 with HEKSA as bioactivity monitor. LCMS analysis of these two samples (Figures S13 and S14) revealed additional minor components such as the aglycone quercetin and a diglycoside, tentatively assigned as rutin present in IQC90. However, LCMS analysis did not display another obvious nonflavonoid impurity, as indicated by NMR. In this case, NMR spectroscopy demonstrated to be a powerful universal detector for the identification of additional impurities via the structural information provided.

### Our Approach to Identifying the Minor Bioactive Component

The “gold-standard” experimental approach to identify which, if any, minor constituents or impurities are responsible for observed antiviral activity is a process called bioassay-guided fractionation. Briefly, the bioactive sample or extract is chromatographically separated and the fractions assessed for biological activity. This process is iterated, typically three or more times, until the bioactivity is associated with a defined component of the original sample. This requires a biological assay that is robust and quantitative, as well as functional assays to demonstrate translational bioactivity. The choice of fractionation methods remains, in large part, an “art”. Rigorous quantification of bioactivity recovery throughout the fractionation process was essential to avoid prematurely or falsely assigning activity to an invalid component.

#### Step 1: CCS Fractionation of IQC90—Knocking Down IQC

The nature of the sample used for the hunt of the real bioactive components led to the use of Counter Current Separation (CCS), a liquid–liquid technique for the separation of complex mixtures. The main advantage of CCS over other chromatographic methods includes the ability to knock out one compound from large quantities of a complex mixture:^16^ in this case, CCS produced the knockout of IQC (as 90% abundant) from 3 g of IQC90. The resulting fractions were labeled as A, B1, B2­(a-c), B3, B4, and C (Figure 3A). The main component, IQC, was isolated in the bulk fraction (B2), whereas all the RC was present in the remaining fractions.

**Figure 3:**
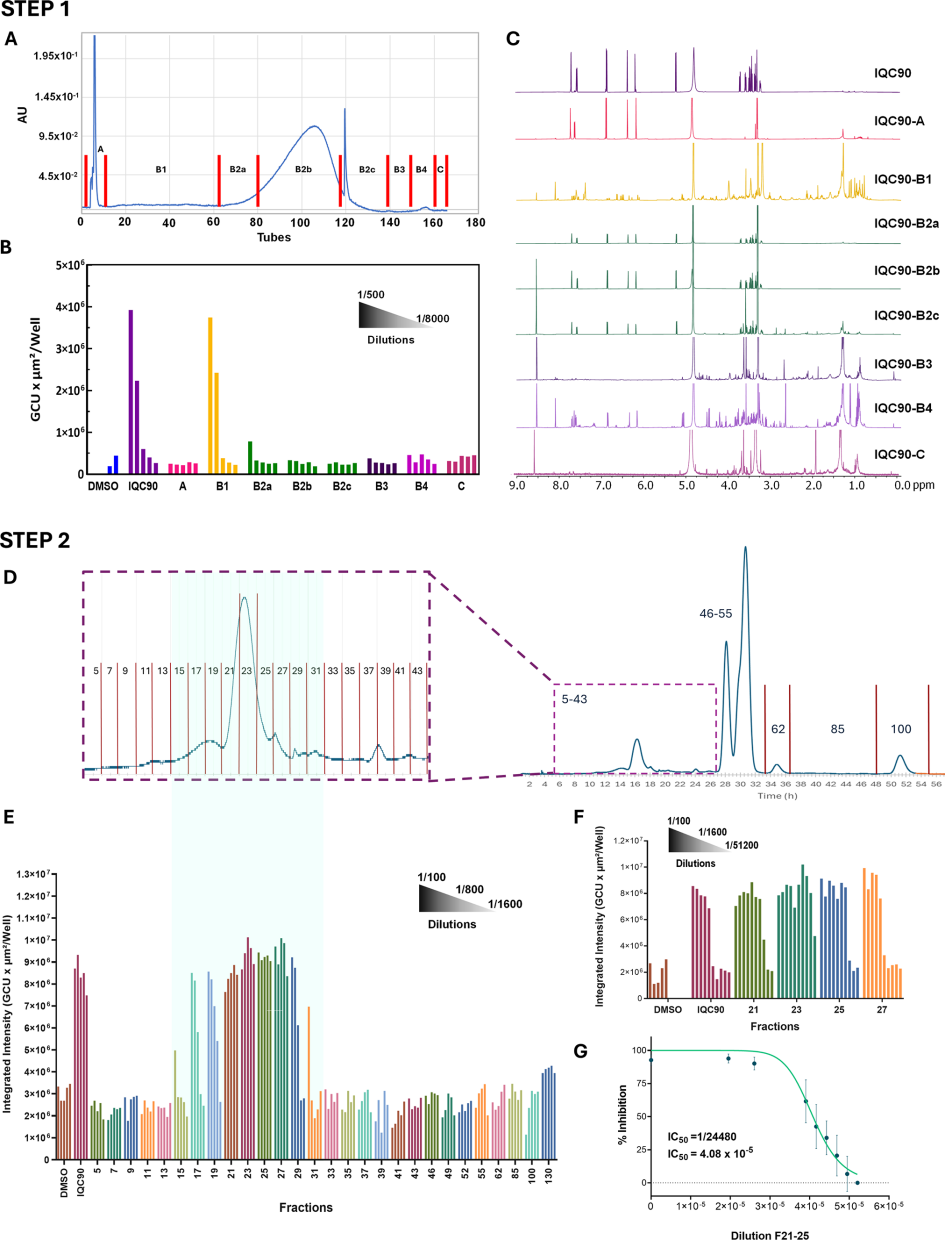
Two-step bioassay-guided fractionation of IQC90. Step 1: Primary CCS fractionation of IQC90: (A) chromatogram at UV 254 nm of the CCS fractionation of IQC90. (B) HEKSA fluorescence readout (biological duplicates) showing only fraction IQC90-B1 active even at 1/1000 dilution; IQC90 and CCS recovery sample served as references, DMSO as vehicle. (C) ^1^H NMR monitoring of the fractions obtained from the first step of separation. Fraction IQC90-A contained the aglycone, quercetin, IQC90-B1 the bioactive terpenoids. IQC90-B2 was high-purity IQC mainly in IQC90-B2a/b, IQC90-B3 unidentified minor components, IQC90-B4 a diversity of flavonoid glycosides. Step 2: Secondary Sephadex LH-20 fractionation of IQC90-B1. (D) Chromatogram at UV 260 nm of the Sephadex LH-20 (7-m) fractionation of the active fraction IQC90-B1. Expansion of the chromatographical zone of interest. (E) Antiviral monitoring of the fractions (1/100 to 1/1,600 dilutions) in HEKSA (biological duplicates), showing the bioactives in fractions F15–F31 (highlighted). The fractions containing IQC, quercetin, and other flavonoids were devoid of any activity. (F) Higher dilutions (1/3200–1/51,200) of the most active fractions (F21–F27) pinpointed F23 as the most active, even at 1/51,200 dilution (singular replicate). (G) Half inhibitory concentration (IC_50_) of F21–25 in HEKSA (biological triplicate; mean ± SD; linear regression 95% CI = 3.9 × 10^–5^ to 4.2 × 10^–5^).

Figure 3C shows the ^1^H NMR analyses of all the fractions, performed to determine the identity of the components in the fractions. The observance of its characteristic spin systems allowed the identification of IQC in fraction B2. For fraction A, the absence of sugar components and the characteristic peak patterns for quercetin were observed, meaning that the aglycone of IQC eluted in the front of the separation. Fractions B3 and C displayed peak patterns attributed to saccharides and fatty acid residues. In contrast, fraction B4 showed typical peak patterns from other glycosylated flavonoids (Figure 3C). Importantly, Fraction B1 presented the characteristic peak patterns for likely glycosidic (tri)­terpenoid compounds, with several likely methyl singlets in the low-frequency region (0.5 to 1.2 ppm); however, some aromatic resonances and saccharide residues were also observed (Figure 3C).

Here, we want to introduce the concept of *aliquot-based* bioassay-guided fractionation, which uses the original material as a reference for comparison throughout the fractionation steps. In this approach, all samples are labeled as mg equivalents (mgE) to the original source. Thus, the amount of compound in each fractionation step is not needed: all calculations are based on the aliquots from the original sample, rather than the concentration of each sample itself. The advantage of this method is that it enables tracking of the bioactive components, without any loss of bioactivity through the fractionation steps, which leads to an accurate comparison of potency among the fractions, as everything is expressed based on the original material.

The samples tested in HEKSA were *aliquot-based* on the amount of IQC90 as the original material, i.e., as 1-mg equivalents of IQC90 (“1 mgE IQC90”). The bioassay showed that all the activity was present in fraction B1 (Figure 3B), with activity observed in dilutions 1/500 and 1/1,000, showing that the activity was present at 1 μgE of IQC90. Fractions A (quercetin), B2 (IQC), and B3–B4–C remained as inactive fractions.

#### Step 2: Fractionation of B1—Finding the Active Components

Further fractionation of B1 to identify the active components in IQC90 was pursued. CCS was used for the first step of the fractionation to remove IQC from the other components of this complex mixture. However, as the active compound remained unknown, the selection of an orthogonal method was proposed; in this case, a Sephadex LH-20 column. Sephadex LH-20 is not based on the liquid–liquid K value of the components, but on the molecular weight discrimination and affinity for the gel, allowing the separation of flavonoids, flavonoid-glycosides, and terpene-like components.^17^

The fractionation of 60 mg of fraction B1 was performed with a 7-m Sephadex column system in MeOH solvent, yielding 100 fractions (Figure 3D). The antiviral activity (HEKSA) was tested for all fractions, by pooling two fractions, hence, fraction 31 (F31) comprised tubes 30 (T30) and 31 (T31). According to the results in the first step, the activity was present until 1 μgE IQC90, so the aliquot prepared for this case was 150 mgE IQC90. The bioactivity was only observed from F15 to F31 (Figure 3E).

Due to the increased potency observed for this set of fractions (still active after 1/1,600 dilution), further dilutions of the most active fractions F21 to F27 were tested (Figure 3F). The bioactivity for all these fractions was maintained up to a 1/6400 dilution, showing the high potency of the components present in those fractions (2.6 μgE IQC90). Fraction F23, displayed bioactivity even at a 1/51,200 dilution (0.3 μgE IQC90).

UHPLC analysis (Figure S15) of the active fractions showed the presence of a family of compounds; however, tube 21 (T21) showed a major component in the UV profile, hence the full fraction was analyzed by NMR to establish the chemical structure of the main compound. Tube 23 (T23) also displayed a main component in the UV profile, with traces of the compound present in T21 and other similar compounds. The further separation of these active components could not be performed due to the low quantities obtained, the yields of the active individual fractions range from 1 to 3 mg.

### Structural Elucidation of Dicitrioside A_1_ (**1**)

Compound **1** was identified as the main component of T21. Rel-qHNMR analysis of the sample showed the purity of the compound as 74%, with the identification of two main impurities: 21% of compound **2** and 5% of an aliphatic residue. This sample had the highest purity among the isolates, hence subject to structural elucidation. HRMS yielded an *m*/*z* value of 1644.7352 corresponding to the molecular formula C_82_H_116_O_34_ with 25 degrees of unsaturation. The analysis of the MS/MS fragmentation patterns identified the aglycone with an *m*/*z* ratio of 601.3917 [M-H]^−^. Analysis of a 600 MHz ^1^H NMR spectrum ascertained three distinct structural components: two cinnamic acid residues, an aliphatic alicyclic aglycone (identified later as the triterpene), and a hexasaccharide with six anomeric protons.

While the peak patterns in the aliphatic and aromatic regions were reasonably dispersed at 600 MHz, the substantial peak overlap in the oligosaccharide region challenged the elucidation process: 32 protons resonated within only 1.0 ppm (3.1 to 4.1 ppm), with three protons overlapping within just 0.05 ppm. Combined with achievable sample and purity limitations, this hampered the further elucidation of coupling patterns essential for stereochemical assignments within the entire molecule. Coincidentally, a new 1.1 GHz 3 mm cryoprobe NMR spectrometer became available at NMRFAM: its first application for the liquid NMR analysis of this “small” molecule essentially doubled dispersion (Figure 4) and yielded a series of ^1^H-detected spectra that enabled unequivocal assignments including all H,H coupling patterns. Collectively, the structure elucidation was achieved via a combination of ^1^H, ^13^C, HSQC, HMBC, COSY, NOESY, and 1D selective TOCSY (SelTOCSY) experiments at both 600 MHz and 1.1 GHz.

**Figure 4:**
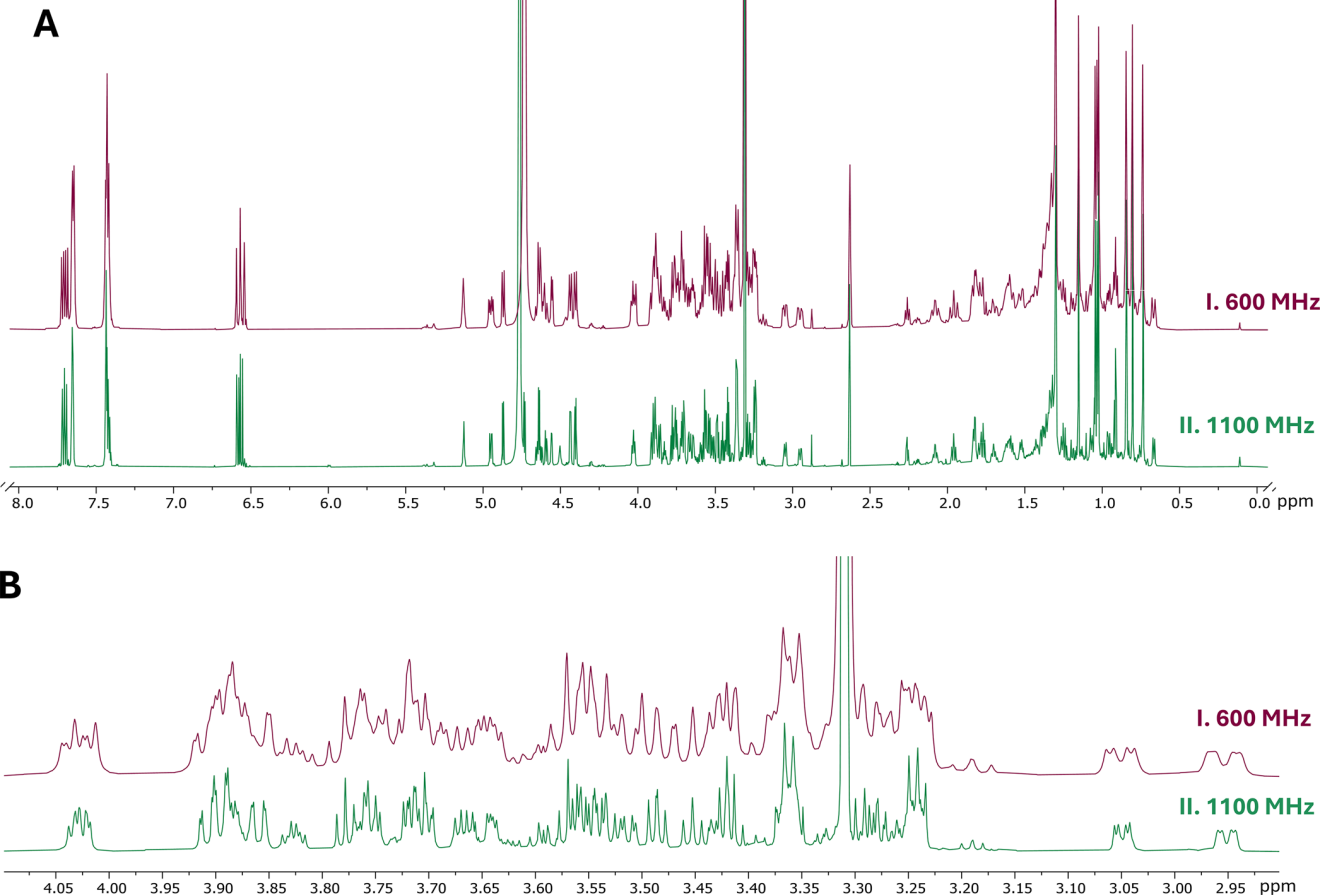
^1^H NMR spectra of fraction F21, showing compound **1** as the main component. Panel (A) shows the overview of spectra acquired at both 600 MHz and 1.1 GHz. Panel (B) shows an expansion of the sugar range between 2.90 and 4.10 ppm and exemplifies how the increased dispersion observed at 1.1 GHz enabled the first nuclear genotyping of a molecule of this size and complexity via quantum mechanics-based ^1^H iterative functionalized Spin Analysis (HifSA). The region amplified comprises the overlapping peak patterns of 34 protons, which were fully explained by the numeric HifSA profiles in Tables 2 and 3.

The aglycone was identified as an oleanolic-like triterpene. The presence of an oxygen-based methine (δ_H_ 4.944 ppm) in position 21 revealed the aglycone as a 21-hydroxy-oleanolic acid derivative. An HMBC correlation between H-21 and the carboxylic carbon at 168.01 ppm indicated the position of one of the two cinnamic acid residues, identifying the aglycone as 21-cinnamoyloxy-oleanolic acid.^18^ This was corroborated by establishing the nuclear genotype, encoding the relative configuration of the acylated triterpenoid aglycone, by HifSA profiling via quantum-mechanical spin analysis.^19^

The structure of the oligosaccharide was assembled primarily via SelTOCSY and HMBC experiments. Exciting the anomeric protons with the selective pulse experiments served as entry points for the entire spin system of each individual sugar and fully resolved peaks overlap with neighboring sugars (Figure 5). This led to the observance of the typical 4-fold *trans*-diaxial couplings (H-1 to H-4) of four β-glucopyranoses and one β-xylopyranose. The more complex, noncyclohexanoid *J* coupling pattern of the sixth sugar was assigned to an α-arabinofuranose residue, in line with the literature.^20^

**Figure 5:**
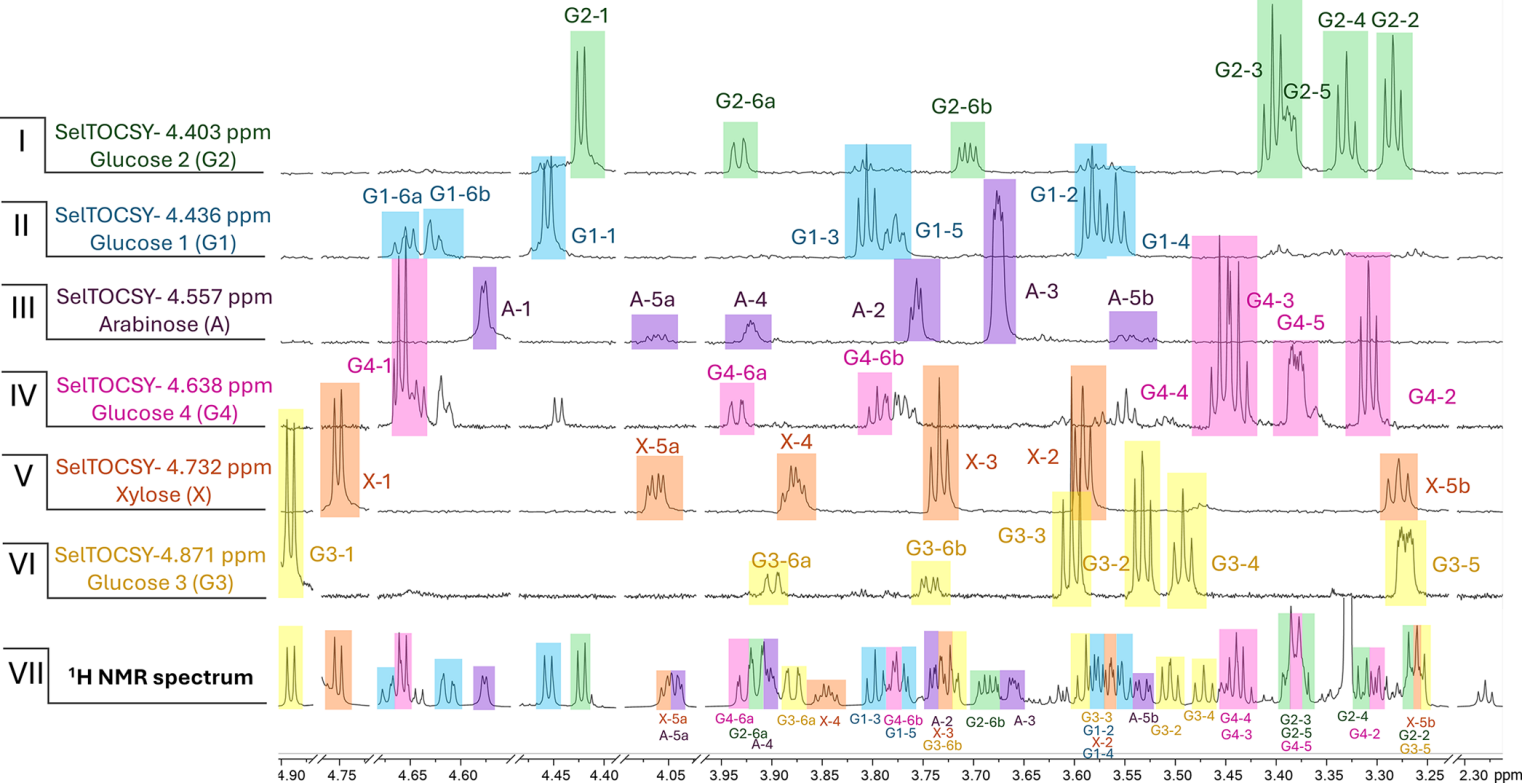
Identification of the six sugars of **1** by 1.1 GHz ^1^H NMR spectroscopy. Shown are the stacked spectra of 1D selective TOCSY experiments for the anomeric protons (I–VI, 120 ms mixing time) of the six sugars and mapping of the assignments to the full ^1^H NMR spectrum of **1** (VII). The TOCSY-based isolation of the individual sugar spin systems combined with HifSA analysis enabled the determination of all coupling constants, which identified four glucoses (I, II, IV, and VI), one xylose (V), and one arabinose (III) unambiguously.

The linkage of the sugars and the location of the second cinnamate residue were determined via HMBC analysis (Figure 6). The first glucose (G1) showed four connection points identified via HMBC: first, the anomeric proton at 4.436 ppm (G1-1) correlated with C-3 of the aglycone (δ_C_ = 91.1 ppm). The second substitution was observed in position G1-6, where the methylene protons (4.649 and 4.594 ppm) correlated with the carboxylic carbon of the second cinnamate residue (167.98 ppm). Further, the second glucose (G2) HMBC cross-peaks between G2-1 (δ_H_ = 4.403 ppm) and G1-4 (δ_C_ = 81.45 ppm) proved their β-1→4 linkage, while the xylose (X) was connected to G1 via a β-1→2 bond, as evidence from the correlation between G1-2 (δ_C_ = 82.40 ppm) and X-1 (δ_H_ = 4.732 ppm). The xylose was also multiply substituted, exhibiting linkages with the arabinofuranose (A) in position 4 and with C-3 of the third glucose (G3-3). The observed HMBC cross peaks between 4.557 ppm (A-1) and X-4 (δ_C_ = 72.37 ppm) as well as G3-1 and X-3 (δ_H_ = 4.871 ppm; δ_c_ = 83.78 ppm) confirmed these linkages. Finally, the HMBC correlation between 4.638 ppm (G4-1) and 84.56 ppm (G3-2) proved the β-1→2 linkage between the third and fourth glucose, thereby forming a sophorose substructure. Collectively, this identified **1** as the 3-*O*-hexaglycoside of a 21-cinnamoyloxy-oleanolic acid containing β-Glc*p*-{(2→1)-β-Xyl*p*-[(4→1)-α-Ara*f*]-(3→1)-β-Glc*p*-(2→1)-β-Glc*p*}-6-*O*-cinnamoyl,(4→1)-β-Glc*p* as the oligosaccharide. Figure 1 shows the key HMBC correlations that prove the interglycosidic and ester linkages in **1**, a new natural product named dicitrioside A_1_.

**Figure 6:**
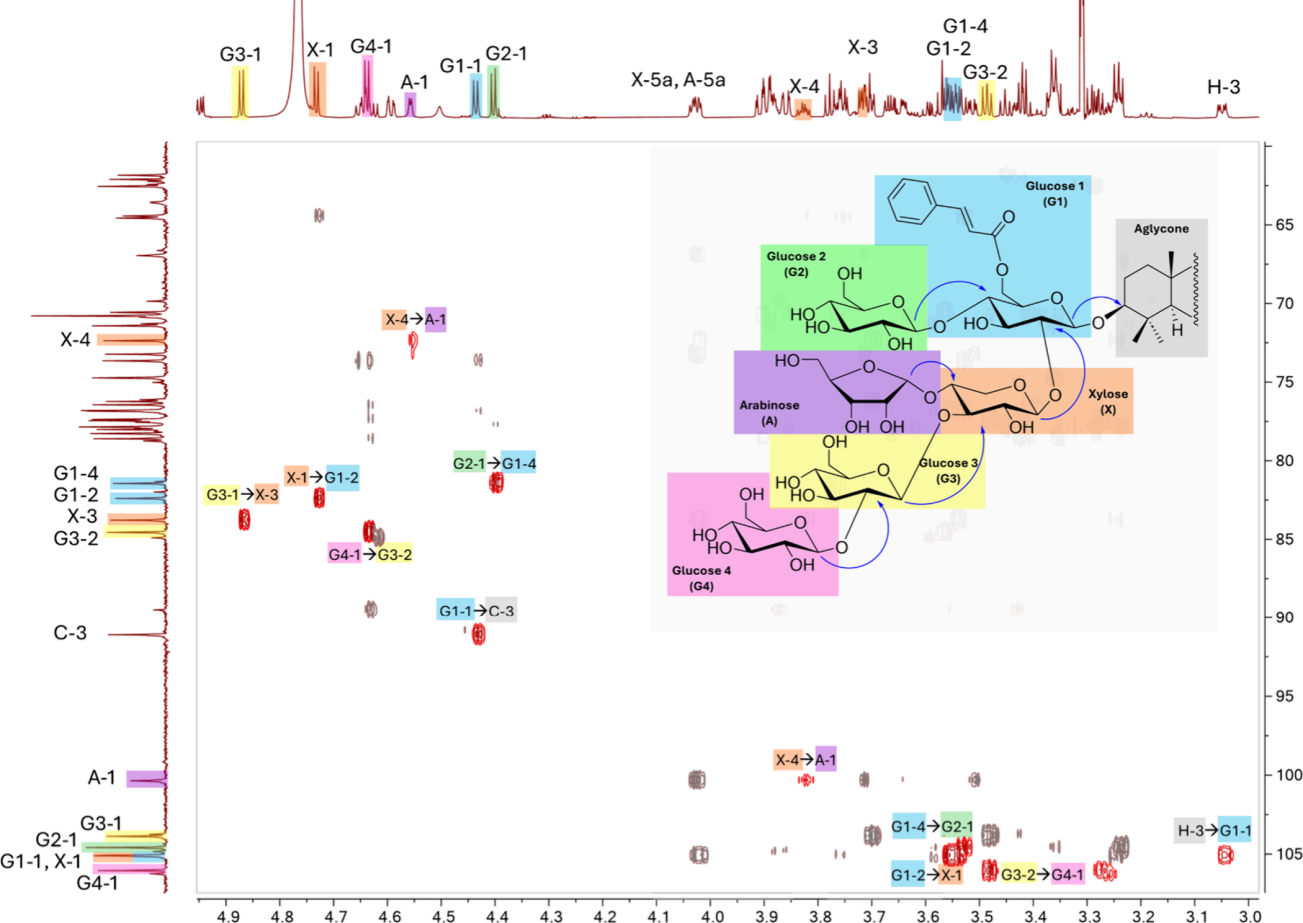
Key HMBC (1.1 GHz ^1^H, 150 MHz ^13^C) correlations for the structural elucidation of the oligosaccharide moiety of **1**. Highlighted in red are HMBC correlations between the anomeric protons of each sugar residue with their respective carbon linkage point. The matching pair of glycosidic correlations, proton-to-carbon from the anomeric H to the attached carbon in the preceding sugar (^3^ *J*_H(anom),C_) plus from the anomeric C to the methine H in the preceding sugar (^3^ *J*_C(anom),H_), were also observed. The only exceptions are a few instances where the dihedral angle resulting from the conformation of the oligosaccharide was near 90° and precluded ^3^ *J*_C(anom),H_ from being measurable.

The SelTOCSY experiments were crucial for elucidation not only of the sugar residues, but also for key positions in the aglycone. The spectra were also foundational for comprehensive assignments of relative stereochemistry by HifSA-based nuclear genotyping of **1**, likely the most complex molecule for which such an analysis has been achieved to date. By listing all H,H-coupling constants and exact chemical shifts, Tables 2 and 3 provide the full ^1^H nuclear genotype^19^ of **1**, which fully explains the highly overlapped spectra via quantum mechanical calculations. These outcomes also demonstrate that HifSA can be applied even to purity limited isolates such as triterpene oligoglycosides.

**Table 2:** ^1^H (1.1 GHz) and ^13^C (150 MHz) NMR Data Including HifSA Profile Parameters for the Aglycone Moieties of **1** and **2**

	1					2			
position	δ_C_	δ_H_	Mult	*J* (in Hz)	HMBC	δ_C_	δ_H_	Mult	*J* (in Hz)
1ax	40.40	0.758	ddd	13.53, −13.07, 3.79		39.29	0.761	ddd	14.15, −12.78, 3.21
1eq		1.367	ddd	–13.07, 3.71, 3.04			1.369	ddd	–12.78, 4.06, 4.33
2ax	27.21	1.584	dddd	13.53, −12.60, 11.67, 3.71		26.10	1.582	dddd	14.15, −12.81, 11.57, 4.06
2eq		1.823	dddd	–12.60, 4.18, 3.79, 3.04			1.822	dddd	–12.81, 4.72, 4.33, 3.214
3	91.10	3.049	dd	11.67, 4.18	23, 24, 1	89.92	3.051	dd	11.57, 4.72
4	40.21					39.16			
5	57.07	0.667	d	12.07	25, 23, 6, 24, 7, 11, 8, 4	55.88	0.675	d	12.00
6ax	19.23	1.335	dddd	–12.99, 12.14, 12.07, 3.51		18.04	1.321	ddd	–13.52, 12.14, 12.00, 3.51
6eq		1.524	ddd	–12.99, 4.06, 2.94			1.531	ddd	–13.52, 4.06, 2.25
7ax	33.94	1.428	ddd	–12.32, 12.14, 4.06		32.80	1.437	ddd	–12.22, 12.14, 4.06
7eq		1.259	ddd	–12.32, 3.51, 2.94			1.267	ddd	–12.22, 3.51, 2.25
8	40.36					39.07			
9	48.86	1.316	ddd	11.14, 6.55, −1.30		47.88	1.351	ddd	11.14, 6.55, −1.30
10	37.80					36.73			
11ax	24.19	1.621	ddd	–17.73, 11.14, 3.28		23.00	1.622	ddd	–17.73, 11.14, 3.28
11eq		1.328	ddd	–17.73, 6.55, 2.25			1.325	ddd	–17.73, 6.55, 2.25
12	124.47	5.124	ddd	3.28, 2.25, −1.30	11, 14, 9	123.32	5.131	ddd	3.28, 2.25, −1.30
13	143.32					142.02			
14	42.73					41.53			
15ax	28.71	1.702	ddd	13.50, −12.55, 4.33		27.55	1.700	ddd	14.28, −13.26, 4.28
15eq		1.071	ddd	–12.55, 4.09, 3.15			1.084	ddd	–13.26, 4.26, 3.24
16ax	25.29	2.079	ddd	13.50, −13.13, 4.09	15, 17	24.14	2.093	ddd	14.28, −12.62, 4.26
16eq		1.792	ddd	–13.13, 4.33, 3.15			1.793	ddd	–12.62, 4.28, 3.24
17	49.14					47.90			
18	42.07	2.951	dd	14.28, 4.99	16, 14, 19, 17	40.91	2.953	dd	13.83, 4.99
19ax	47.90	1.959	dd	14.28, −14.13	30, 29, 20, 18, 21	46.76	1.971	dd	–14.46, 13.83
19eq		1.390	dd	–14.13, 4.99			1.399	dd	–14.46, 4.99
20	36.35					35.23			
21	76.84	4.949	dd	11.94, 4.89	30, 29, 20, 22	75.53	4.955	dd	11.93, 4.85
22ax	37.91	1.770	dd	–12.77, 11.94		36.67	1.771	dd	–13.17, 11.93
22eq		1.828	dd	–12.77, 4.89			1.834	dd	–13.17, 4.85
23	16.82	0.804	s		24, 4, 5	15.62	0.808	s	
24	28.34	1.033	s			27.08	1.038	s	
25	15.91	0.844	s		11, 4, 8, 9, 5	14.80	0.849	s	
26	17.74	0.736	s		7, 8, 14, 9	16.66	0.740	s	
27	26.13	1.045	s		15, 8, 14	25.00	1.059	s	
28	179.63					178.12			
29	29.43	1.024	s			28.30	1.035	s	
30	18.97	1.153	s		29, 20, 19	17.86	1.162	s	
31	168.01				21	166.69	-		
32	119.02	6.585	d	15.95		117.91	6.611	d	15.94
33	146.29	7.711	d	15.95		144.93	7.720	d	15.94
34	135.71					134.54			
35	129.34	7.651	dd	7.79, 1.23		128.21	7.681	dd	7.50, 1.23
36	130.25	7.430	dd	7.79, 7.43		129.01	7.458	dd	7.50, 7.05
37	131.63	7.437	dd	7.43, 1.23		130.47	7.454	dd	7.05, 1.23

**Table 3:** ^1^H (1.1 GHz) and ^13^C (150 MHz) NMR Data for the Oligosaccharide Moieties of **1** and **2**

	1					2			
position	δ_C_	δ_H_	Mult	*J* (in Hz)	HMBC	δ_C_	δ_H_	Mult	*J* (in Hz)
glucose 1 [β-Glc*p*-1]									
G1-1	105.08	4.436	d	7.81	C-3, G1-5	103.96	4.441	d	7.92
G1-2	82.40	3.558	dd	9.00, 7.81	X-1, G1-3	80.66	3.594	dd	8.97, 7.92
G1-3	76.81	3.77	dd	9.00, 8.69	G1-4, G1-1	75.69	3.763	dd	8.97, 8.60
G1-4	81.45	3.53	dd	9.71, 8.69	G2-1, G1-3, G1–5	80.30	3.538	dd	9.94, 8.60
G1-5	73.65	3.76	ddd	9.71, 8.89, 2.49	G1-4, G1-1	72.46	3.759	ddd	9.94, 8.82, 2.46
G1-6a	64.57	4.64	dd	–11.59, 8.89	G1-5, G1-7	63.35	4.667	dd	–11.62, 8.82
G1-6b		4.59	dd	–11.59, 2.49	G1-7		4.591	dd	–11.62, 2.46
G1-7	167.98				G1–6	166.72			
G1-8	119.22	6.56	d	15.98	G1–9	118.14	6.587	d	15.93
G1-9	146.16	7.69	d	15.98	G1-11, G1-G1-8	145.06	7.709	d	15.93
G1-10	135.74					134.51			
G1-11	129.36	7.65	dd	7.79, 1.23		128.24	7.677	dd	1.28, 7.74
G1-12	130.11	7.42	dd	7.79, 7.43		129.11	7.445	dd	7.28, 7.74
G1-13	131.59	7.44	dd	7.43, 1.23		130.47	7.429	dd	7.28, 1.28
glucose 2 [β-Glc*p*-2]									
G2-1	104.58	4.41	d	7.87	G1-4, G2-2	103.49	4.402	d	7.95
G2-2	74.70	3.23	dd	9.21, 7.87	G2-3, G2-1	73.50	3.238	dd	9.15, 7.95
G2-3	77.83	3.36	dd	9.21, 9.04	G2-4, G2-2	77.57	3.357	dd	9.15, 9.13
G2-4	71.42	3.29	dd	9.81, 9.04	G2-5, G2-6a	70.21	3.291	dd	9.72, 9.13
G2-5	78.27	3.36	ddd	9.81, 6.03, 2.37	G2-6b, G2-4	77.11	3.372	ddd	9.72, 6.02, 2.11
G2-6a	62.55	3.89	dd	–12.03, 2.37	G2–4	61.25	3.892	dd	–11.76, 2.11
G2-6b		3.67	dd	–12.03, 6.03	G2–4, G2–5		3.665	dd	–11.76, 6.02
xylose [β-Xyl*p*]									
X-1	105.09	4.73	d	7.54	G1-2, X-5a	103.55	4.755	d	7.62
X-2	76.23	3.54	dd	9.08, 7.54	X-3	88.33	3.436	dd	8.74, 7.62
X-3	83.78	3.70	dd	9.08, 8.78	X-2, X-4, G3-1, X-5a	73.78	3.432	dd	8.74, 8.22
X-4	72.37	3.83	ddd	9.55, 8.78, 5.21	X-3, X-5a, A-1	68.63	3.563	ddd	9.99, 8.22, 5.46
X-5a	64.43	4.01	dd	–11.92, 5.21	X-1, A-1	65.46	3.881	dd	–11.80, 5.46
X-5b		3.26	dd	–11.92, 9.55			3.195	dd	–11.80, 9.99
arabinose [α-Ara*f*]									
A-1	100.36	4.56	d	4.30	X-4, A-2				
A-2	70.79	3.71	dd	6.47, 4.30	A-3, A-4				
A-3	73.22	3.64	dd	6.47, 3.04	A-5b				
A-4	66.95	3.89	ddd	7.04, 3.94, 3.04	A-5b, A-2				
A-5a	63.57	4.03	dd	–11.89, 7.043	A-1, A-3				
A-5b		3.51	dd	–11.89, 3.94					
glucose 3 [β-Glc*p*-3]									
G3-1	103.87	4.87	d	7.84	X-3, G3-2, G3-6b	102.51	4.641	d	7.83
G3-2	84.56	3.49	dd	9.35, 7.84	G3-3, G4-1	83.69	3.483	dd	9.20, 7.83
G3-3	77.45	3.57	dd	9.35, 9.09	G3-4, G3-2	76.25	3.598	dd	9.31, 9.20
G3-4	70.56	3.44	dd	9.88, 9.09	G3-6a	70.09	3.322	dd	9.60, 9.31
G3-5	78.01	3.24	ddd	9.88, 4.55, 2.56	G3-6a	76.77	3.362	ddd	9.60, 4.47, 2.32
G3-6a	61.84	3.85	dd	–12.73, 2.56	G3-4, G3-5	61.33	3.872	dd	–12.05, 2.32
G3-6b		3.7	dd	–12.73, 4.55			3.616	dd	–12.05, 4.47
glucose 4 [β-Glc*p*-4]									
G4-1	106.04	4.64	d	7.75	G3-2, G4-2	105.05	4.625	d	7.87
G4-2	76.44	3.27	dd	9.54, 7.75	G4-4, G4-1	74.93	3.259	dd	9.42, 7.87
G4-3	77.37	3.42	dd	9.54, 9.03	G4-4, G4-5	76.38	3.390	dd	9.92, 9.42
G4-4	70.79	3.42	dd	9.77, 9.03	G4-3, G4-6a	69.68	3.368	dd	9.92, 9.50
G4-5	78.58	3.36	ddd	9.77, 4.46, 2.21	G4-3, G4-6a	76.72	3.353	ddd	9.50, 4.50, 2.07
G4-6a	62.12	3.90	dd	–12.20, 2.21	G4-4, G4-5	60.99	3.883	dd	–11.99, 2.07
G4-6b		3.75	dd	–12.20, 4.46			3.722	dd	–11.99, 4.50

### Structural Elucidation of Dicitrioside B_1_ (**2**)

Rel-qHNMR analysis identified **2** as the main component 61% in isolate T23, which also contained 21% of **1** plus 15% of an unknown congener and 2% of fatty/aliphatic residue. HRMS yielded an *m*/*z* value of 1512.6929 corresponding to the molecular formula C_77_H_108_O_30_. The NMR spectra of **2** were nearly identical with those of **1**. Congruent aliphatic ^1^H fingerprints indicated the same cinnamoylated aglycone, and the presence of five anomeric protons suggested the structural difference to reside in the oligosaccharide portion of **2**. This prompted SelTOCSY experiments, which upon excitation of the anomeric protons identified four glucose and one xylose residues (Figure S16). HMBC correlations established the interglycosidic connectivities: the anomeric proton at 4.441 ppm (Glc-1) connected with C-3 of the aglycone (89.9 ppm). The second glucose (Glc-2) correlated with position 4 of Glc-1, indicating a β-1→4 linkage. As in **1**, **2** exhibited a β-1→2 linkage between the Xyl and Glc-1.

While a sophorose moiety was also present in **2**, HMBC revealed that the Glc-3 to Xyl linkage was β-1→2. This subtle branching difference to **1** [β-Xyl*p*-(3→1)-β-Glc*p*] and the absence of the Ara*f* moiety attached to C-4 of xylose in **1** were the main differences in the oligosaccharide residue of **2**, which was determined as β-Glc*p*-[(2→1)-β-Xyl*p*-(2→1)-β-Glc*p*-(2→1)-β-Glc*p*]-6-*O*-cinnamoyl, (4→1)-β-Glc*p*. Figure 1 shows the key HMBC correlations for assembling the main moieties of **2**, a new compound named dicitrioside B_1_. Further support came from 1.1 GHz HifSA analysis, which enabled the unambiguous interpretation of all NMR data as shown in Tables 2 and 3. In addition to carrying unprecedented sugar sequences, both compounds have two unique features that stand out from the triterpenoid literature: first, the double cinnamoyl residues, distributed between the aglycone and oligosaccharide portion. Second, the phenylpropanoyl substitution in position 6 of the sugar attached to the aglycone.

### Antiviral Activity of the Dicitriosides **1** and **2**

The highly active fractions F21–F25 were pooled and evaluated via HEKSA to determine the half-inhibitory dilution of the mixture showing an IC_50_ of 1/24,480 (Figure 3G). Considering the molecular weight (MW) of the main components, as well as the ratio (1:1) observed in the UHPLC profiles between **1** and **2**, the MW of both compounds were averaged (1578 g/mol). Using these calculations, the IC_50_ can be transformed into IC_50_= 530 nM (linear regression 95% CI= 500–540 nM). This shows the potency of the active components by comparing the IC_50_ previously calculated for IQC90 (12.8 μM), showing that the triterpene glycosides are 25-fold more active.

An important consideration in the bioactivity context is the often-neglected difference between molar- and weight-based impurity designations: in the (frequently encountered) absence of MW information, the initial rel-qHNMR analysis of IQC90 had to assume isobaric impurities to derive the value of ∼0.80% for the designated triterpene glycoside impurities. Now knowing their much larger size (average MW: 1578 g/mol) served as a reminder that the ∼0.80% had to be interpreted as mol % and, thus, required recalculation for expression as the more customary mass percentage value: at a 3.4-fold higher MW relative to IQC (464 g/mol), minor triterpene glycosides constituted 2.68% (m/m) of IQC90.

Triterpenoid oligoglycosides with 21-cinnamoyloxy-oleanolic acid aglycone have been isolated previously: the pachyelasides from *Pachyelasma tessmannii*, a widely distributed tree in central Africa;^21^ the anti-influenza congeners from *Burkea africana* bark;^22^ cytotoxic saponins from *Enterolobium contortisiliquum*;^18,23,24^ and saponins from *Mimosa pigra*.^25^ However, these compounds differ substantially in sugar identities and linkage patterns of their oligosaccharide moieties, and they lack the glycosidic cinnamoyl substituent. Structurally different, some widely studied saponins have been described to have anti-SARS-CoV-1 properties, including ginsenoside Rb1 (IC_50_ 100 μM), α-hederin (IC_50_ 10 μM), soyasaponins (IC_50_ 20 μM),^26^ and glycyrrhizin (EC_50_ > 300 μM).^26,27^

The previous and our new findings indicate the general potential of triterpene glycosides as antiviral agents. To prove the selectivity of **1** and **2**, we included the triterpenes ginsenoside Rg1, glycyrrhetinic acid, glycyrrhizin, oleanolic acid, and the *ent*-kaurane, diterpene rebaudioside A, in the HEKSA evaluation (Figure S17), using IQC90 as a positive control. While IQC90 inhibited syncytialization at 25, 50, and 100 μM, and oleanolic acid at 50 and 100 μM, the other saponins were inactive (Figure S17A). Moreover, syncytialization inhibition by oleanolic acid differed from that by IQC90: whereas IQC90-treated cells became small at 20 h post-transfection, yielding individual and more fluorescent events, oleanolic acid-treated cells grouped into highly fluorescent clusters, giving less counts of fluorescent events (Figure S17B). Additional fluorescent and brightfield microscopy confirmed the morphological difference between cells treated with IQC90 vs oleanolic acid (Figure S17C,D).

### Purity–Activity Relationships of the Dicitriosides **1** and **2**

The chromatographic profiles of fractions F21 to F27 (Figure S15) showed the presence of two main compounds, primarily present in fractions T21 and T23 and identified as **1** and **2**, respectively. However, biosynthetic considerations (explaining the combinatorial complexity of triterpenoid glycosides metabolomes) and the UHPLC profiles of the 7-m Sephadex LH-20 fractions indicated that these bis-cinnamoylated triterpenoid oligoglycosides occur as a complex mixture of congeners with very similar physicochemical and structural properties. To confirm the key role of **1** and **2** as antiviral bioactives in the non-IQC components of IQC90, a preliminary purity-activity experiment was performed using HEKSA.

Taking into account both sample limitations and the need for high chromatographic resolution, separation of **1** and **2** was performed by UHPLC using fraction T22, yielding five fractions I–V. Fractions T22–II-V contained **1**, **2**, and two other triterpenoids in different ratios (Figure 7A), while fractions I and V contained unidentified minor components outside the peaks of interest. Table S1 shows the purities of the target compounds calculated based on areas in the chromatographic profiles. HEKSA showed activity in fractions T22-II and T22-III. Chemically, T22-II contained 84% of **1** and 14% of compound **2**, whereas T22-III consisted of 6% of **1** and 94% of **2**. Bioactivity of T22-II was strongly present until the fourth consecutive dilution (1/800, 8.3 μgE IQC90), with a very minor activity in the following dilution; whereas for T22-III, bioactivity persisted strongly until dilution 1/3,200, translating into 4.2 μgE IQC90 (Figure 7C). These findings confirm that the two minor triterpenoids are the major bioactives, and that dicitrioside B_1_ (**2**) is more active than dicitrioside A_1_ (**1**). The quantitative bioactivities were confirmed qualitatively by fluorometry as well as microscopy (Figure 7B). The activity observed for both fractions reflects the contribution of these two major components of the very minor (<1 mol %) triterpenoid impurity complex. However, attempts to completely isolate compound **1** were unsuccessful; therefore, the activity measured for this compound is limited by its purity.

**Figure 7:**
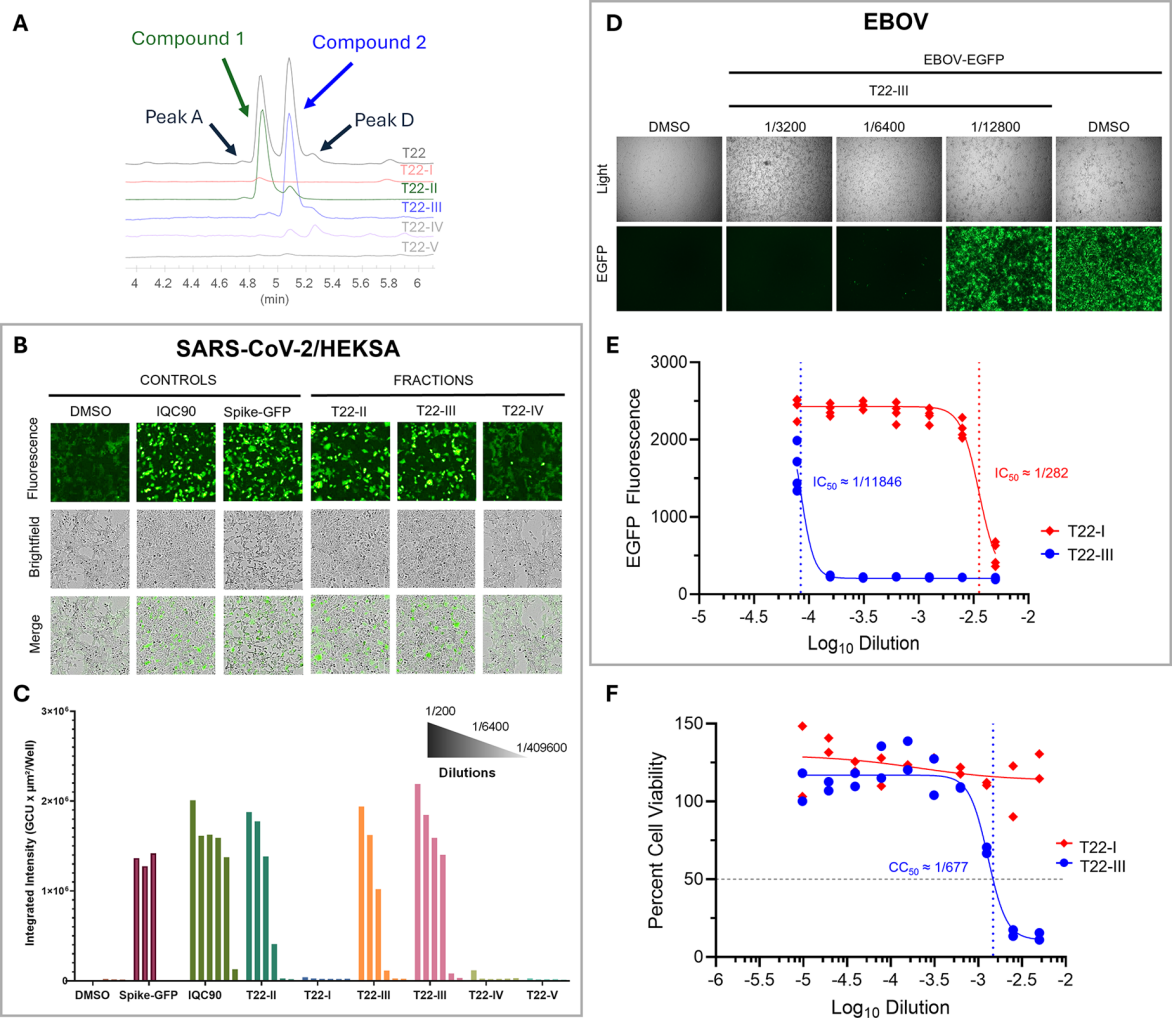
Preliminary purity-activity relationship studies of **1** and **2** in HEKSA, in infected EBOV-EGFP Vero E6 cells, and for cytotoxicity. (A) UHPLC chromatograms at UV 254 nm showing the relationship between fraction T22, and the subfractions T22–I-V, with **1** isolated in T22-II and **2** in T22-III. (B) HEKSA microscopy images after 20 h incubation comparing IQC90 and the subfractions T22–III-IV at the highest concentration (1/200 dilution). T22–II-IV reduced the syncytialization plaques most significantly. (C) HEKSA integrated fluorescence (20 h post-transfection; biological duplicate; mean) showed activity for T22-II and T22-III until 1/800 and 1/1,600 dilution, respectively. (D) Representative images (light and fluorescence microscopy) of Vero E6 cells infected with EBOV-EGFP at 7 days postinfection showed T22-III as active until 1/6,400 dilution. (E) EGFP fluorescence was quantified as a measure of EBOV infection (technical triplicates) and the IC_50_ calculation via nonlinear regression, is indicated by dotted lines. (F) Cytotoxicity evaluation (technical triplicates) in Vero E6 cells treated with T22-I and T22-III for 7 days vs DMSO control gave an interpolated CC_50_ value only for T22-III (1/677; dotted line) corresponding to a selectivity index of 17.5.

### Evaluation of Dicitrioside B_1_ (**2**) in EBOV Assays

Fraction T22-III (94% compound **2**) was further evaluated for its ability to inhibit EBOV infection in Vero E6 cells. Cells were treated with increasing dilutions of T22-III, after which they were inoculated with EBOV-EGFP at an MOI of 0.1. On day 7 postinfection, fluorescent microscopy images revealed abundant EGFP signals in cells that were treated with DMSO (drug carrier) only, indicating robust EBOV infection (Figure 7D). Cells treated with T22-III at 1/12,800 revealed a similar magnitude of EGFP fluorescence, while cells treated with 1/6,400 (1.04 μgE IQC90) showed dramatically less EGFP signal, and cells treated with 1/3,200 showed essentially none, similar to uninfected cells. Indeed, quantification of EGFP fluorescence demonstrated significant inhibition of EBOV infection at all dilutions except the highest (1/12,800), with an estimated IC_50_ value of 1/11,846 (0.56 μgE IQC90; Figure 7E). The CC_50_ of T22-III was estimated to be 1/677 (Figure 7F), giving an SI of ∼17.5. Conversely, the control, T22-I, showed no inhibitory activity at any dilution except the lowest, likely from traces of **1**, and it also showed no cytotoxicity (Figure 7E-F). The suitability of this assay was once again confirmed using galidesivir, which has been previously shown to inhibit EBOV infection.^28^ The drug inhibited EBOV-driven EGFP fluorescence, with an IC_50_ of 52.7 μM (Figure S1B). It should be noted that obtaining T22-III in the achieved purity (94%) required the use of UHPLC, which severely limited the amount of material available. However, this approach was instrumental in allowing the confirmatory EBOV bioassay that expanded the antiviral proof-of-principle for the dicitriosides to this pathogen.

## Conclusions

The Residual Complexity (RC) phenomenon is unavoidable in NP research and many other key agents used in biomedical studies. The neglect to report the purity and other modifying chemical properties of substances tested in bioassays hides the possible participation of RC and may lead to false positive or false negative results. The biological activity attributed to the designated (labeled) major compound contained in a substance may be caused, instead, by minor components. As presented above, separation of the different layers of RC in a commercial sample of 90% pure isoquercitrin (IQC90) uncovered the hidden presence (∼0.80 mol % equiv. to 2.68% [m/m]) of a whole family of structurally complex bis-cinnamoylated and branched triterpene oligoglycosides (∼1,700 amu) as truly responsible for the antiviral activity.

The presented case is likely not an isolated incident but potentially more commonplace in NP research, especially when involving highly studied NPs in drug discovery programs. The importance of RC goes beyond the essential lack of druggable pharmacophores of Invalid/Improbable/Interfering Metabolic Panaceas (IMPs) and extends to studies of their innate functions.^9,29^

The knockout removal of isoquercitrin (IQC) by CCS, along with the fractionation of the IQC90 components with long-bed Sephadex LH-20, proved the need for orthogonal chromatography when researching complex mixtures, which are ubiquitous in NP research. Important for bioassay-guided fractionation (see above) was the selectivity of HEKSA, which enabled tracking the active components throughout the project and at a micromolar level.

Furthermore, employing *aliquot-based* screening schemes was key for the ability to track the true, most potent bioactives. Using aliquot- rather than the more common concentration-based bioassays, the observed bioactivity enrichment was linear and led to isolates with potencies that explain the original bioactivity of the IQC90 starting material.

The bis-cinnamoylated triterpene hexa- and penta-glycosides, **1** and **2**, respectively, are new and structurally unique. Sufficiently close congeners for SARs are unavailable, and commercial triterpenoid glycosides are too limited in matching structural features. Within these constraints, our preliminary SAR studies selected five terpenoids as the closest possible initial matches: glycyrrhizin contains an oleanane-type aglycone yet is only a diglycoside with two glucuronic acids; it was inactive. While antiviral activity has been described for glycyrrhizin,^27^ when compared to the nanomolar potency of the likely selective **1** and **2**, antiviral potency of glycyrrhizin is marginal (micromolar range), highly dependent on the specific assay used; antiviral activity could not be confirmed for glycyrrhetinic acid and oleanolic acid. These are typical characteristics of IMPs,^9^ which glycyrrhizin, its aglycone glycyrrhetinic acid, and oleanolic acid all are. Of the two aglycones, oleanolic acid showed some inhibitory activity yet with morphological differences, whereas glycyrrhetinic acid showed only incomplete inhibition of syncytialization. Inclusion of the dammarane triterpenoid, ginsenoside Rg1, and the *ent*-kaurane diterpenoid, rebaudioside A, aimed to evaluate the potential role of aglycones in oligoglycosides, but both were also inactive.

We hypothesize that the dicitriosides have a specific, although unknown at this time, mode of action based upon their submicromolar potency and *in vivo* activity. The combination of a 21-*O*-acylated aglycone, and other E-ring modifications, and variation in the oligosaccharide moiety is known to significantly affect biological activity of several saponins. A highly purified fraction of *Quillaja saponaria* saponins (“QS-21”), containing trisdesmosidic octaglycosides, is an essential adjuvant in modern vaccines, such as against shingles, malaria, and respiratory syncytial virus.^30^ Dehydrosoyasaponin-I, a triglycoside from *Desmodium adscendens*, is a nanomolar potent Maxi-K ion channel agonist.^31^ Notably, triterpene E-ring and oligosaccharide acylation in particular have been reported to alter both *in vitro* and *in vivo* activity of camelliasaponins, aescins, and senegasaponins.^32^ Oxidation of the 22-OH of soyasaponin-I to a ketone increased ion agonist activity by 60-fold, indicating that minor structural modifications can have major effects in this compound class. The potential implication of the absolute configuration of the sugars will remain to be established in the future as the sparse isolation yields did not allow such determinations.

Assessment of the bioactivity (discussed above), yield, and purity of the isolates in both SARS-CoV-2 and EBOV bioassays supported the assignment of **1** and **2** as the true bioactives of IQC90: isolate T22-III containing 94% of **2** showed 2-fold higher potency in SARS-CoV-2 syncytialization inhibition relative to T22-II containing 84% of **1**. While only T22-III could be evaluated for EBOV activity due to sample limitations, prominent activity was observed for dilutions down to 1/6,400 (1.04 μgE IQC90), attesting to the potency of this compound in inhibiting EBOV infectious processes.

As true bioactives of IQC90, the dicitriosides **1** and **2** inherit the previously established lead properties of IQC90, making them promising prophylactic agents against SARS-CoV-2 and EBOV, and being more druggable than IQC. Their administration prior to infection of cells or hosts (*c.f.* *in vivo* work with IQC90 against EBOV^4^) delayed the infection in both cases, showing their protective efficacy against the viruses. While the viruses differ in various ways, the possible broad-spectrum antiviral properties of **1** and **2** could inspire the future therapeutic use of these plant constituents, with added promise when considering the safe use of *Quillaja* triterpenoids. Supportive rationales for the translational prospects of the dicitriosides are (i) the achievement of complete recovery of all bioactivity throughout the fractionation steps, and (ii) full correlation of the newly reported *in vitro* outcomes with those of IQC90 used in the earlier *in vivo* studies against EBOV.^4^ Finally, it is important to highlight that neither IQC nor its congeneric flavonoids are the bioactive component. Our results establish the role of **1** and **2** as true carriers of the activity observed with IQC90, a product labeled as “isoquercitrin”.

This study confirms the intricate relevance of RC in NP-based drug and biomarker discovery and provides key methodologies and concepts for its analysis as a means of advancing scientific rigor. While the outcomes may be considered a delayed validation of isoquercitrin, widely used as a key chemical resource, as a prototypical IMP, it should be pointed out that, rather than invalidating IQC-based science per se, material labeled as “isoquercitrin” led to the discovery of a class of potential new antiviral NP leads. This broader outcome not only constitutes an exemplary case of RC, but also serves as a reminder that RC is ubiquitous, in experimental and commercial NPs alike, and deserves attention.

## Experimental Section

### General Analytical Procedures

Analytical UHPLC was performed with a Shimadzu Nexera equipped with a photodiode array detector (SPD-M20A), using an Acquity UPLC BEH C18 1.7 μm, (2.1 × 50 mm) packed column and an elution gradient of 10:90 ACN (0.1% FA)-H_2_O (0.1% FA) to 100:0 ACN (0.1% FA) in 10 min, with a flow rate of 0.5 mL/min. High-resolution LC-MS (HRLCMS) analysis used a Waters Alliance 2695 (Waters Corp., Milford, MA, USA) coupled with a Waters SYNAPT quadrupole/time-of-flight mass spectrometer. The column used for the UHPLC analysis was an XBridge C18, 2.5 μm particle size, 2.0 × 50 mm with a gradient system of 15:85 ACN (0.1% FA)-H_2_O (0.1% FA) to 100 ACN (0.1% FA) in 12 min, at a flow rate of 0.2 mL/min. High-resolution MS data was obtained with an electrospray ionization in negative mode at a scan range of *m*/*z* 400–1,800. Low-resolution LC-MS analysis used a Shimadzu LC-20AD coupled with a LCMS-2020 detector, the analysis was done with a YMC Pack ODS-AQ (C18) 120 Å, 3 μm (150 × 2.0 mm) packed column and a elution gradient of 5:95 ACN (0.1% FA)-H_2_O (0.1% FA) to 100:0 ACN (0.1% FA) in 13.5 min, wth a flow rate of 0.3 mL/min. Column chromatography (CC) was performed on Sephadex LH-20 gel (GE Healthcare), using AceGlass columns and a Lab Alliance series III pump, while a Shimadzu SPD-10A UV detector was used to set a 260 nm wavelength detection. High-speed countercurrent chromatography (HSCCC) was performed on a MIDI hydrodynamic instrument from Dynamic Extractions (Slough, UK) with a 900 mL column volume and 20 mL injection loop. The column PFA tubing had a 4 mm I.D. The distance between the column axis and the central axis of the centrifuge for the columns was 11 cm, while the β value ranged from 0.64 to 0.81.

### Materials

Isoquercitrin samples were purchased from seven different suppliers, referred to as Supplier A to Supplier G. From Supplier A, two types of IQC were obtained (purities given as labeled): IQC 90% and IQC 98%. Supplier B provided both isoquercitrin and quercetin 3-O-β-d-glucofuranoside (IQC-SB-2) 99.6%. Suppliers C and E provided IQC materials with 99% purity; Suppliers D, F, and G each with 98%, 97.7%, 99.7% purity, respectively. The names of the suppliers of the investigated IQC materials will be made available on request by the corresponding authors.

The other compounds stemmed from an in-house collection: oleanolic acid (97.9%) was originally from ChromaDex (Los Angeles, CA, USA); glycyrrhetinic acid (98%) from Quality Phytochemicals LLC (NJ, USA); glycyrrhizin (80%) from Sigma-Aldrich (Merck KGaA, Darmstadt, Germany), rebaudioside A (96.9%) from the United States Pharmacopoeia (Rockville, MD, USA), and ginsenoside Rg1 (84.7%) from HWI Analytic GmbH (Ruelzheim, Germany). The ^1^H NMR spectra and LCMS data are provided in the Supporting Information (Figures S39–S48).

The deuterated solvents MeOD and DMSO-*d*_6_ were purchased from Cambridge Isotope Laboratories Inc. (Tewksbury, MA, USA).

### HEKSA

This assay has been described in detail previously.^13^ Briefly, HEK293 cell monolayers in 96-well plates were transfected simultaneously with two expression vectors, 2019-nCov_pcDNA3.1­(+)-P2A-eGFP for expression SARS-CoV-2 Spike protein (MolecularCloud) and pcDNA3.1-hACE2-C9 (Addgene) for expression of human angiotensin converting enzyme 2. After 4 h of incubation, the cells were washed with cell media to remove the transfection medium and then it was replaced with medium containing the compound under test and incubation was resumed for 16 h in an IncuCyte S3 incubator (Essen BioScience) which allows time-lapsed capture of GFP fluorescence and brightfield microscopic images of each well as well fluorescence quantification as Green Calibrated Unit (GCU) x μm^2^/well. Syncytialization is manifested by a regrouping of cells around large cell-less patches and a decrease in overall fluorescence. To compute HEKSA-derived IC_50_ by regression analysis, the concentration of test compound yielding the highest fluorescence (maximal blocking of syncytialization) was given the value of 100; the fluorescence of lower concentrations (partial or no blocking of syncytialization) were expressed as % of the reference value.

### IQC Inhibition of SARS-CoV-2

The evaluation of the inhibitory effect of IQC against SARS-CoV-2 was performed essentially as described previously.^1^ Briefly, IQC was dissolved in DMSO and then diluted to various concentrations in treatment medium (DMEM or MEM plus 2% heat-inactivated fetal bovine serum (HI-FBS), 1% l-Glutamine, 1% penicillin/streptomycin (P/S). Vero E6 or Caco-2 cells were cultured in triplicate or quadruplicate in 24-well plates to 80–90% confluency, at which point growth medium was removed and replaced with treatment medium. After a 1 h incubation, cells were infected with SARS-CoV-2 (SARS-CoV-2/Canada/ON-VIDO492 01/2020; GISAID accession # EPI_ISL_425177) at a multiplicity of infection (MOI) of 0.1. Cells were incubated at 37 °C and 5% CO_2_ for 48 h, after which supernatants were harvested and both virus RNA and infectious virus was quantified, as previously described.^13^

### Evaluation of IQC Efficacy in Hamsters

Twenty (10 female, 10 male) Syrian golden hamsters (*Mesocricetus auratus*) were purchased from Charles River (Saint Constant, Quebec, Canada). Following 1 week of acclimation, the animals were divided into two groups (n = 10, equal numbers male and female) and treated with either 50 mg/kg IQC-SB or PBS only via oral gavage. Treatment was delivered daily for nine consecutive days, from day −3 to day 5 postinfection. On day 0, all animals were inoculated intranasally with SARS-CoV-2 at a dose of 10^5^ median tissue culture infectious dose (TCID_50_). Following inoculation, animals were observed daily up to day 16 for weight and clinical signs of disease. Oral, nasal, and rectal swabs were obtained on day 2 postinfection, and half the animals in each group were sacrificed on 4 day postinfection to obtain lung tissue samples. Virus RNA was quantified in all swab and tissue samples via RT-qPCR, as previously described.^13^

### IQC, T22-III, and Galidesivir Inhibition of EBOV

IQC and T22-III were dissolved in DMSO and galidesivir was dissolved in water before subsequently being diluted to various concentrations using treatment medium. Because the concentrations of T22-I and T22-III were unknown, these samples were dissolved in DMSO and then serially diluted 2-fold from 1:200 to 1:12,800 in DMEM only. Vero E6 cells at 80–90% confluency in 96-well plates were incubated with treatment medium for 1 h before infection with a recombinant Ebola virus (variant Makona-C07) expressing the enhanced green fluorescent protein (EGFP) (EBOV-EGFP; Ebola virus *H. sapiens* -rec/GIN/2014/Makona-Guekedou-C07 -EGFP) at an MOI of 0.1. At the indicated time points postinfection, EGFP signal was quantified using a Synergy XT microplate reader (BioTek). Images of infected cells were captured using an EVOS fl fluorescent microscope (Invitrogen).

### Cell Viability Assay

Drug cytotoxicity in Vero E6 cells was assessed as previously described.^1^ Briefly, serial dilutions of IQC90, T22-III, or galidesivir were applied to cells at 80–90% confluency in 96-well plates. Cell viability was assessed after 48 h of IQC90 treatment or 7 days of T22-III or galidesivir treatment using the CyQUANT XTT Cell Viability Assay kit (ThermoFisher) according to the manufacturer’s directions.

### Biosafety and Animal Ethics Statement

All work with infectious SARS-CoV-2 was performed in either the containment level 3 (CL-3) or CL-4 facilities at the Canadian Science Centre for Human and Animal Health (CSCHAH), National Microbiology Laboratory (NML), Public Health Agency of Canada (PHAC) in Winnipeg, Canada. All work with EBOV was performed in the CL-4 facilities at the CSCHAH. All animal experiments were reviewed and approved by the CSCHAH animal care committee in accordance with the guidelines of the Canadian Council on Animal Care. All procedures were conducted in accordance with standard operating protocols appropriate for these levels of biosafety.

### Statistical Analyses

Statistical figure components were generated in GraphPad Prism Version 10. The half-maximal inhibitory concentrations (IC_50_) and half-maximal cytotoxicity concentrations (CC_50_) were calculated using nonlinear regression, with a four-parameter variable slope.

### Aliquot-Based and Bioassay-Guided Isolation of Active Compounds

A 10.9-g sample of IQC90 was fractionated with HSCCC using the solvent system HEMWAT 1:7:1:7 in ascending mode (normal phase) at a flow rate of 20 mL/min and an rpm of 1,100. The stationary phase retention volume ratio was 0.83. The resulting CCC fractions (160 × 20 mL each) were pooled into six subfractions (A, B1, B2, B3, B4 and C). Fraction B1 (60 mg) was further fractionated with a 7-m Sephadex LH-20 system, using MeOH as the mobile phase at a flow rate of 0.8 mL/min. From this procedure, 100 subfractions were obtained and tested with the bioassay. Active fractions F15 to F31 were analyzed by UHPLC. Fraction F21 (2.26 mg) was analyzed to discover compound **1** as the main constituent of the fraction. Fraction F23 (2.05 mg) contained compound **2** as the main constituent of the active fraction.

The *aliquot-based* approach is generally focused on the aliquot of the original bioactive material, in this case IQC90. For this approach, the initial material (in mg) used for the experiments and the number of fractions were key to the calculation. In this case, the first step consisted of a seed of IQC90 of 100 mg, from which 1% of the full amount was sent to the bioassay (HEKSA). As only the fraction B1 was active, the full 100% bioactivity (1 mg equivalent IQC90 = 1 mgE IQC90) is condensed in that fraction. With the mgE and the dilution factors, the calculation of the mgE or μgE was performed. For the following calculations (step 2 and the mini-scale purification of fraction T22), the bioactivity was distributed into the active fractions. As an example, for step 2, the seed material was 3 g, and 5% of the total were used for the bioassays; the active fractions were 9, thus 16.7 mgE IQC90 were present per fraction. Considering the dilution factor, the calculation of the activity of F21 and *F*23 resulted in 2.60 and 0.33 μgE IQC90, respectively. Finally, for the fractionation of T22, 5% of the fraction from the same 3 g of original material were used for the analysis, and five fractions were active, thus 6.7 μgE IQC90 were present in each sample. For this final experiment, the calculations were performed for both HEKSA and EBOV bioassays resulting in 8.3 μgE IQC90 for compound **1**, 4.2 μgE IQC90 for compound **2** in HEKSA, and 1 μgE IQC90 for compound **2** in the EBOV bioassay. The calculations are documented in more detail in the SI.

### NMR Spectroscopy

^1^H NMR spectra were recorded on a 1.1 GHz Bruker Avance Neo spectrometer at NMRFAM, University of Wisconsin Madison. Some additional experiments (COSY, HMBC, HSQC, NOESY, and 1D selective TOCSY) were also performed with this spectrometer. The ^13^C NMR spectra were recorded on a 600 MHz Bruker Avance III equipped with a ^13^C DCH cryoprobe at 298 K. Additional NMR measurements were recorded on a JEOL ECZ at 600 MHz spectrometer equipped with an HFX Royal Probe. The full acquisition parameters are available in the shared raw data. All spectra were recorded in 3 mm NMR tubes using MeOD-DMSO-*d*_6_ mixtures: 9:1 for **1**, 8:2 for **2**, to overcome solubility limitations in small volumes of neat MeOD. The chemical shifts were referenced to the residual protonated solvent (CD_2_HOD, 3.3000 ppm ^1^H, 49.00 ppm ^13^C).

### Quantitative Analysis

Relative quantification (100% method, rel-qHNMR) of the analyzed samples was performed using the following equation:

$$P[\%]=\frac{nInt_{t}\times MW_{t}}{nInt_{t}\times MW_{t}+\sum (nInt_{u}\times MW_{t})}\times 100$$

where MW is the molecular weight, u is the number of impurities, and t is the target analyte. For the highly overlapped samples (T21 and T23), the integrals were obtained via HifSA, all the procedure and calculations are shared as part of the (digital) Supporting Information.

### ^1^H Iterative Functionalized Spin Analysis (HifSA) of 1D ^1^H NMR Spectra

Computational HifSA of the 1D ^1^H NMR spectra of **1** and **2** was performed using the Cosmic Truth software (ctm.nmrsolutions.fi) by NMR Solutions Ltd., Kuopio (Finland). The workflow consisted of importing the spectra as a JDX file and the structures of the compounds (created by Chem3D) as SDF files to generate starting spin parameters. Iterative calculations were performed with the operator until the best fit of the calculated and experimental spectra. The resulting HifSA profiles contained the spin parameters of all analytes (δ, *J*, relaxation response factor, and ω_1/2_ and Lorentzian–Gaussian line shape parameter).

### Dicitrioside A_1_ (**1**)

UV (MeOH) λ_max_ (log ε) 276 (3.06), 222 (2.91), 202 (3.06) nm. HRMS *m*/*z* 1644.7375 [M-H]- (calculated for C_82_H_115_O_34_ 1644.7353, Δ = 1.64 ppm). ^1^H and ^13^C NMR data are presented in Tables 2 and 3.

### Dicitrioside B_1_ (**2**)

UV (MeOH) λ_max_ (log ε) 276 (3.02), 222 (2.85), 202 (2.98) nm. HRMS *m*/*z* 1512.6929 [M-H]- (calculated for C_77_H_107_O_30_ 1512.6925, Δ = −0.126 ppm). ^1^H and ^13^C NMR data are presented in Tables 2 and 3.

## Supplementary Material



## Data Availability

The raw NMR (FIDs) and MS data and associated electronic data including rel-qHNMR spreadsheets are shared via the Harvard Dataverse (DOI: 10.7910/DVN/PTELHW) and nmrXiv.org at DOI: 10.57992/nmrxiv.p163.

## Notes

Additional figures of the biological experiments (cell syncytialization assay [HEKSA], *in vitro* and *in vivo* evaluation of IQC inhibition of SARS-CoV-2 and EBOV, and cytotoxicity); NMR spectroscopy and chromatographical data of the compounds used in this study; information about the aliquot-based bioassay approach (PDF)
