# Supplementary material for: Multilevel Residual Complexity Analysis Reveals Origin of Nanomolar Antiviral Bioactives of “Isoquercitrin”
Source: J Nat Prod. 2026 Apr 10;89(4):1249–64. doi: 10.1021/acs.jnatprod.6c00077 (PMC13122639; doi:10.1021/acs.jnatprod.6c00077)
Supplement: Supplementary file 1 [file np6c00077_si_001.pdf]

■ SUPPORTING INFORMATION

Multi-level Residual Complexity Analysis Reveals Origin of Nanomolar Antiviral Bioactives of “Isoquercitrin”

Daniela Rebollar-Ramos,[1,#] Annie V. Roy,[2,#] J. Brent Friesen,[1,3] Guy Harris,[1] Shao-Nong Chen,[1] Michael Chan,[4] Shihua He,[4] Guodong Liu,[4] Wenjun Zhu,[4] Logan Banadyga,[4,5] James B. McAlpine,[1] Michel Chrétien,[2] Majambu Mbikay,[2*] Guido F. Pauli[1*]

[1]Pharmacognosy Institute and Department of Pharmaceutical Sciences, University of Illinois Chicago, Chicago, Illinois 60612, United States
[2]Functional Endoproteolysis Laboratory, Montreal Clinical Research Institute, Montreal, Quebec H2W1R7, Canada
[3]Physical Sciences Department, Dominican University, River Forest, Illinois 60305, United States
[4]Special Pathogens Program, National Microbiology Laboratory, Public Health Agency of Canada, Winnipeg, Manitoba R3E3R2, Canada
[5]Department of Medical Microbiology and Infectious Diseases, University of Manitoba, Winnipeg, Manitoba R3E0J9, Canada

[#]Contributed equally

*Corresponding authors: gfp@uic.edu, majambu.mbikay@ircm.qc.ca

■ TABLE OF CONTENTS

Figure S1. Galidesivir (BCX4430) inhibits SARS-CoV-2 and EBOV infection.. 4
Figure S2. IQC-SB is not effective against SARS-CoV-2 in Syrian hamsters. 5
Figure S3. HEKSA results for IQC samples from different suppliers.. 6
Figure S4. ^{1}H NMR spectrum of IQC-SB at 600 MHz in MeOD. 7
Figure S5. ^{1}H NMR spectrum of IQC-SB-2 at 600 MHz in MeOD. 7
Figure S6. ^{1}H NMR spectrum of IQC-SC at 600 MHz in MeOD. 8
Figure S7. ^{1}H NMR spectrum of IQC-SD at 600 MHz in MeOD. 8
Figure S8. ^{1}H NMR spectrum of IQC-SF at 600 MHz in MeOD. 9
Figure S9. ^{1}H NMR spectrum of IQC-SG at 600 MHz in MeOD. 9
Figure S10. Inhibition of SARS-CoV-2 replication in Vero E6 and Caco-2 cells for IQC from diverse suppliers. 10
Figure S11. Inhibition of EBOV infection in Vero E6 cells for IQC from diverse suppliers. 11
Figure S12. ^{1}H NMR spectra of the IQC98 (A) and IQC90 (B) samples from Supplier A. 12
Figure S13. LC-MS chromatogram and MS spectra of IQC98. 13
Figure S14. LC-MS chromatogram and MS spectra of IQC90. 14
Figure S15. UHPLC analytical profiles of fractions T20 to T27. 15
Figure S16. Stacked spectra of 1D selective TOCSY experiments and the full ^{1}H NMR spectrum of **2**... 16
Figure S17. Evaluation of a panel of terpenoids in the HEKSA. 17
Figure S18. IQC90 and Triterpenes Glucosides Cytotoxicity on HEK293 Cells over 72 h. 18
Figure S19. ^{1}H NMR spectrum of **1** at 1.1 GHz in MeOD/DMSO-d_6 (9:1). 19
Figure S20. ^{13}C NMR spectrum of **1** at 150 MHz in MeOD/DMSO-d_6 (9:1). 20
Figure S21. 2D COSY NMR spectrum of **1** at 600 MHz in MeOD/DMSO-d_6 (9:1). 21
Figure S22. 2D HSQC NMR spectrum of **1** at 1.1 GHz in MeOD/DMSO-d_6 (9:1). 22
Figure S23. 2D HMBC NMR spectrum of **1** at 1.1 GHz in MeOD/DMSO-d_6 (9:1). 23
Figure S24. 2D HMBC NMR spectrum of **1** at 1.1 GHz in MeOD/DMSO-d_6 (9:1), expansion in the oligosaccharide area. 24
Figure S25. 2D HMBC NMR spectrum of **1** at 1.1 GHz in MeOD/DMSO-d_6 (9:1), expansion in the triterpene area 25
Figure S26. 2D NOESY NMR spectrum of **1** at 1.1 GHz in MeOD/DMSO-d_6 (9:1). 26
Figure S27. 2D NOESY NMR spectrum of **1** at 1.1 GHz in MeOD/DMSO-d_6 (9:1), expansion in the oligosaccharide and triterpene areas. 27
Figure S28. LC-MS chromatogram and MS spectra of fraction F21, containing compound **1** as the main compound. 28
Figure S29. MS/MS fragmentation patterns for compound **1**. 29
Figure S30. ^{1}H NMR spectrum of **2** at 1.1 GHz in MeOD/DMSO-d_6 (8:2). 30
Figure S31. ^{13}C NMR spectrum of **2** at 150 MHz in MeOD/DMSO-d_6 (8:2). 31
Figure S32. 2D COSY NMR spectrum of **2** at 1.1 GHz in MeOD/DMSO-d_6 (8:2). 32
Figure S33. 2D HSQC NMR spectrum of **2** at 600 MHz in MeOD/DMSO-d_6 (8:2). 33
Figure S34. 2D HMBC NMR spectrum of **2** at 600 MHz in MeOD/DMSO-d_6 (8:2). 34
Figure S35. 2D HMBC NMR spectrum of **2** at 600 MHz in MeOD/DMSO-d_6 (8:2), expansion in the

oligosaccharide area. 35
Figure S36. 2D NOESY NMR spectrum of **2** at 600 MHz in MeOD/DMSO-d_6 (8:2) 36
Figure S37. LC-MS chromatogram and MS spectra of fraction F23, containing compound **2** as the main compound. 37
Figure S38. MS/MS fragmentation patterns for compound **2**. 38
Figure S39. ^{1}H NMR spectrum of oleanolic acid at 600 MHz in MeOD. 39
Figure S40. LC-MS chromatogram and MS spectra of oleanolic acid. 39
Figure S41. ^{1}H NMR spectrum of glycyrrhetinic acid at 600 MHz in MeOD. 40
Figure S42. LC-MS chromatogram and MS spectra of glycyrrhetinic acid. 40
Figure S43. 1H NMR spectrum of rebaudioside A at 600 MHz in DMSO-d6. 41
Figure S44. LC-MS chromatogram and MS spectra of rebaudioside A. 42
Figure S45. ^{1}H NMR spectrum of ginsenoside Rg1 at 600 MHz in MeOD. 43
Figure S46. LC-MS chromatogram and MS spectra of ginsenoside Rg1. 43
Figure S47. ^{1}H NMR spectrum of glyzyrrhizin at 600 MHz in DMSO-d6. 44
Figure S48. LC-MS chromatogram and MS spectra of glycyrrhizin. 44
Table S1. UHPLC-based purities of the isolates of **1** and **2** in the subfractions of T22. 45
Table S2. Yields of the antiviral active fractions, T18 to T31. 45

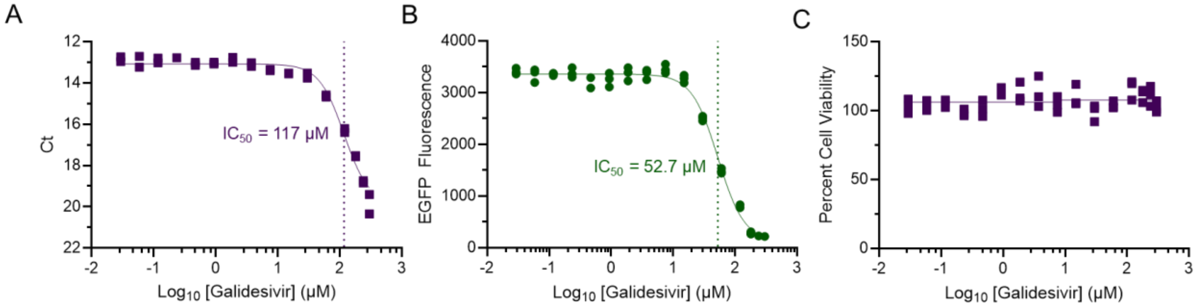

Figure S1. Galidesivir (BCX4430) inhibits SARS-CoV-2 and EBOV infection. A. Vero E6 cells were treated with increasing concentrations (0.03 µM to 300 µM) of galidesivir before they were infected with SARS-CoV-2 at an MOI of 0.01. At 48 hours post-infection with SARS-CoV-2, supernatant was harvested and virus RNA was quantified via RT-qPCR. RNA levels are expressed as Ct (cycle threshold) values on an inverse y-axis; technical duplicates for each condition are shown. **B.** Vero E6 cells were treated with the same concentrations of galidesivir and then infected with EBOV-EGFP at an MOI of 0.1. At 7 days post-infection, EGFP fluorescence was quantified as a measure of EBOV infection; technical triplicates for each condition are shown. **C.** Vero E6 cells were treated with the same concentrations of galidesivir. After 7 days, cell viability was measured and expressed as a percentage of the viability observed in cells treated only with DMSO. Technical triplicates for each condition are shown. Nonlinear regression was used to calculate the IC_{50} value, indicated with a dotted line and corresponding concentration. A CC_{50} value could not be calculated.

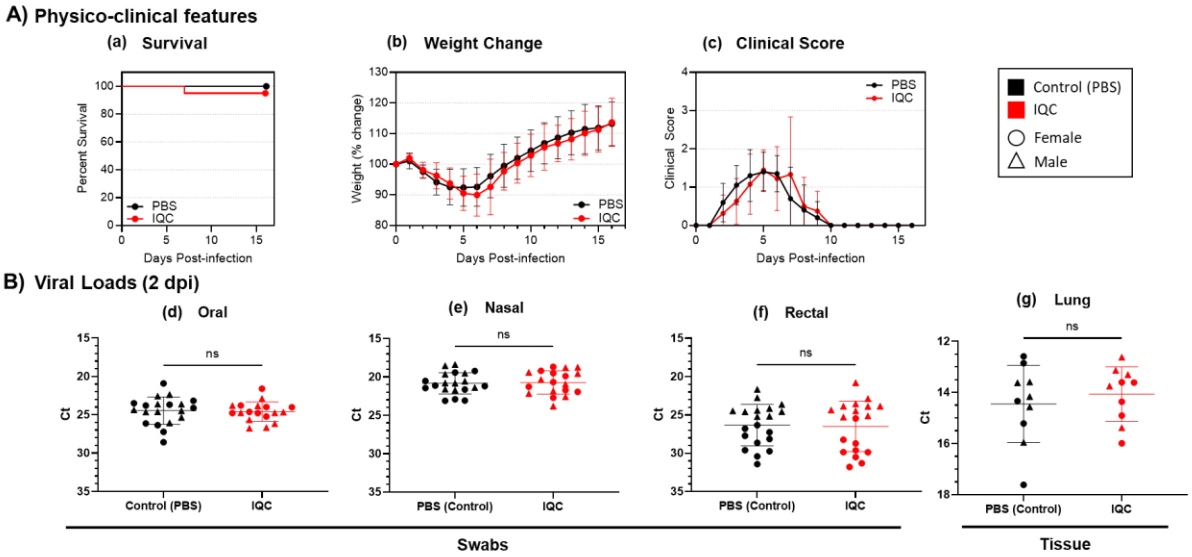

Figure S2. IQC-SB is not effective against SARS-CoV-2 in Syrian hamsters. Ten male and female Syrian hamsters were treated via oral gavage with 50 mg/kg IQC (IQC-SB) from day -3 to day 5 post-infection with 10^5 TCID50 SARS-CoV-2, delivered intranasally. Ten male and female control animals were treated in the same way with PBS only. **A.** Animals were observed for survival (a), weight change (b), and clinical score (c) for 16 days following infection. **B.** Oral, nasal, and rectal swabs were taken on day 2 post-infection, and half the animals were sacrificed on day 4 post-infection, after which lung tissue samples were collected. Viral RNA loads were quantified in all swabs (d-f) and lung samples (g) via RT-qPCR.

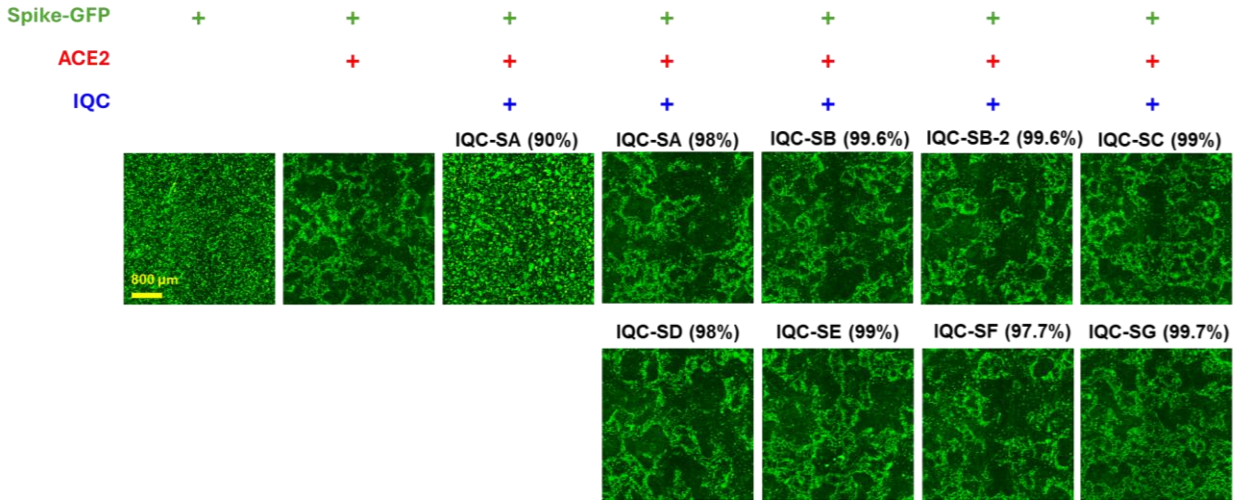

Figure S3. HEKSA results for IQC samples from different suppliers. Fluorescent microscopy images of the HEKSA assay of IQC (100 µM) from different suppliers. Brightness and color adjusted to 65% each. Scale is 800 µm. Untreated cells expressing Spike-GFP alone served as positive controls (no syncytia); untreated cells expressing both Spike-GFP and ACE2 served as negative controls (syncytialized).

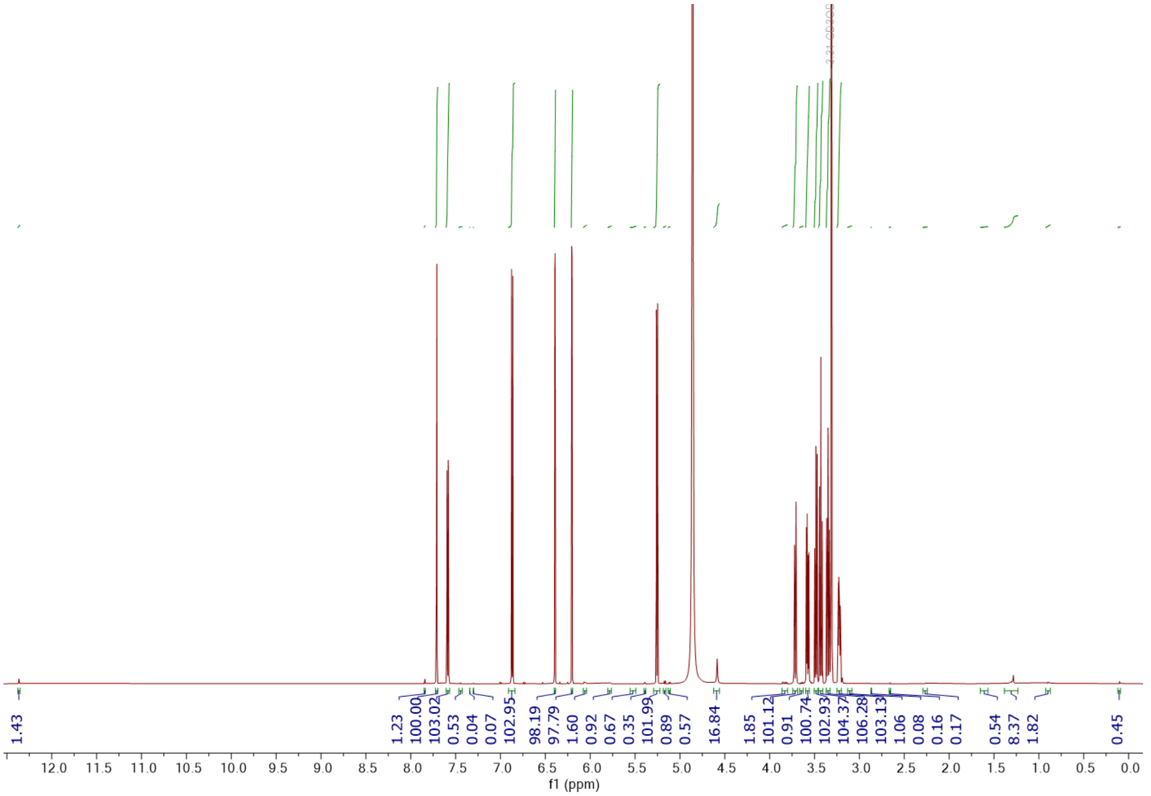

Figure S4. ^{1}H NMR spectrum of IQC-SB at 600 MHz in MeOD.

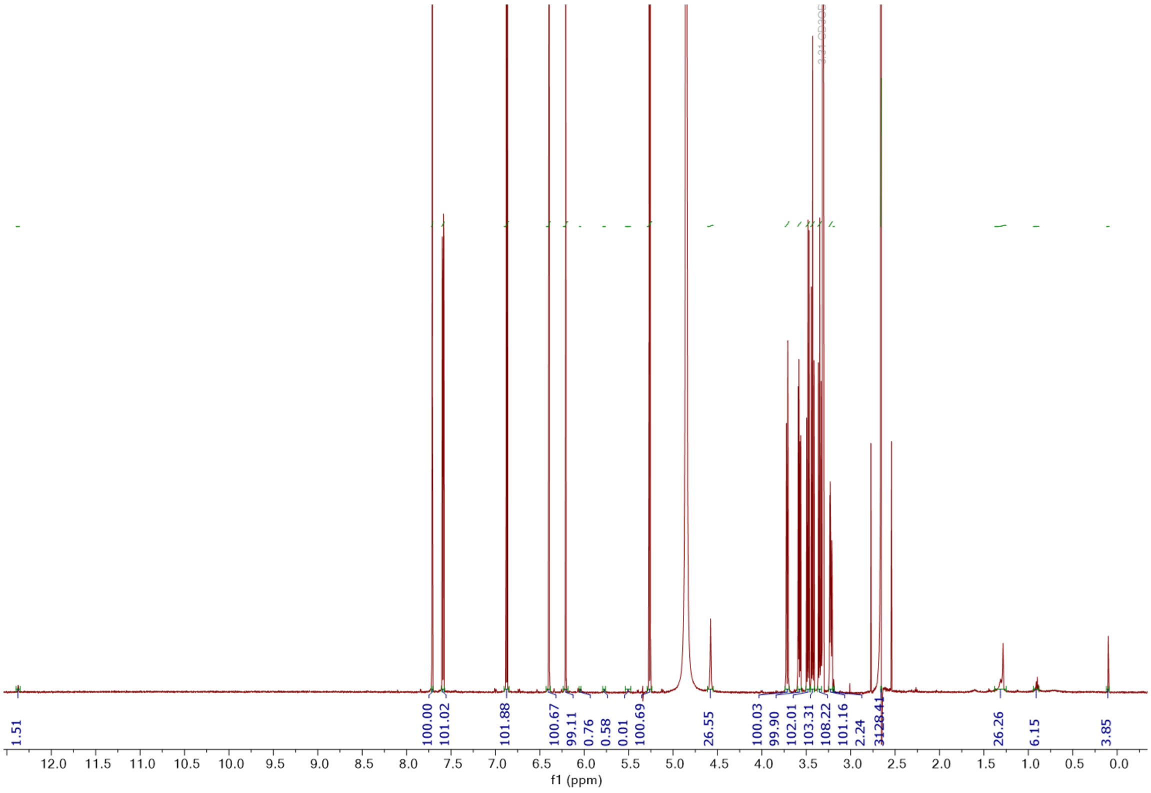

Figure S5. ^{1}H NMR spectrum of IQC-SB-2 at 600 MHz in MeOD.

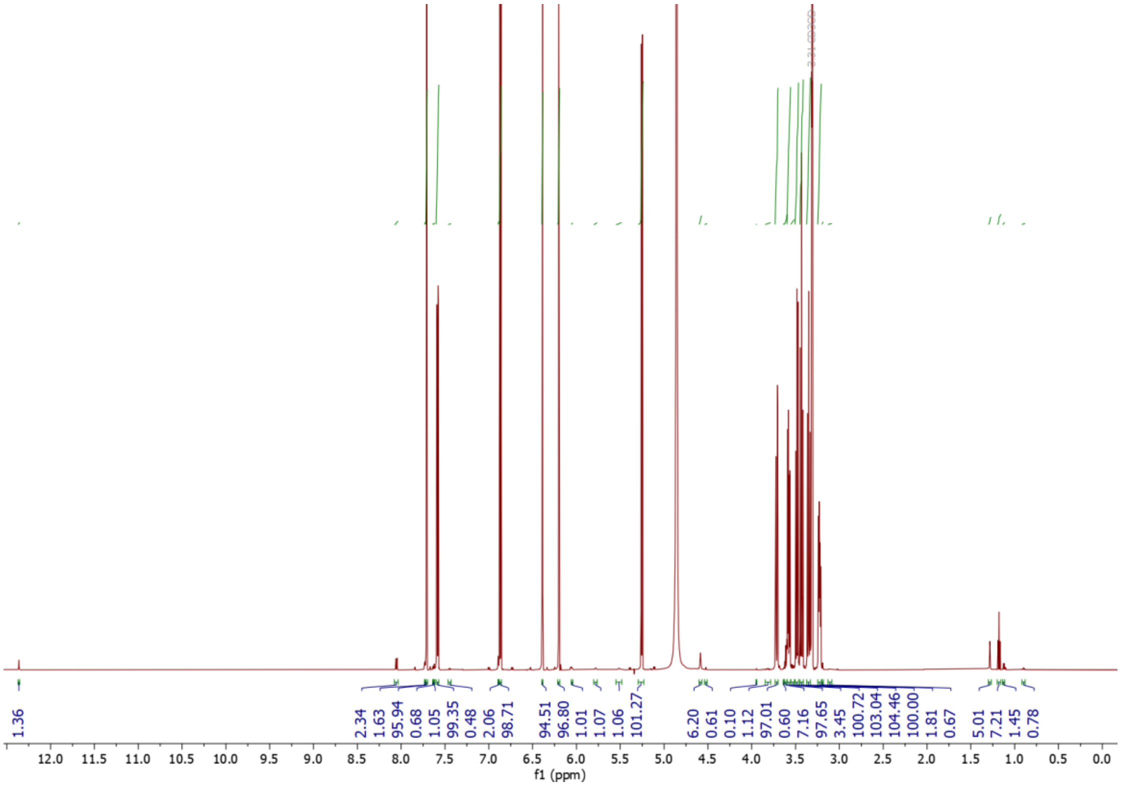

Figure S6. ^{1}H NMR spectrum of IQC-SC at 600 MHz in MeOD.

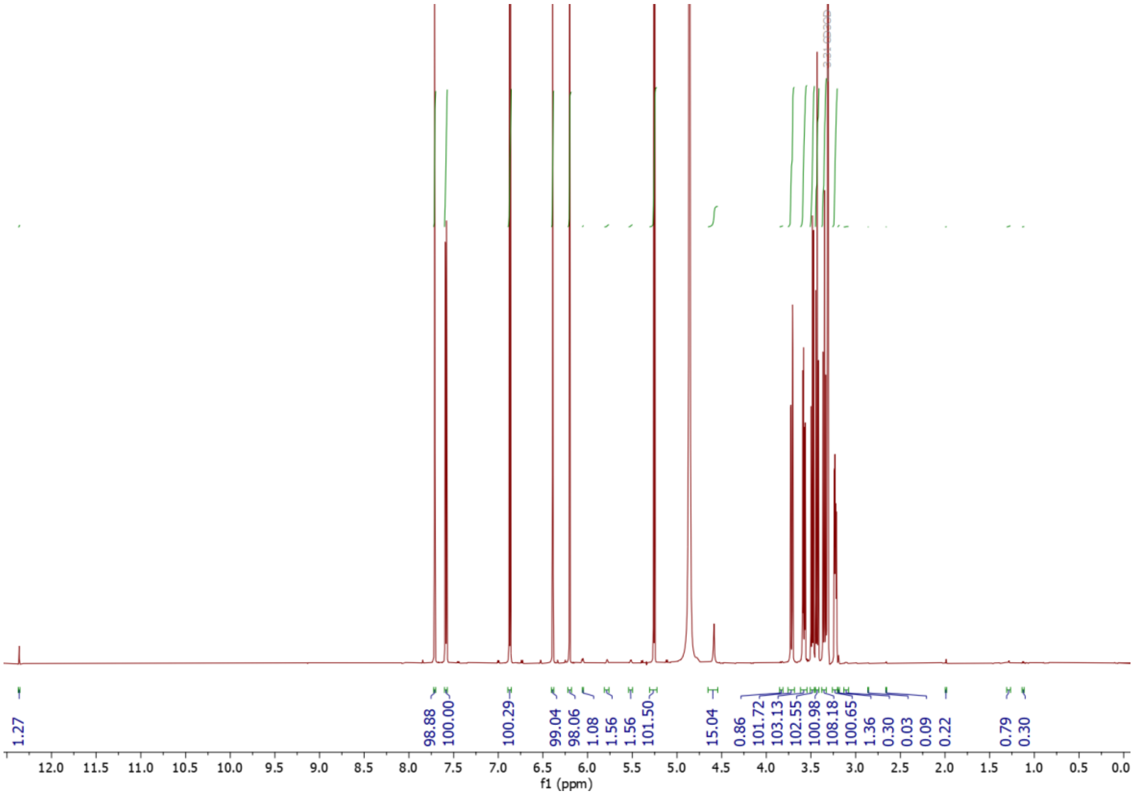

Figure S7. ^{1}H NMR spectrum of IQC-SD at 600 MHz in MeOD.

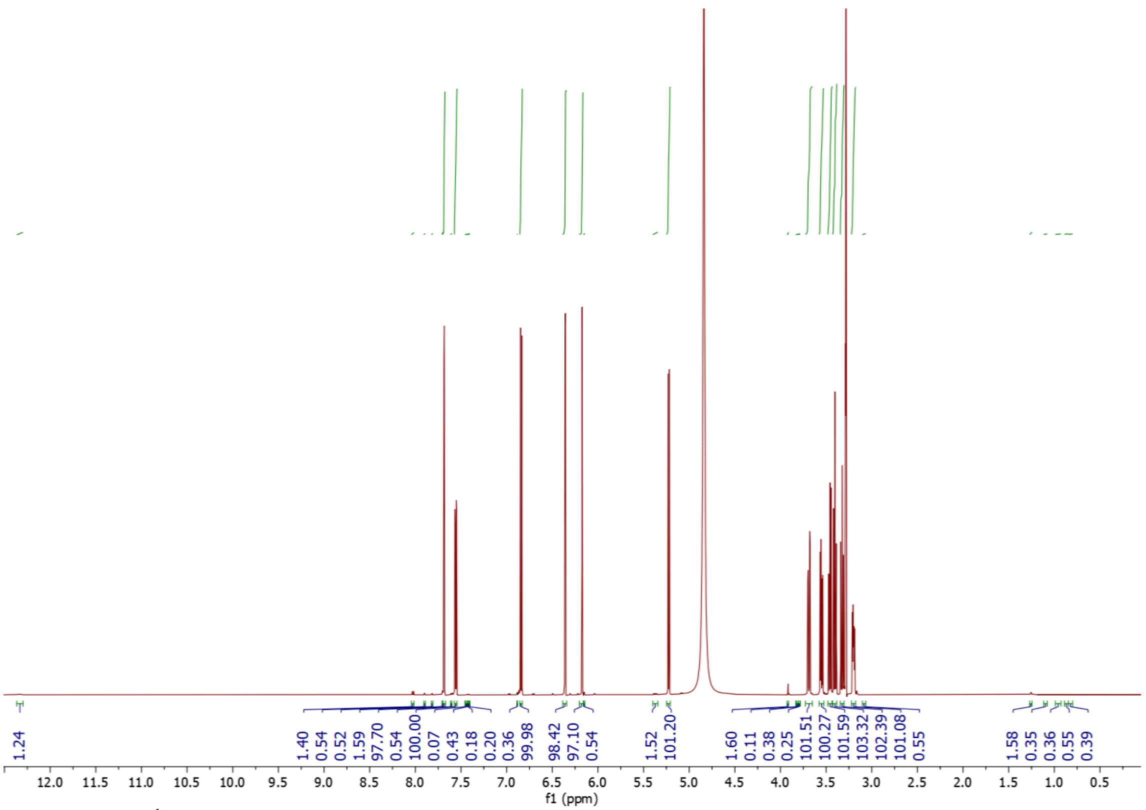

Figure S8. ^{1}H NMR spectrum of IQC-SF at 600 MHz in MeOD.

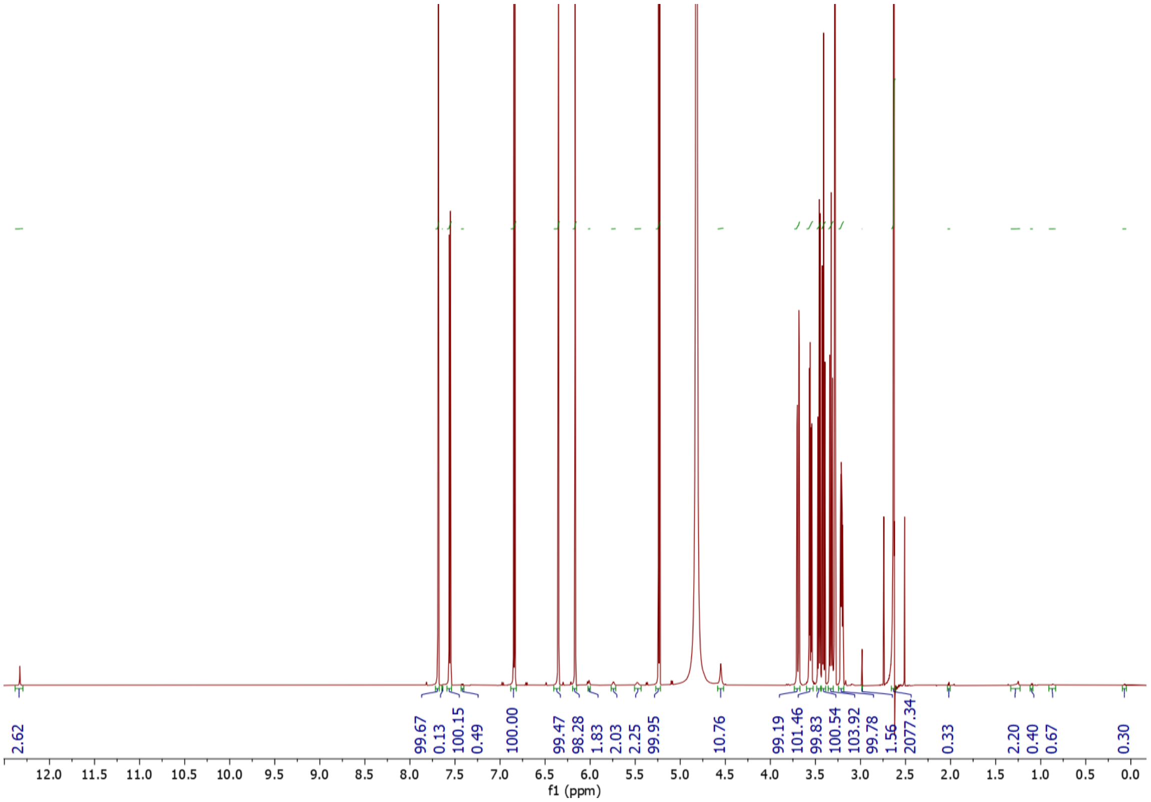

Figure S9. ^{1}H NMR spectrum of IQC-SG at 600 MHz in MeOD.

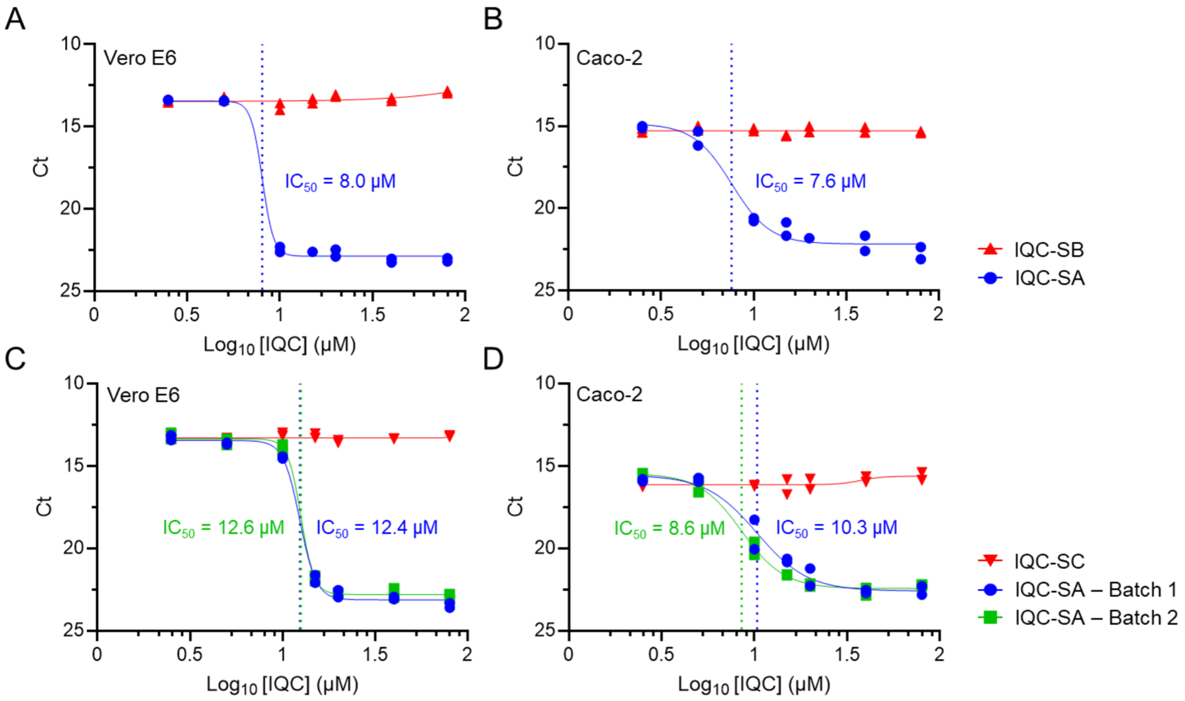

Figure S10. Inhibition of SARS-CoV-2 replication in Vero E6 and Caco-2 cells for IQC from diverse suppliers. Vero E6 (A, C) or Caco-2 (B,D) cells were treated with increasing concentrations of IQC-SA (A-D), IQC-SB (A, B), or IQC-SC (C,D) for 1 hour before they were infected with SARS-CoV-2 at an MOI of 0.1. The final concentrations of IQC were 2.5, 5, 10, 15, 20, 40, and 80 µM. At 48 h post-infection, supernatant was harvested and virus RNA was quantified via RT-qPCR. RNA levels are expressed as Ct (cycle threshold) values on an inverse y-axis; technical duplicates for each condition are shown. Nonlinear regression was used to calculate the IC_{50} values, indicated with a dotted line and corresponding concentration.

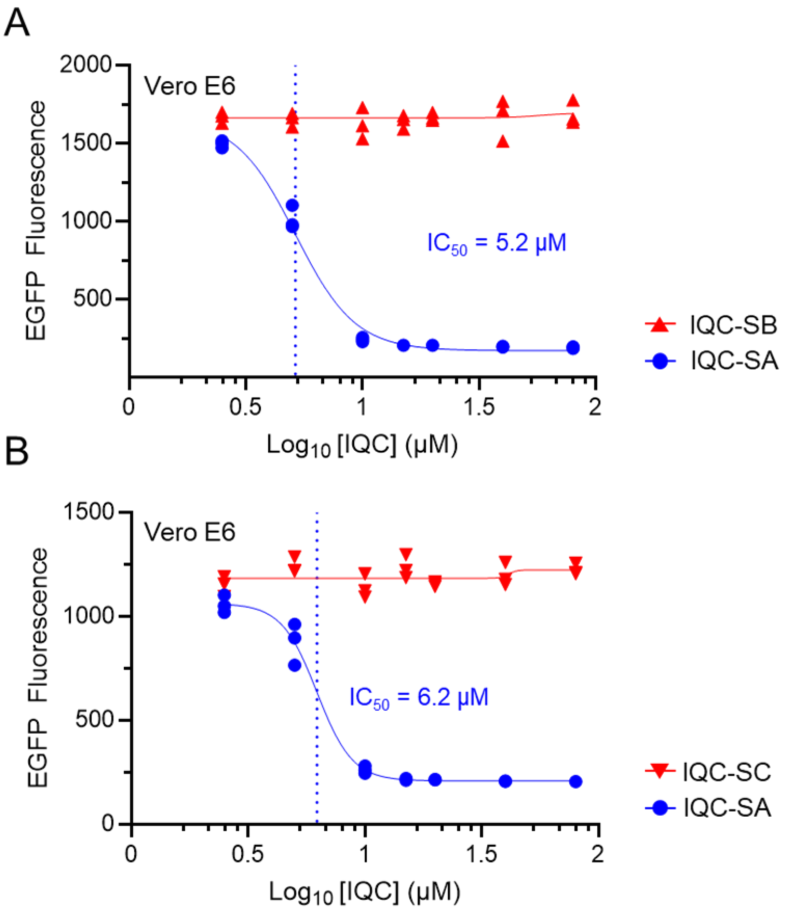

Figure S11. Inhibition of EBOV infection in Vero E6 cells for IQC from diverse suppliers. Vero E6 cells were treated with increasing concentrations of IQC-SA (A, B), IQC-SB (A), or IQC-SC (B) before they were infected with EBOV-EGFP at an MOI of 0.1. The final concentrations of IQC were 2.5, 5, 10, 15, 20, 40, and 80 uM. At 4 days post-infection, EGFP fluorescence was quantified as a measure of EBOV infection. Technical triplicates for each condition are shown. Nonlinear regression was used to calculate the IC_{50} values, indicated with a dotted line and corresponding concentration.

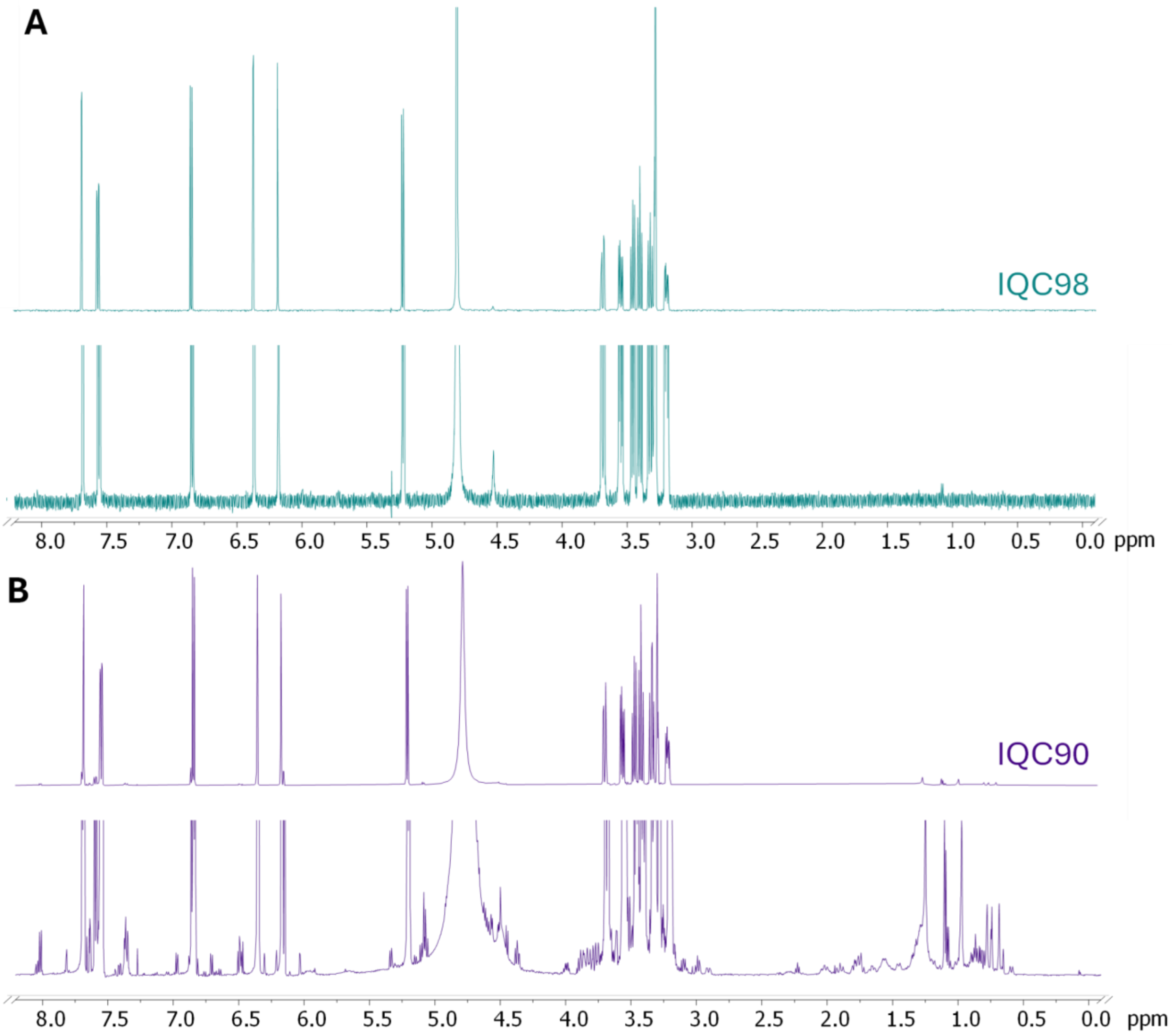

Figure S12. ^{1}H NMR spectra of the IQC98 (A) and IQC90 (B) samples from Supplier A. The overview spectra (upper traces) imply that the materials are relatively pure. However, clear differences are observed when increasing the vertical scale (lower traces). For IQC98 (**A**), the purity is so high that not even a trace of residual complexity is detected with a 10x amplification. In contrast, the amplified IQC90 spectrum (**B**) reveals minor impurities across the entire ppm range, from aliphatic resonances, protons attached to oxygenated carbons, and aromatic/olefinic peak patterns.

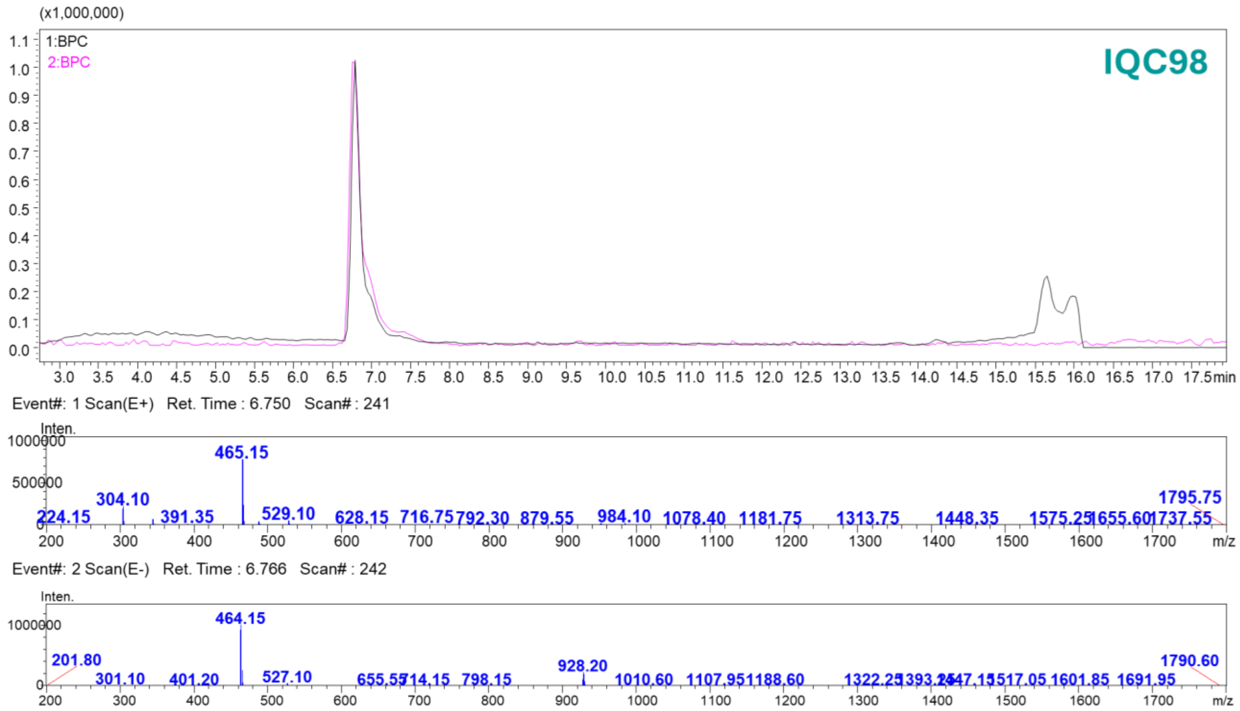

Figure S13. LC-MS chromatogram and MS spectra of IQC98. Base peak intensity chromatogram and low–low-resolution MS data in positive and negative mode of the peak at t_R= 6.75 min. Column: YMC Pack ODS-AQ (C18) 120 Å, 3 µm (150 x 2.0 mm). Elution gradient: 5:95 ACN (0.1% FA)-H_2O (0.1% FA) to 100:0 ACN (0.1% FA) in 13.5 min, flow 0.3 mL/min.

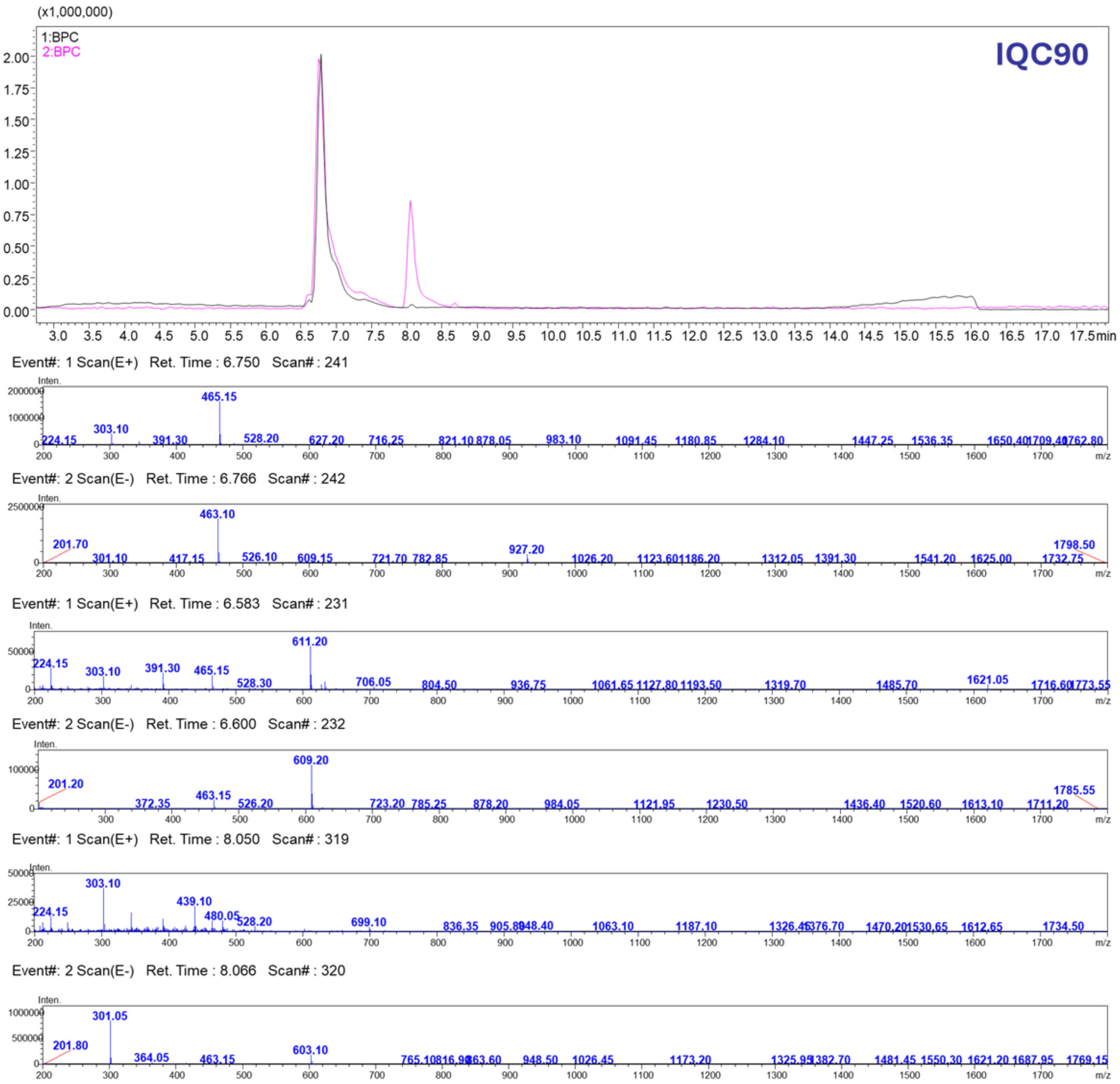

Figure S14. LC-MS chromatogram and MS spectra of IQC90. Base peak intensity chromatogram and low–low-resolution MS data in positive and negative mode of the peak at t_R= 6.75, 6.58, and 8.05 min. Column: YMC Pack ODS-AQ (C18) 120 Å, 3 µm (150 x 2.0 mm). Elution gradient: 5:95 ACN (0.1% FA)-H_2O (0.1% FA) to 100:0 ACN (0.1% FA) in 13.5 min, flow 0.3 mL/min.

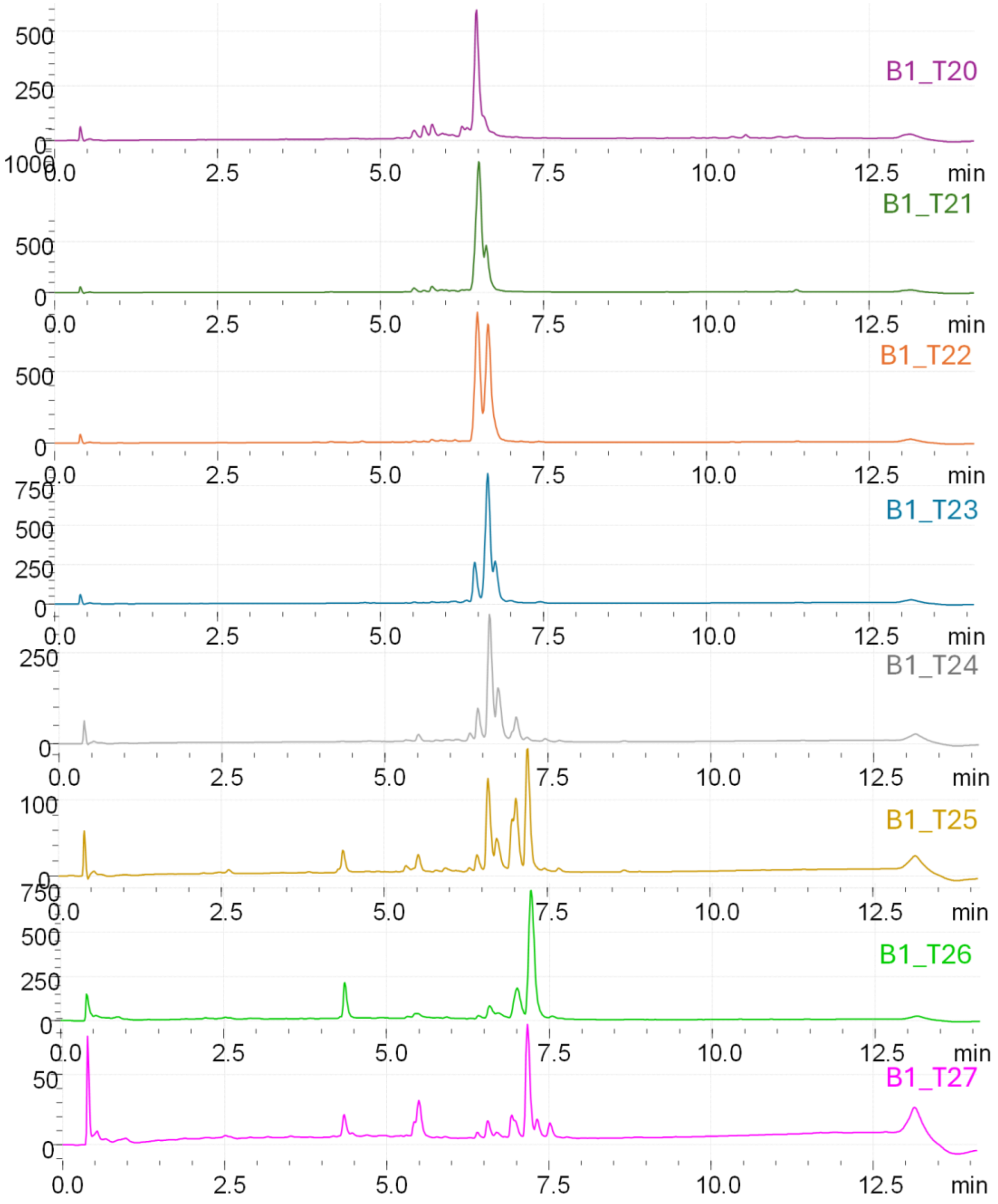

Figure S15. UHPLC analytical profiles of fractions T20 to T27. Chromatograms at 254 nm. Column: Acquity UPLC BEH C18 1.7 µm, 2.1 x 50 mm. Elution gradient: 10:90 ACN (0.1% FA) -H_2O (0.1% FA) to 100:0 ACN (0.1% FA) in 10 min, flow 0.5 mL/min.

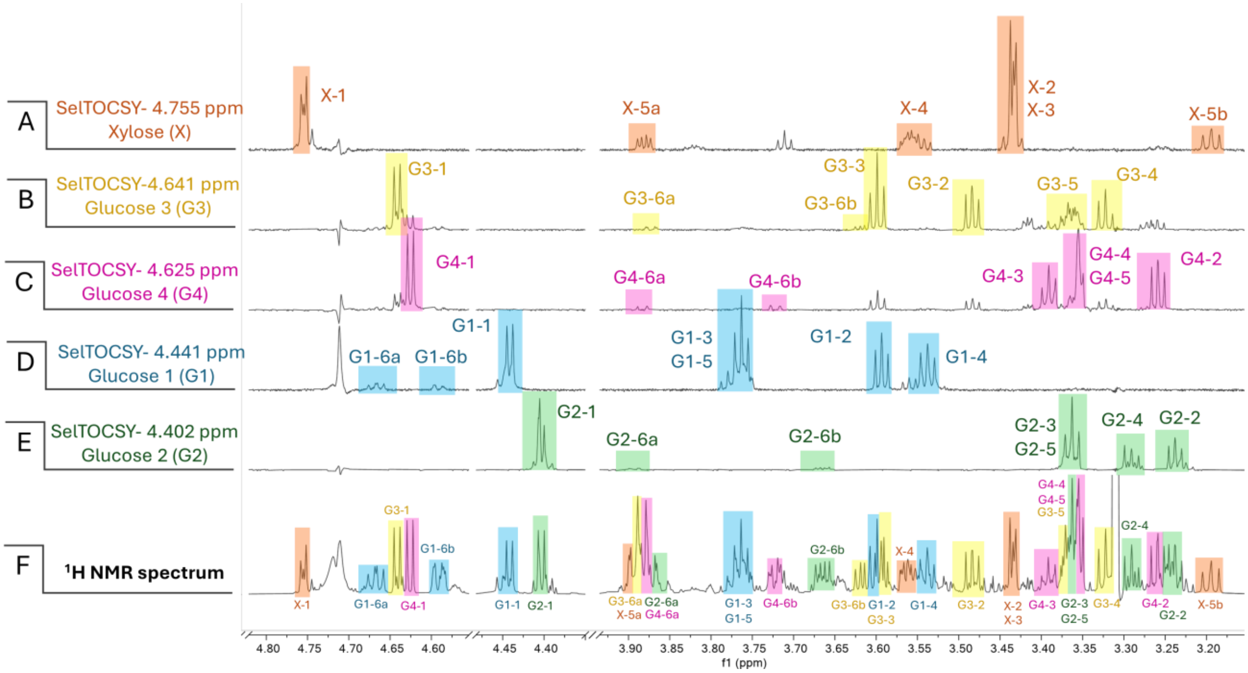

Figure S16. Stacked spectra of 1D selective TOCSY experiments (**A-E**; 90 ms mixing times) of each sugar residue that led to the assignment of all proton resonances in the full ^{1}H NMR spectrum (**F**) of **2**. The excitation frequencies were tuned to the anomeric proton of each sugar. This allows observation of the isolated spin systems of each sugar residue, recognition of multiplicities and, ultimately, the deduction of the coupling constants that identified one xylose (**A**) and four glucoses (**B-E**) as constituting monomers.

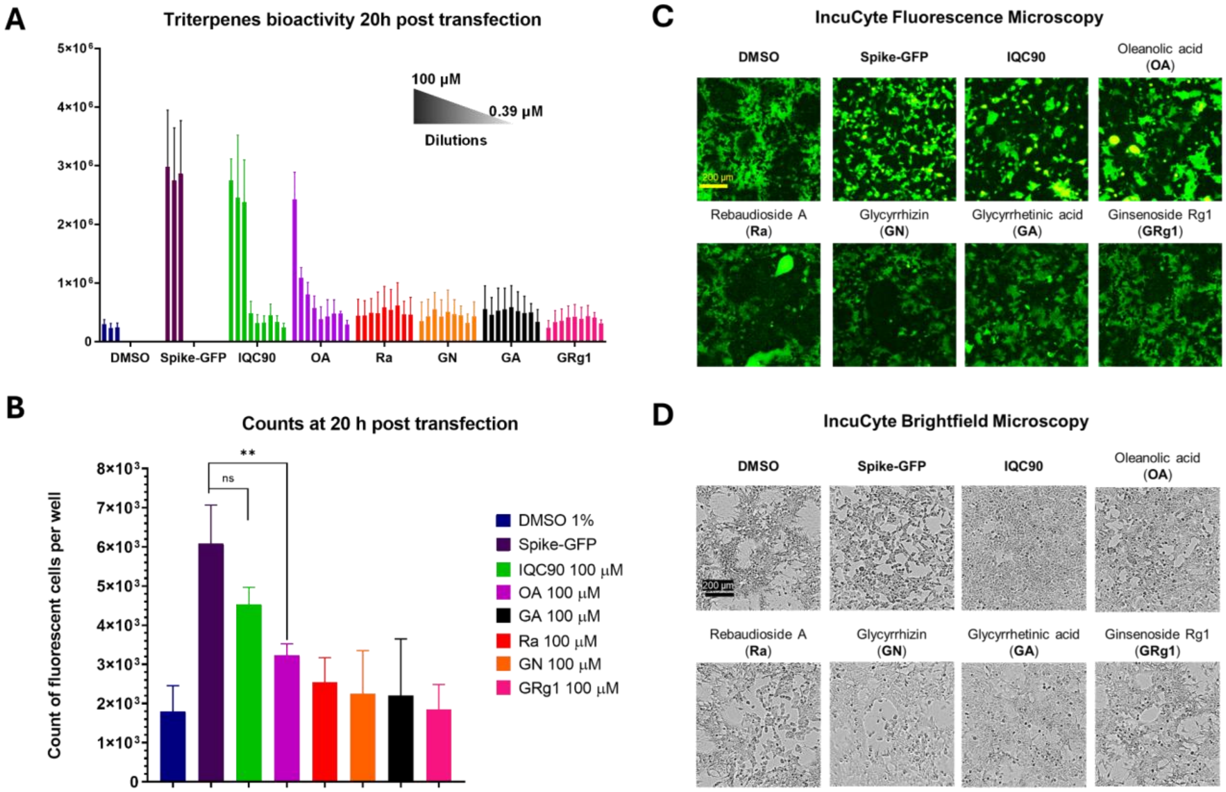

Figure S17. Evaluation of a panel of terpenoids in the HEKSA (see main text for rationalization of the selection of the terpenoids). **A.** Integrated fluorescence intensity reflecting the degree of syncytialization inhibition after 20 h for all the compounds in consecutive dilutions from 100 μM to 0.39 μM. The fluorescence is observed with HEK293 cells expressing Spike-GFP + hACE2 except for bar Spike-GFP only. Negative control: 1% DMSO (vehicle). **B.** Counts of isolated fluorescence events per well of all the compounds at 100 μM, measured at 20 h of incubation. **C.** Fluorescence microscopy images after 20 h of incubation, compounds tested at 100 μM. Brightness and contrast adjusted to 60%, the scale is 200 μm. Syncytialization is reflected by the presence of cell-less black plaques formed following cell fusion into syncytia and the overall reduction of fluorescence intensity. oleanolic acid and IQC90 treatment significantly reduce the plaques. **D.** Brightfield microscopy images after 20 h of incubation, compounds tested at 100 μM. The scale is 200 μm. Syncytialization is reflected by the presence of cell-less plaques and elongated cell morphology. IQC90-treated cells are well-rounded with virtually no plaques; those treated with oleanolic acid and glycyrrhetinic acid are rounding among smaller plaques, indicating incomplete inhibition.

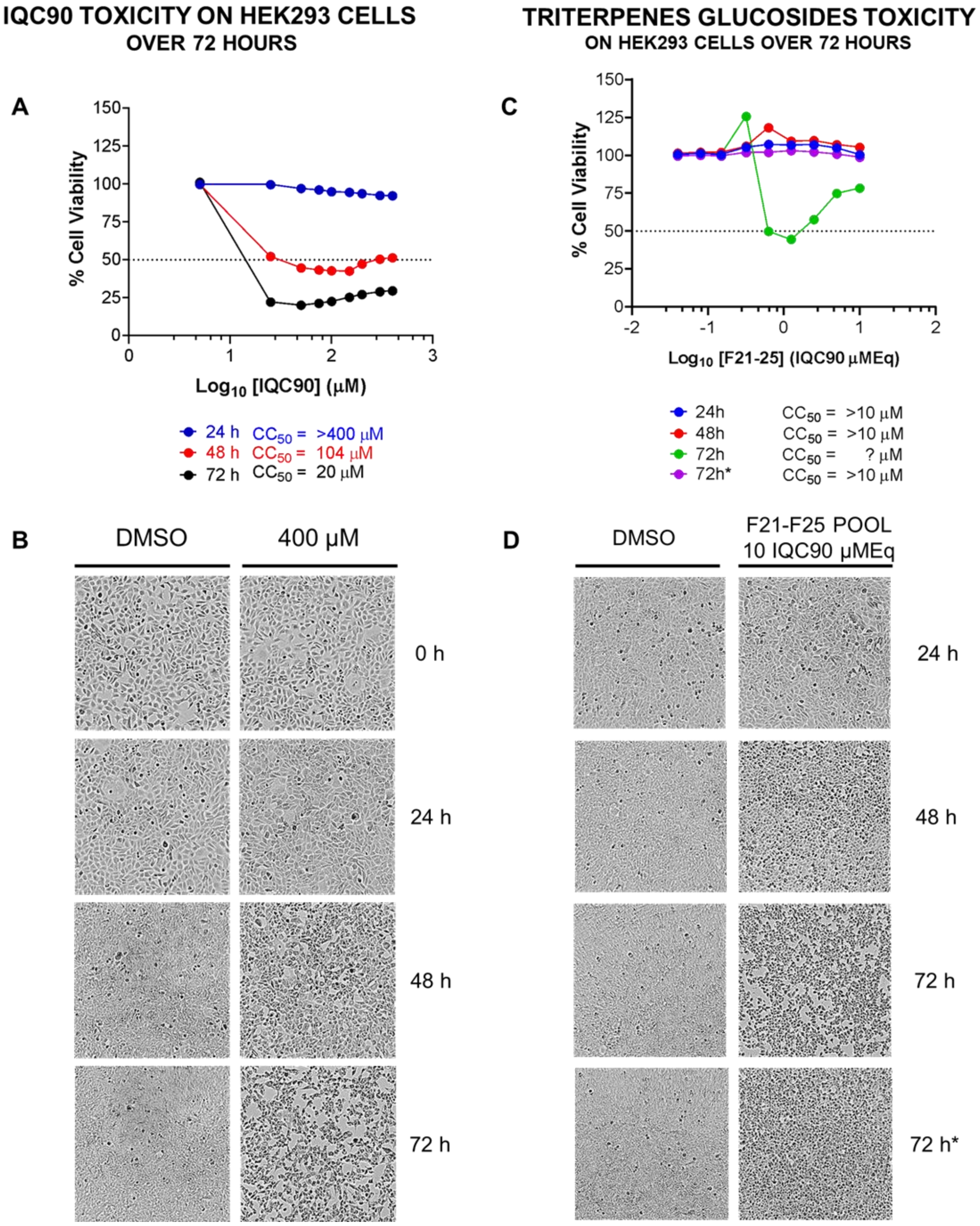

Figure S18. IQC90 and Triterpenes Glucosides Cytotoxicity on HEK293 Cells over 72 h. Cells were incubated in medium containing varying concentrations of either IQC90 (0-400 µM, panels A and B) of the F21-25 triterpene glucoside pool (0-10 IQC90 µMEq, panels C and D) for 24, 48, and 72 h. 72h* indicates changes of test medium every 24 h. B and D are brightfield microscopic images of cells treated with DMSO or the highest concentration of test compound.

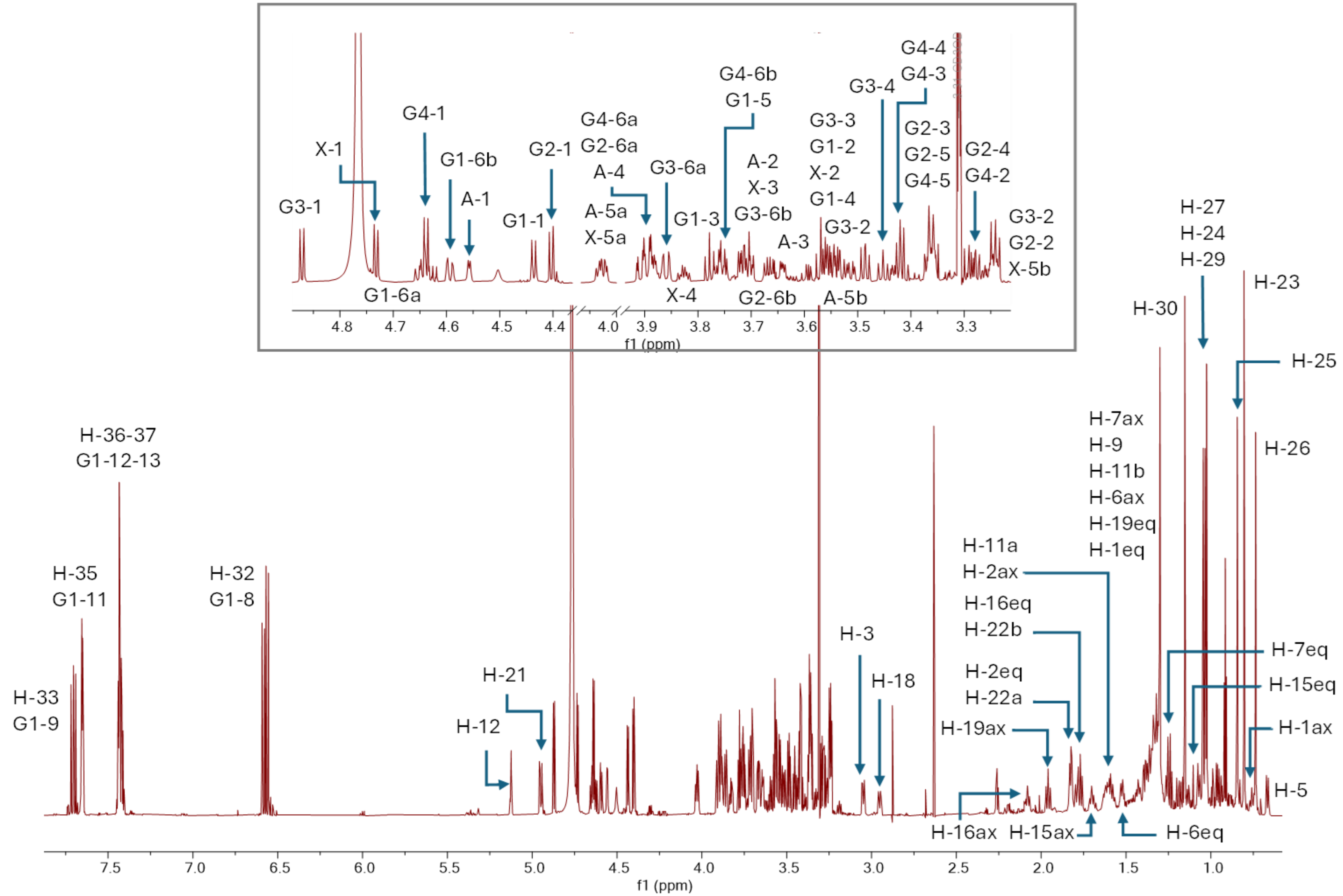

Figure S19. ^{1}H NMR spectrum of **1** at 1.1 GHz in MeOD/DMSO-d_6 (9:1).

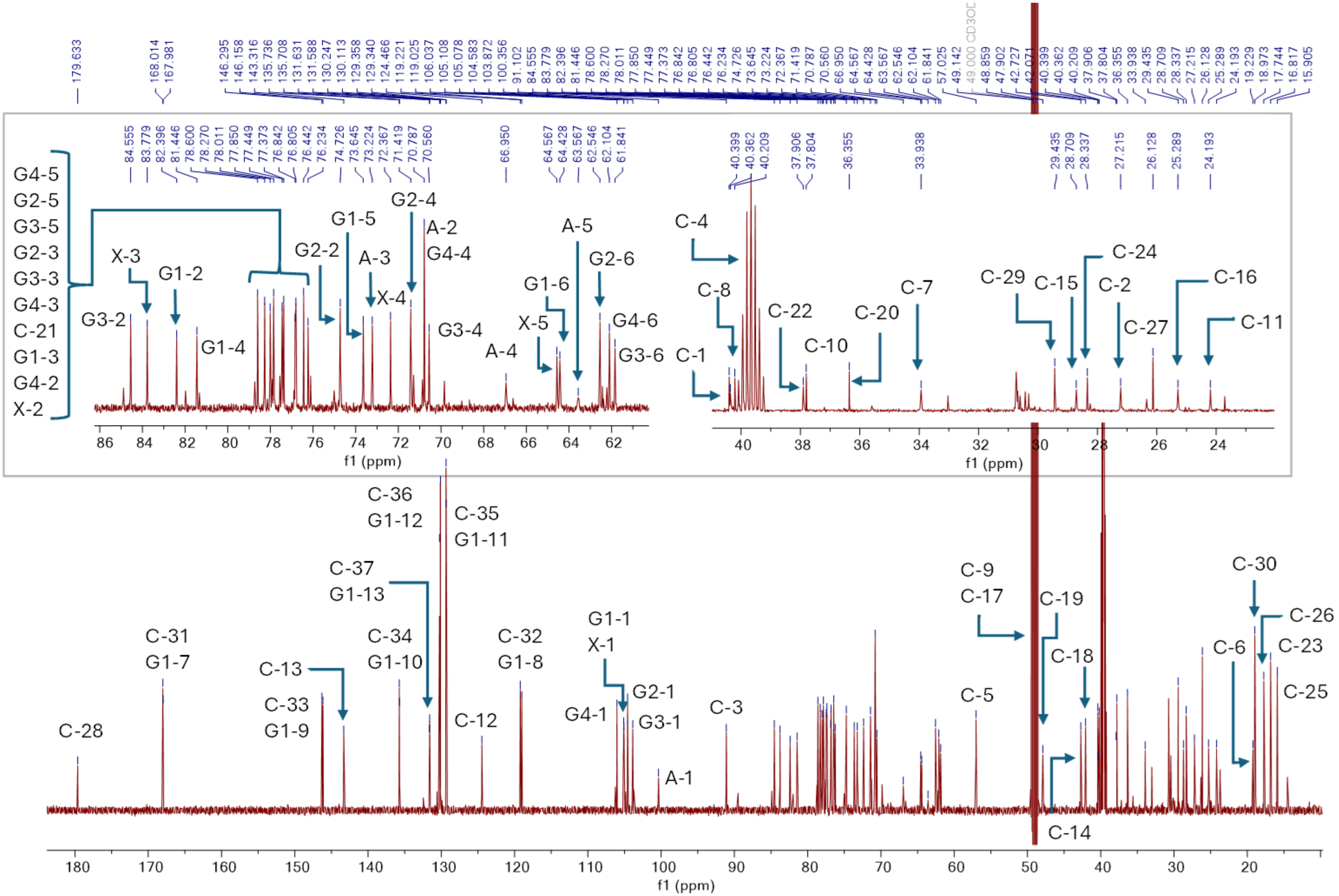

Figure S20. ^{13}C NMR spectrum of **1** at 150 MHz in MeOD/DMSO-d_6 (9:1).

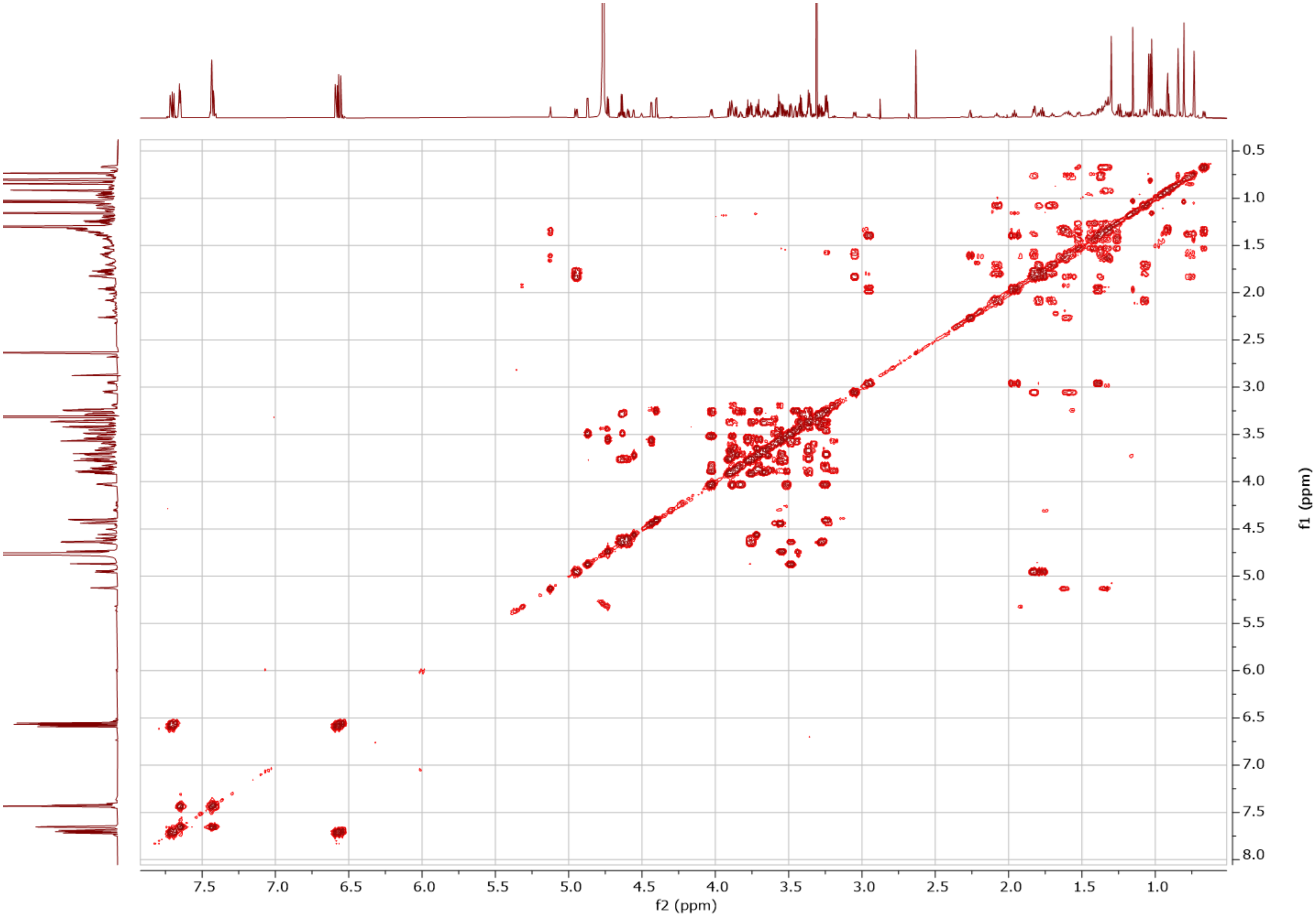

Figure S21. 2D COSY NMR spectrum of **1** at 600 MHz in MeOD/DMSO-d_6 (9:1).

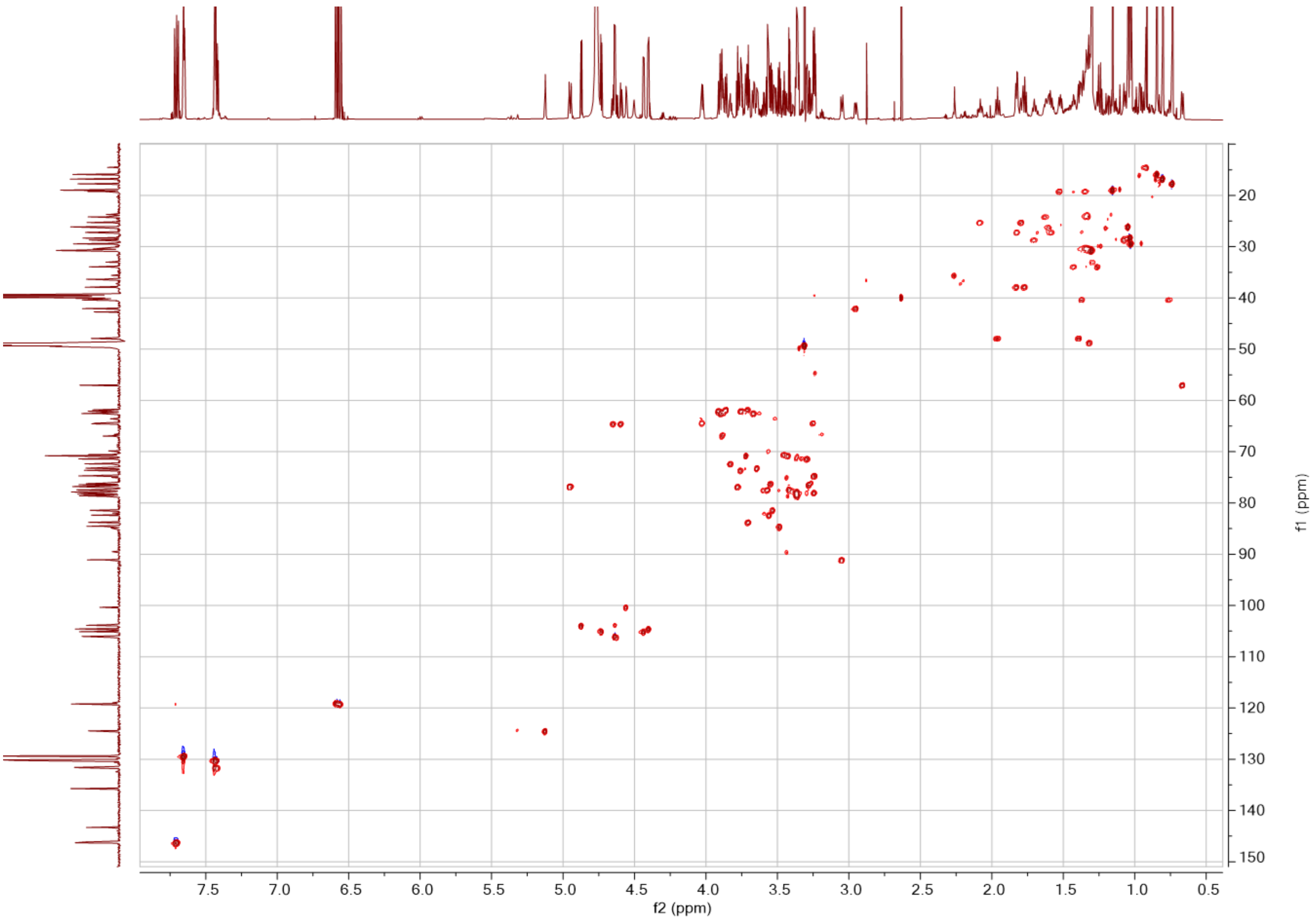

Figure S22. 2D HSQC NMR spectrum of **1** at 1.1 GHz in MeOD/DMSO-d_6 (9:1).

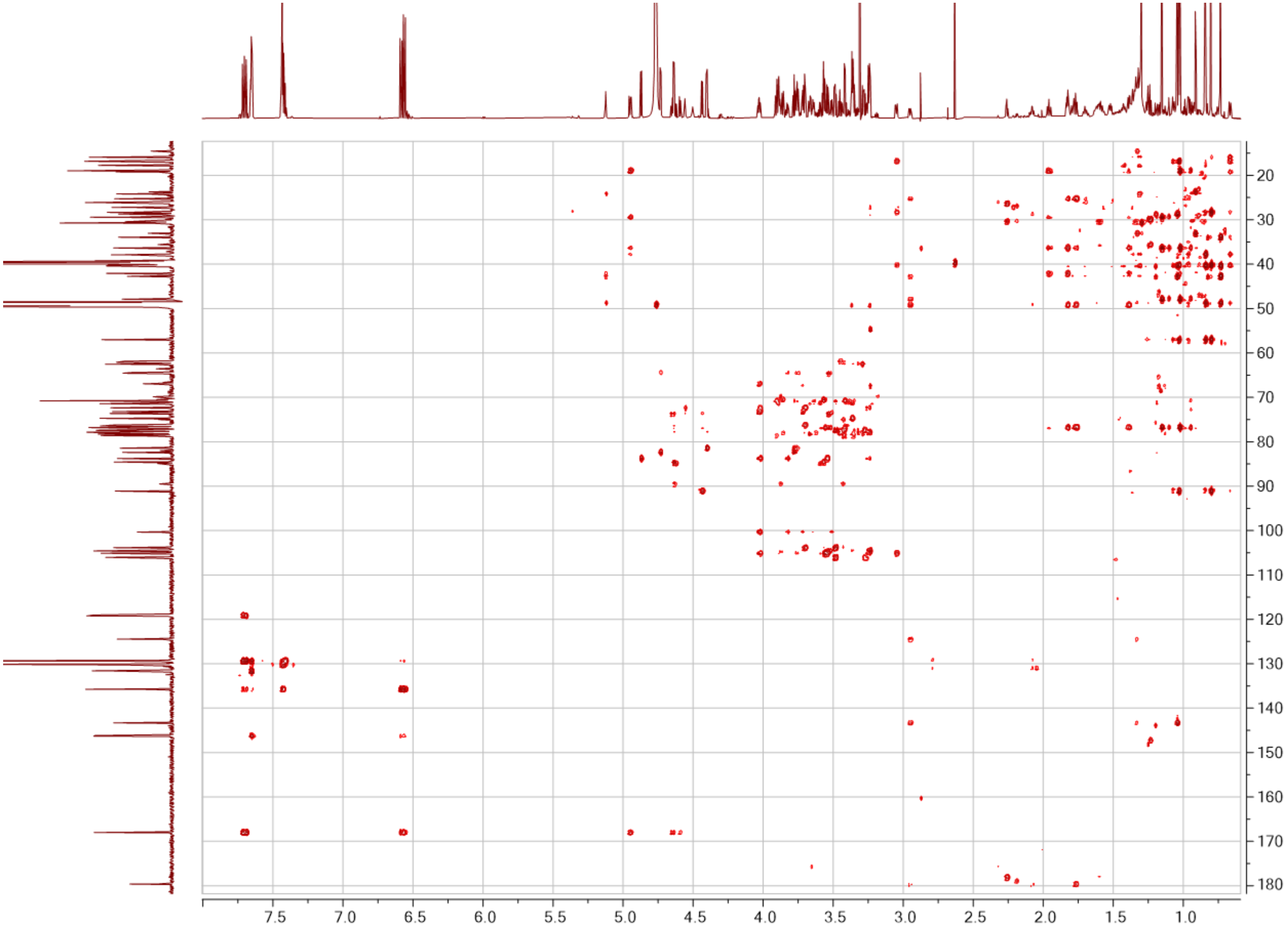

Figure S23. 2D HMBC NMR spectrum of **1** at 1.1 GHz in MeOD/DMSO-d_6 (9:1).

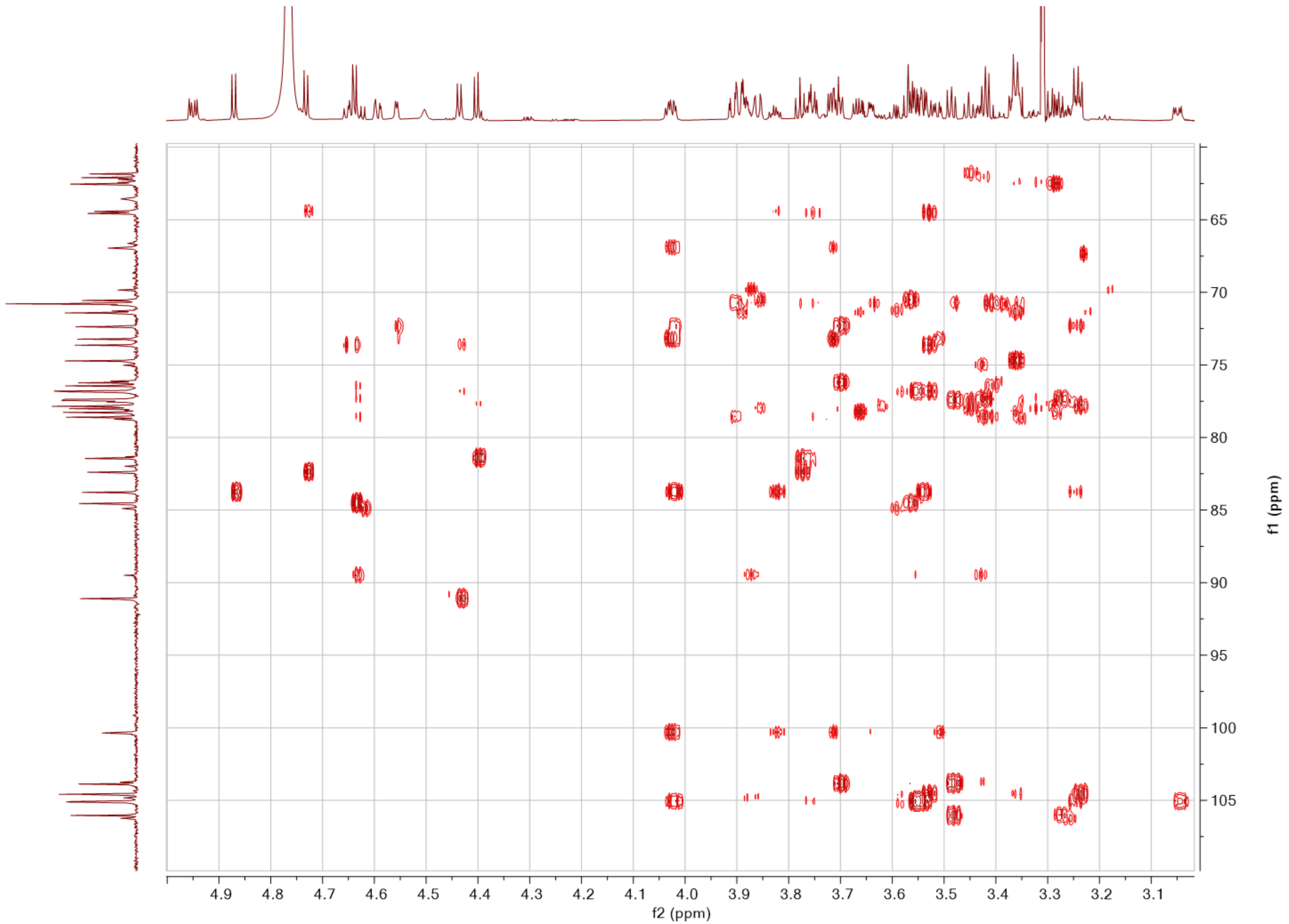

Figure S24. 2D HMBC NMR spectrum of **1** at 1.1 GHz in MeOD/DMSO-d_6 (9:1), expansion in the oligosaccharide area.

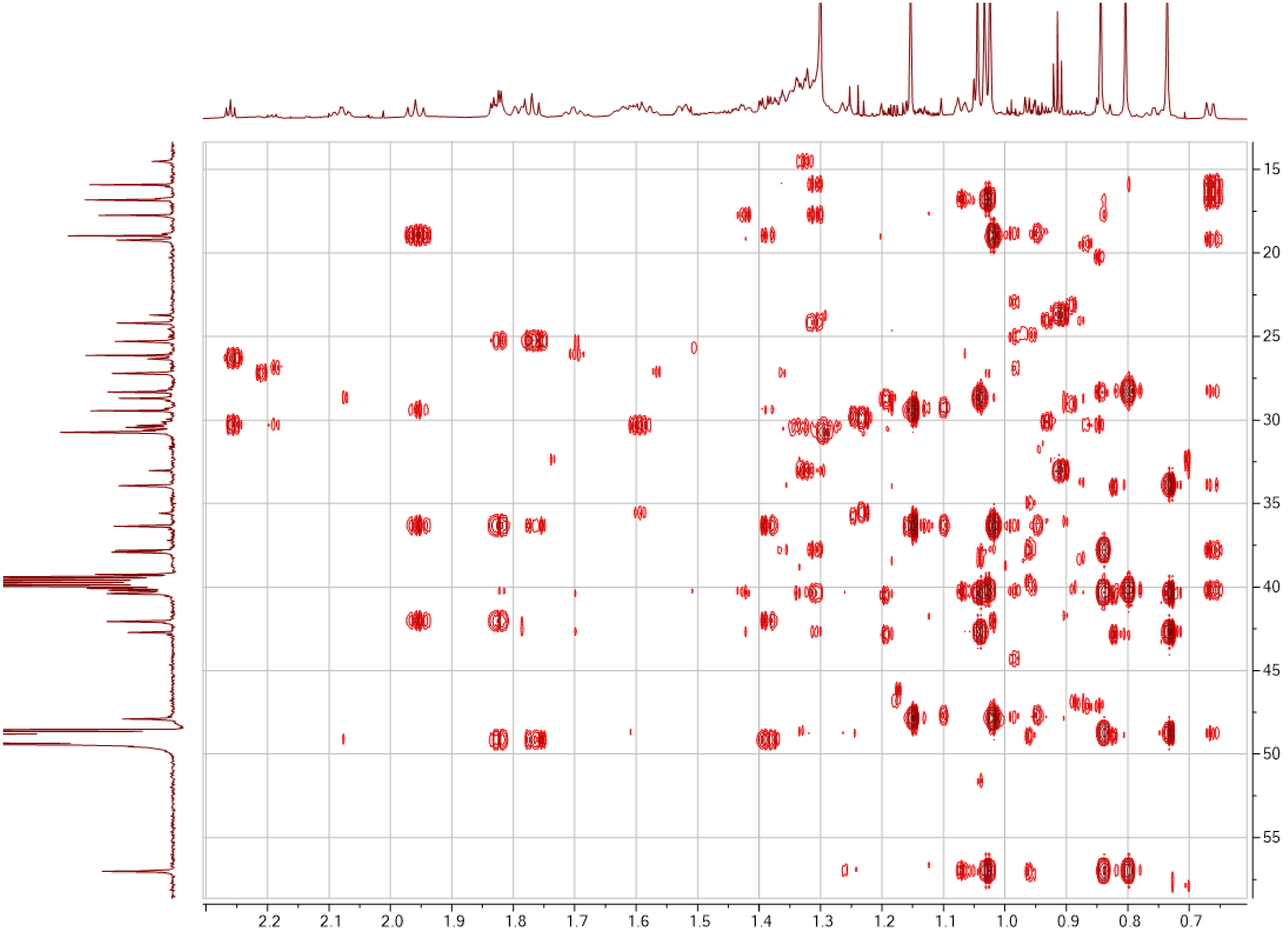

Figure S25. 2D HMBC NMR spectrum of **1** at 1.1 GHz in MeOD/DMSO-d_6 (9:1), expansion in the triterpene area.

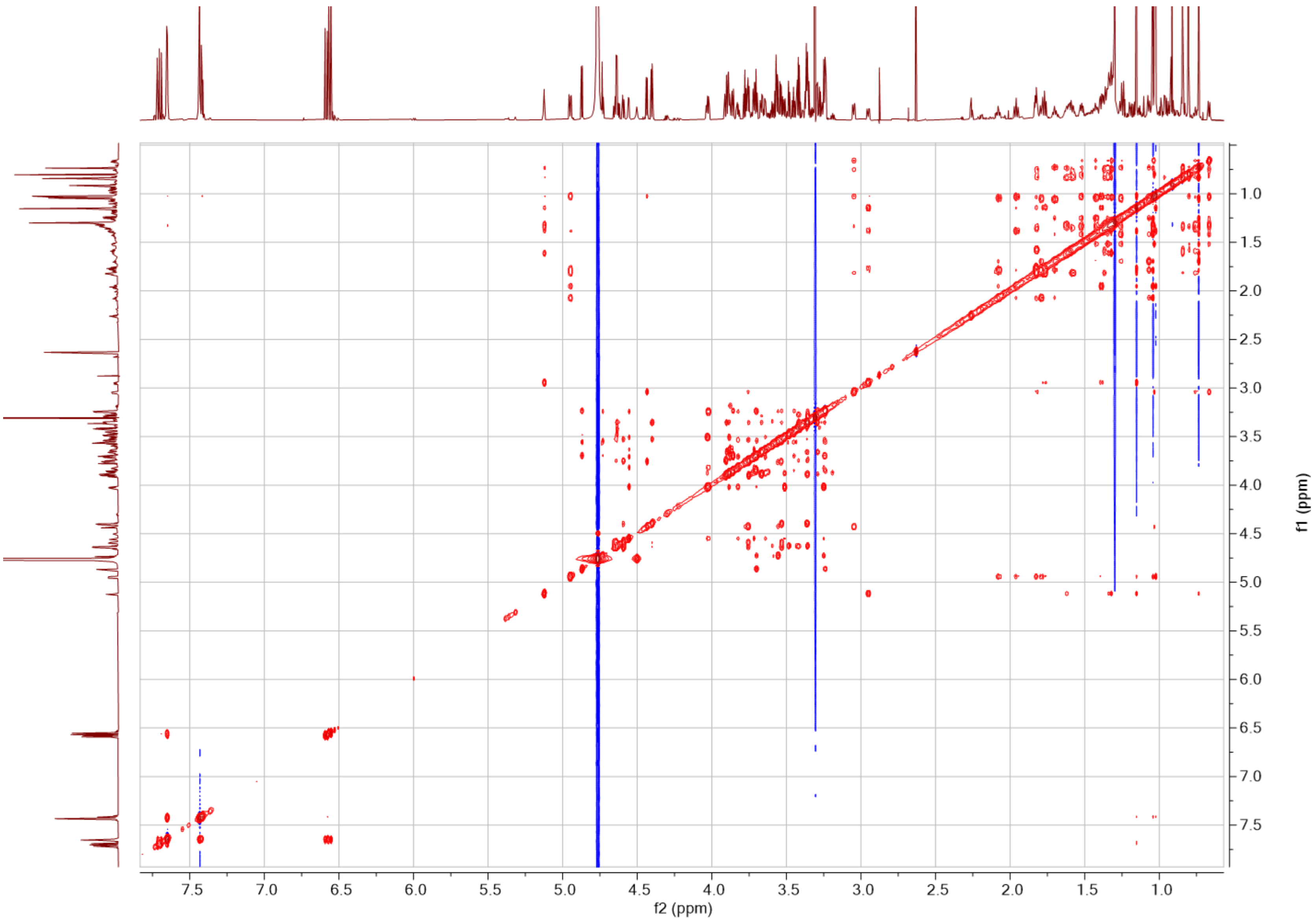

Figure S26. 2D NOESY NMR spectrum of **1** at 1.1 GHz in MeOD/DMSO-d_6 (9:1).

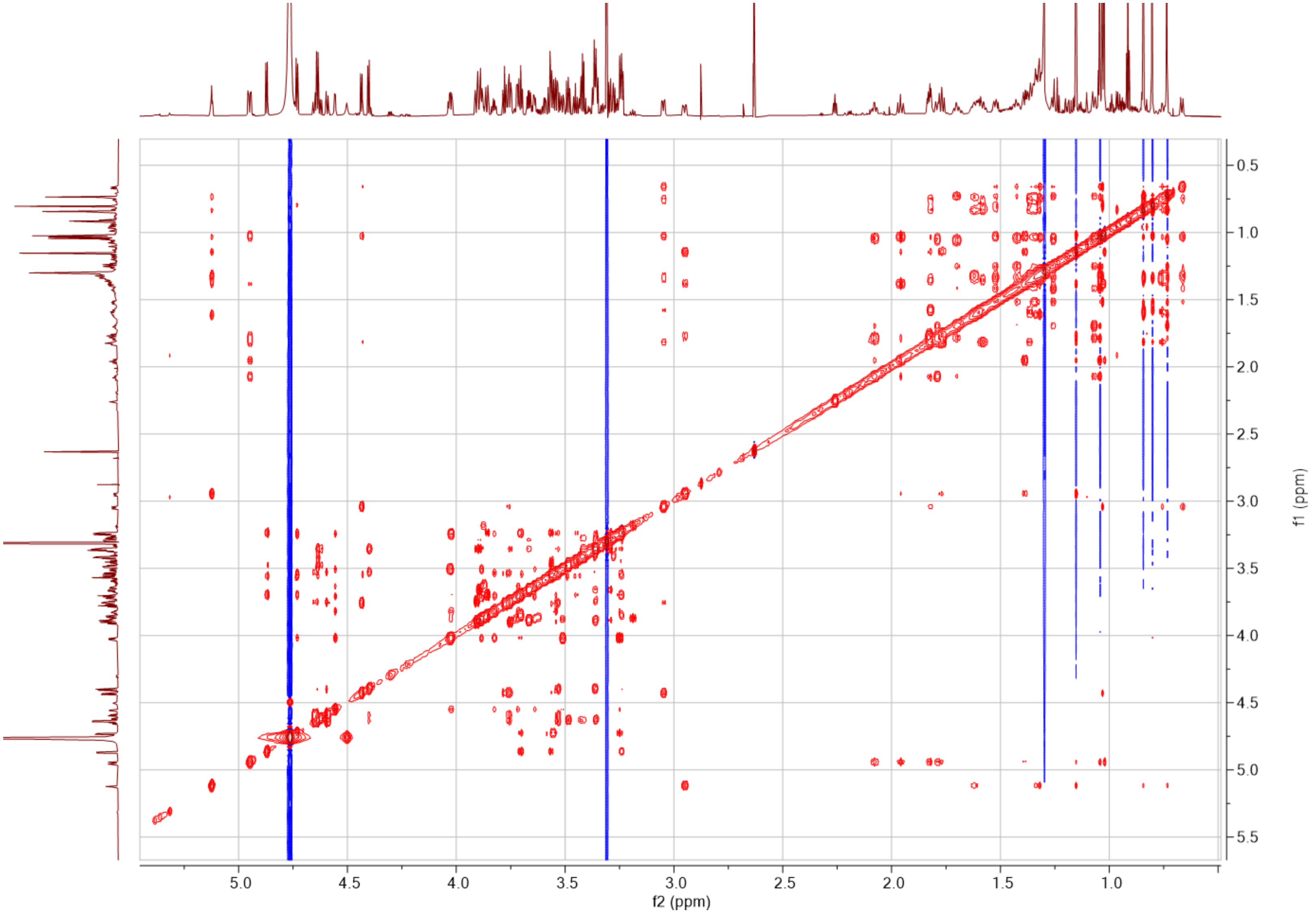

Figure S27. 2D NOESY NMR spectrum of **1** at 1.1 GHz in MeOD/DMSO-d_6 (9:1), expansion in the oligosaccharide and triterpene areas.

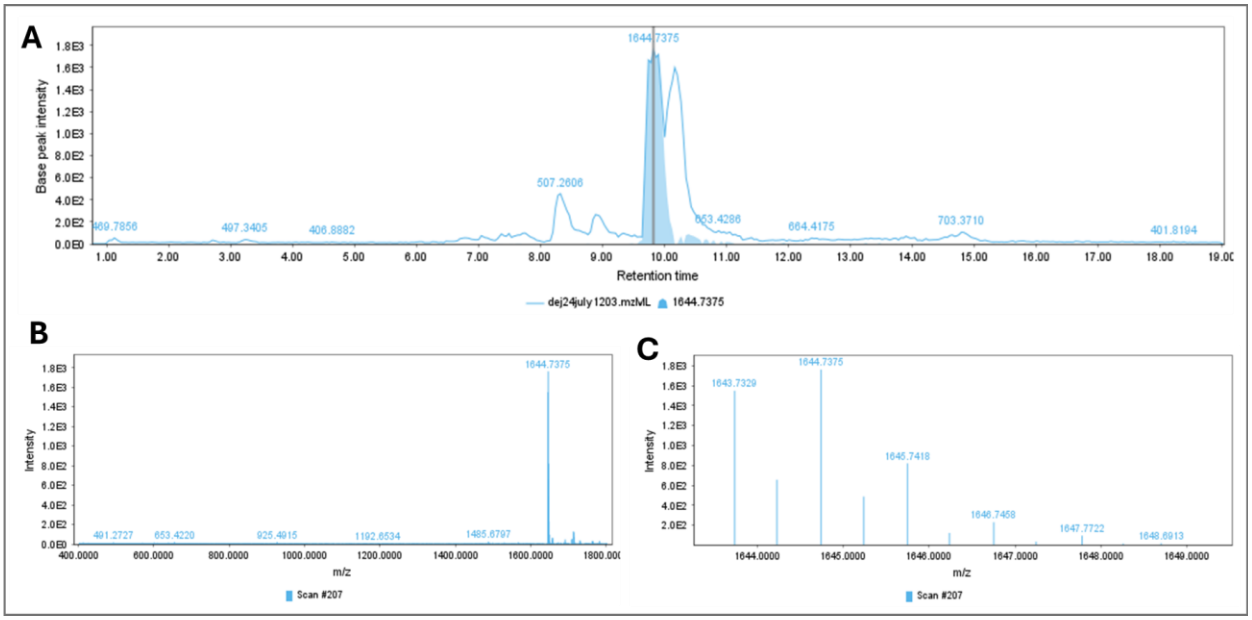

Figure S28. LC-MS chromatogram and MS spectra of fraction F21, containing compound **1** as the main compound. **A.** Base peak intensity chromatogram. Column: XBridge C18, 2.5 µm, 2.0 x 50 mm. Elution gradient: 15:85 ACN (0.1% FA)-H_2O (0.1% FA) to 100 ACN (0.1% FA) in 12 min, flow 0.2mL/min. **B.** High–resolution MS data in negative mode of the peak at t_R= 9.82 min, corresponding to compound **1**. **C.** Expansion of MS data in the mass range of interest.

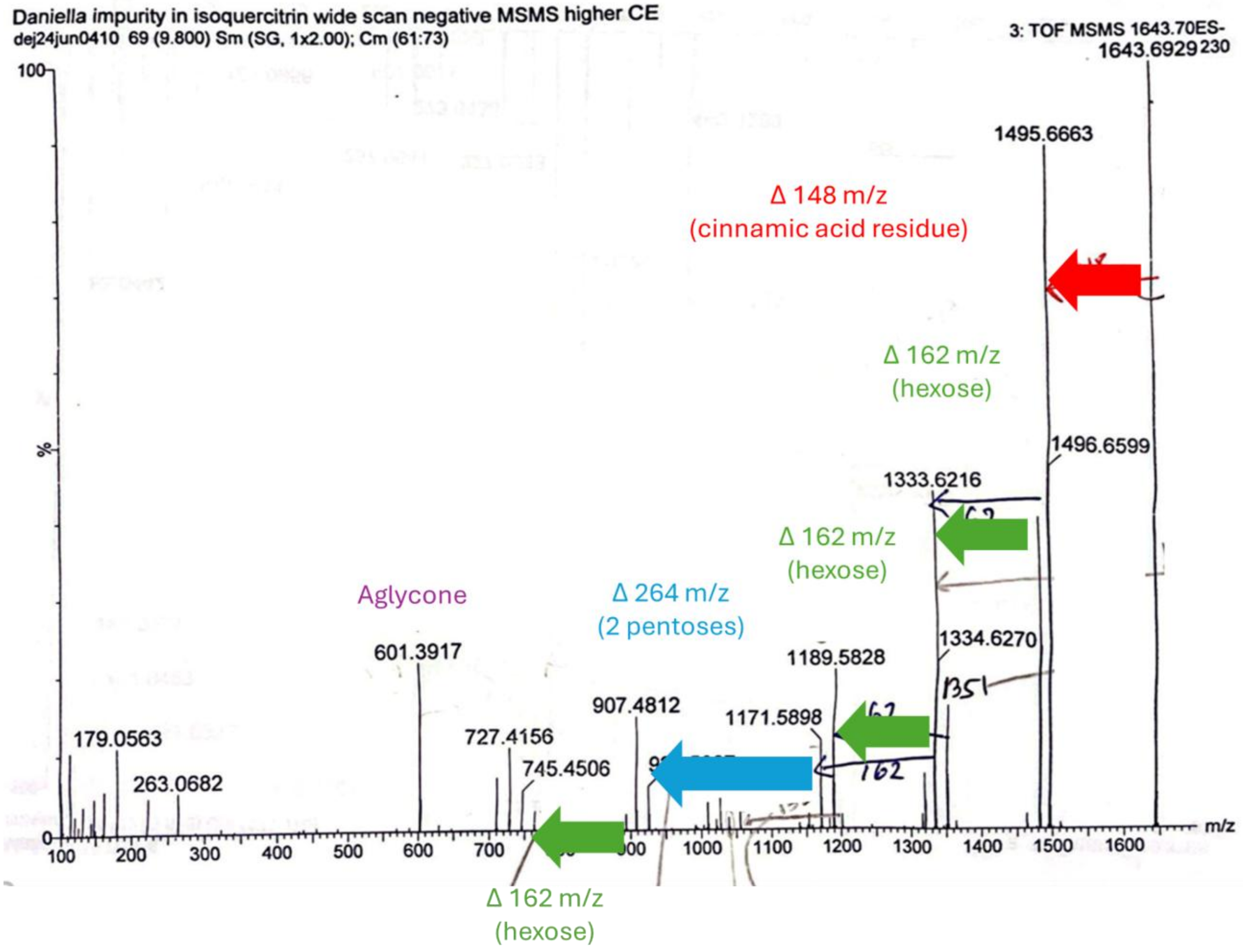

Figure S29. MS/MS fragmentation patterns for compound **1**.

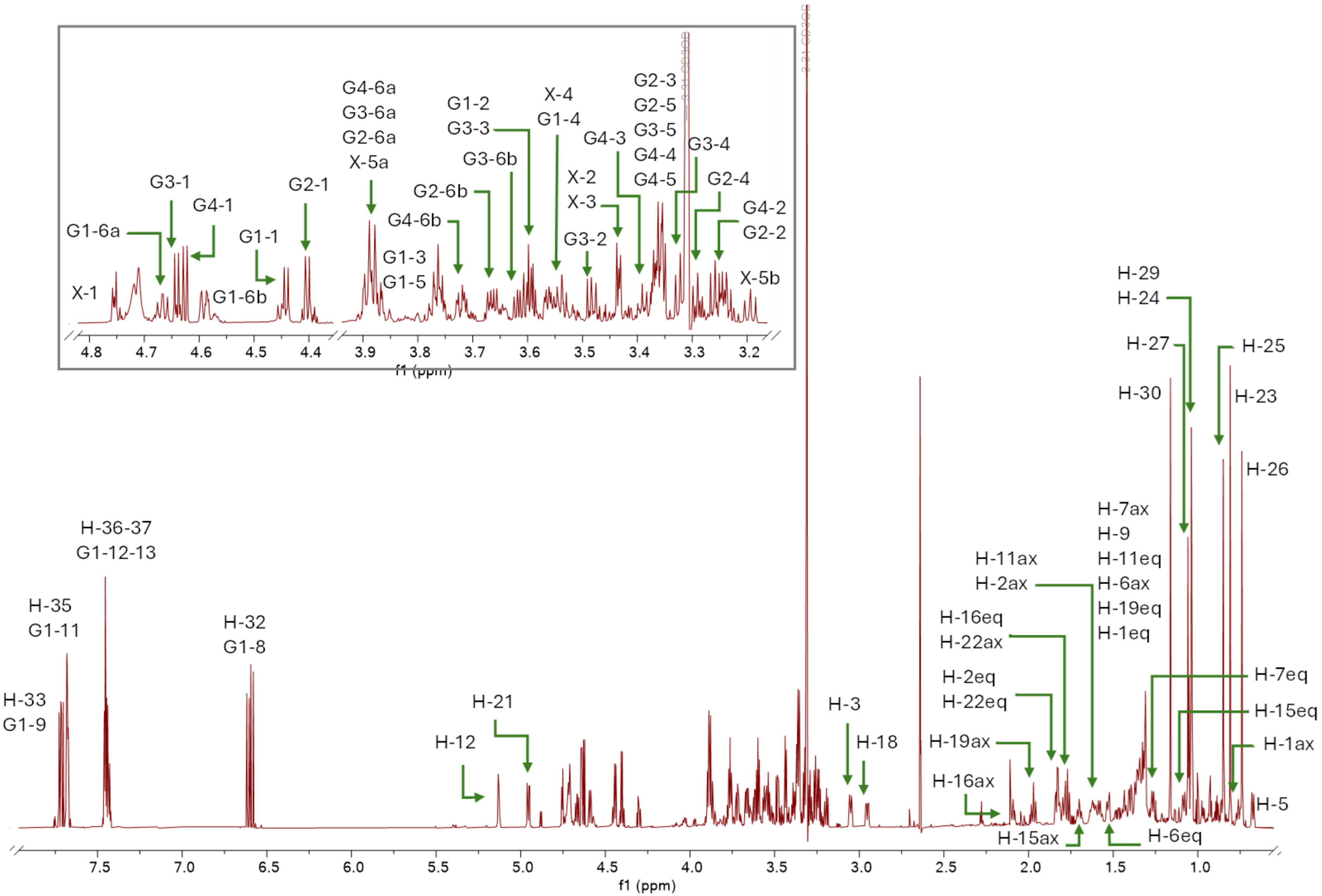

Figure S30. ^{1}H NMR spectrum of **2** at 1.1 GHz in MeOD/DMSO-d_6 (8:2).

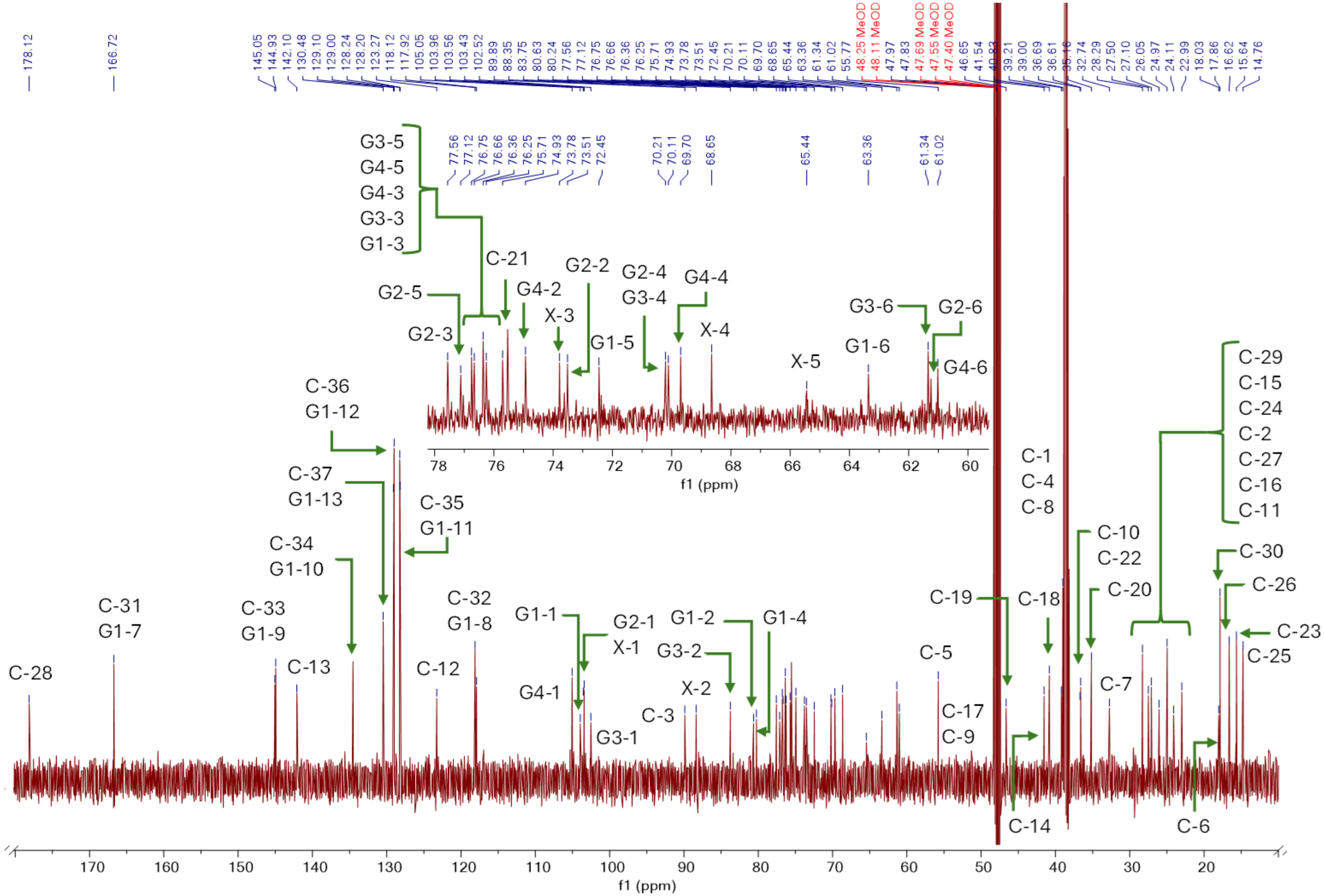

Figure S31. ^{13}C NMR spectrum of **2** at 150 MHz in MeOD/DMSO-d_6 (8:2).

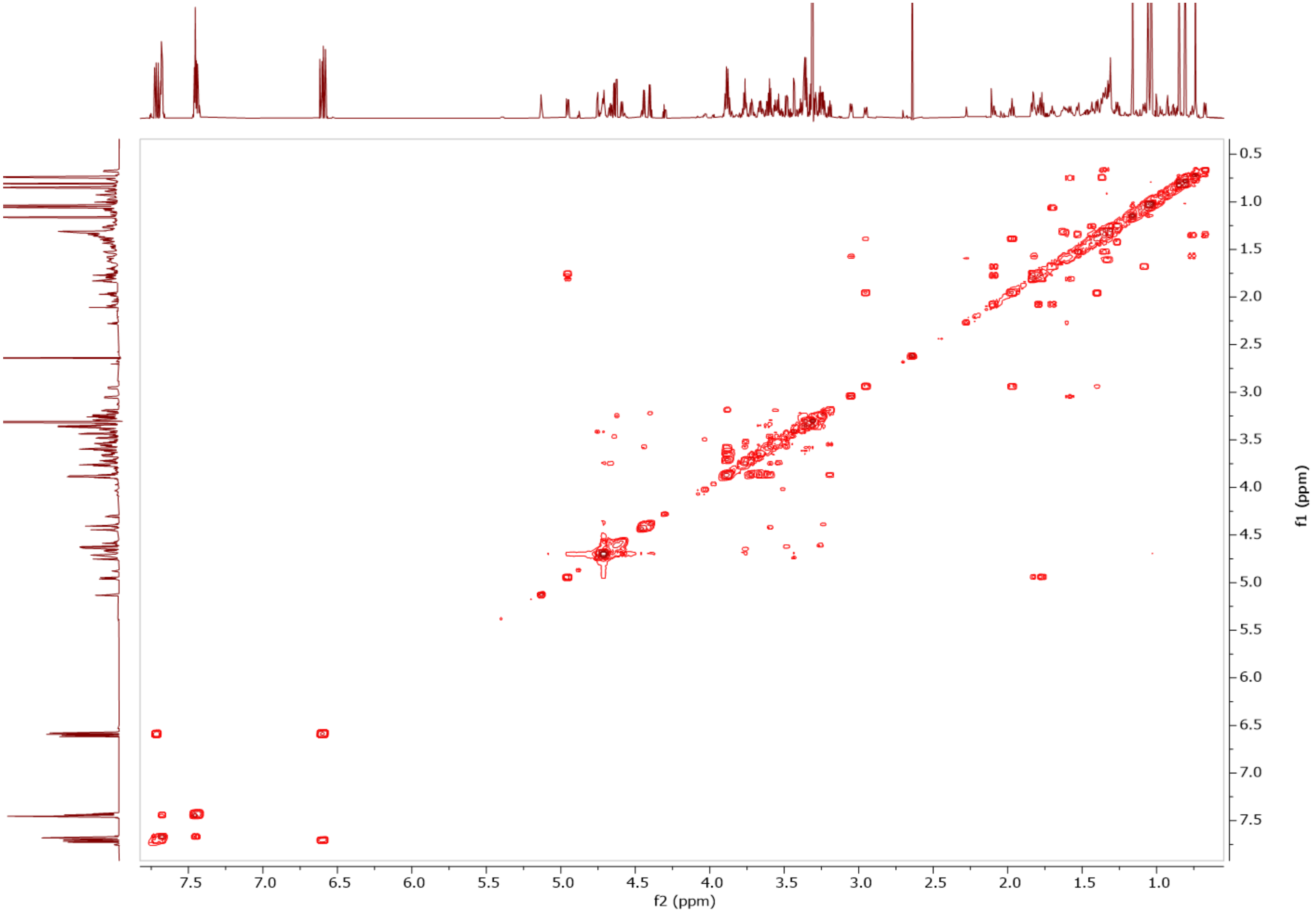

Figure S32. 2D COSY NMR spectrum of **2** at 1.1 GHz in MeOD/DMSO-d_6 (8:2).

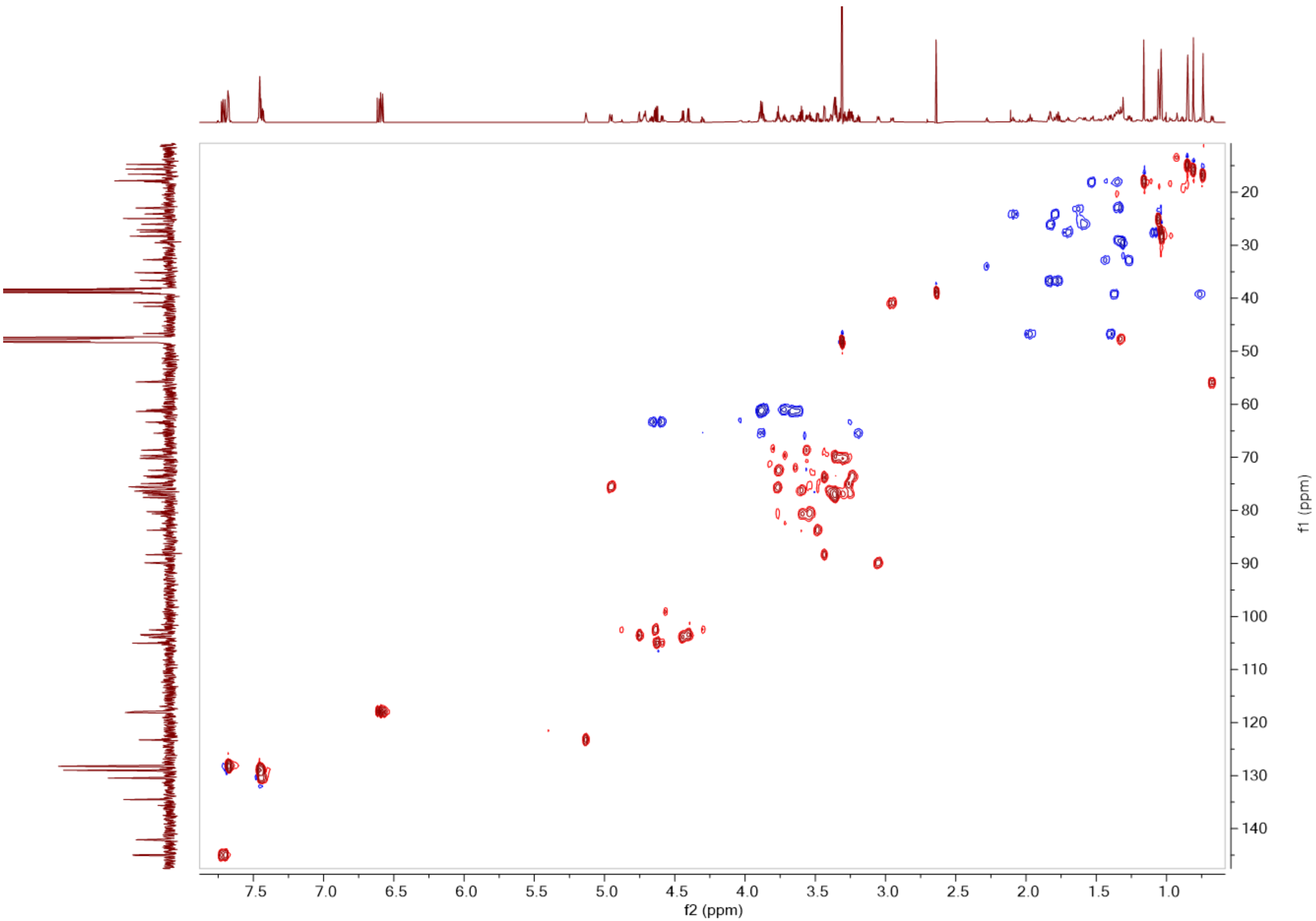

Figure S33. 2D HSQC NMR spectrum of **2** at 600 MHz in MeOD/DMSO-d_6 (8:2).

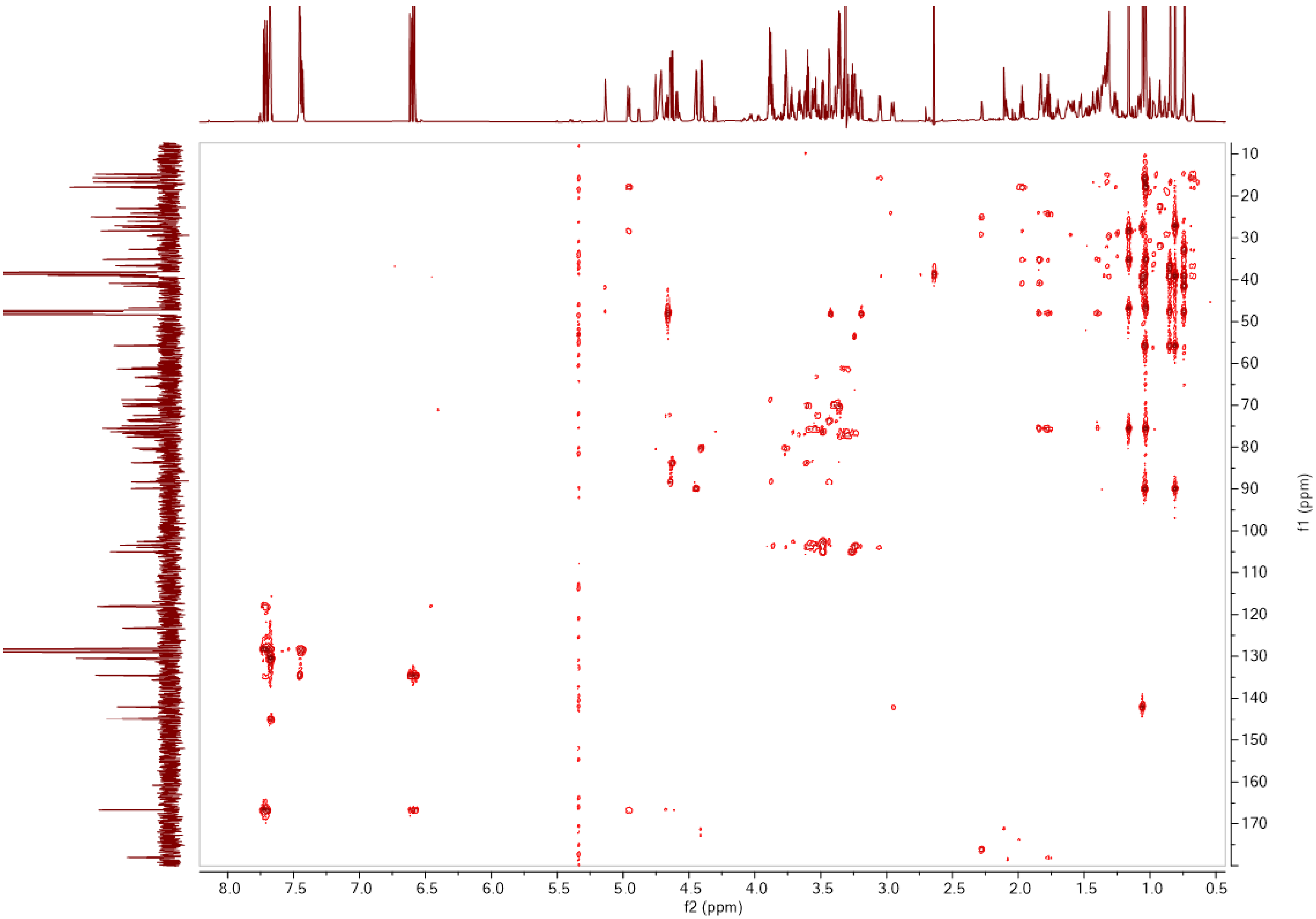

Figure S34. 2D HMBC NMR spectrum of **2** at 600 MHz in MeOD/DMSO-d_6 (8:2).

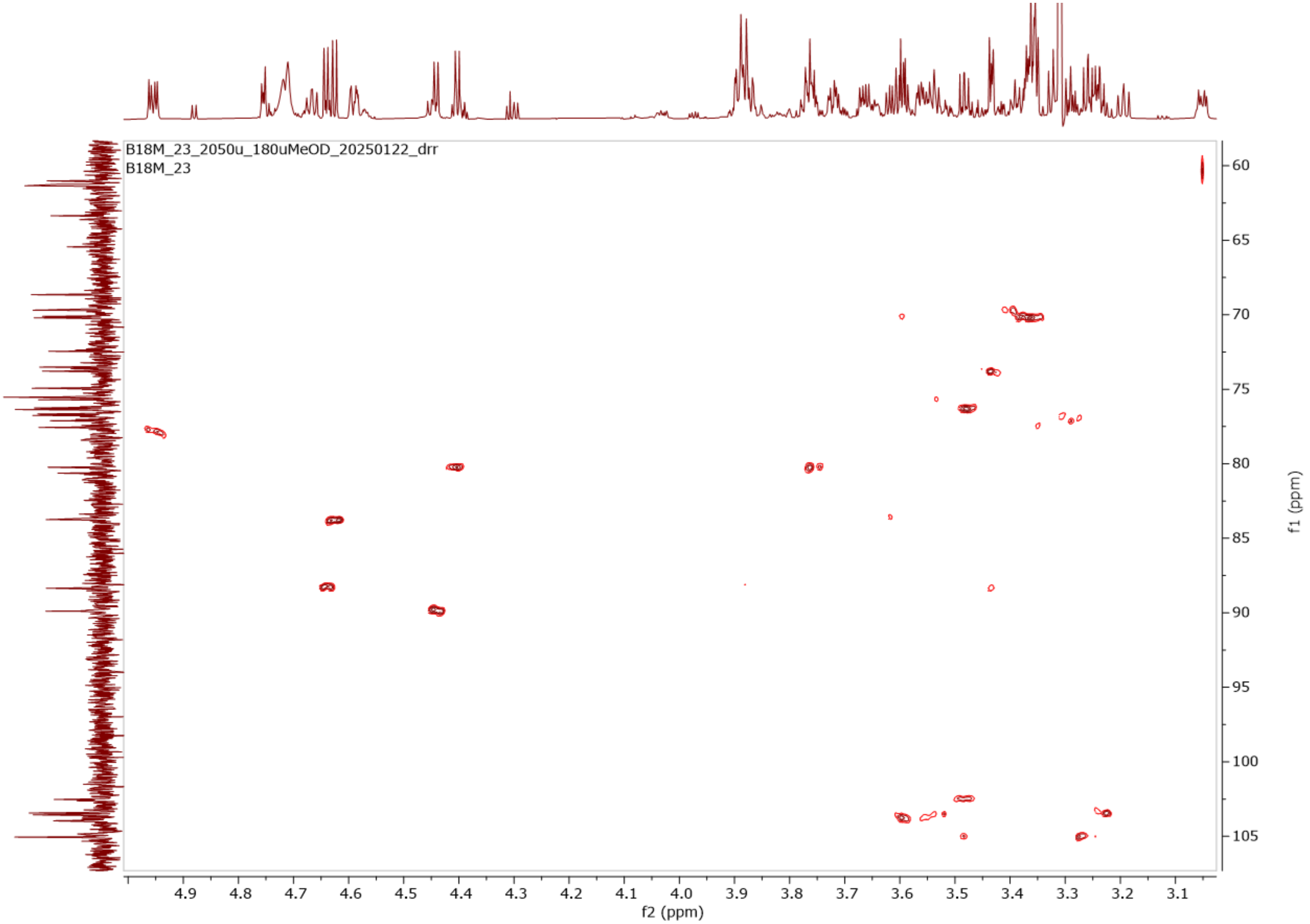

Figure S35. 2D HMBC NMR spectrum of **2** at 600 MHz in MeOD/DMSO-d_6 (8:2), expansion in the oligosaccharide area.

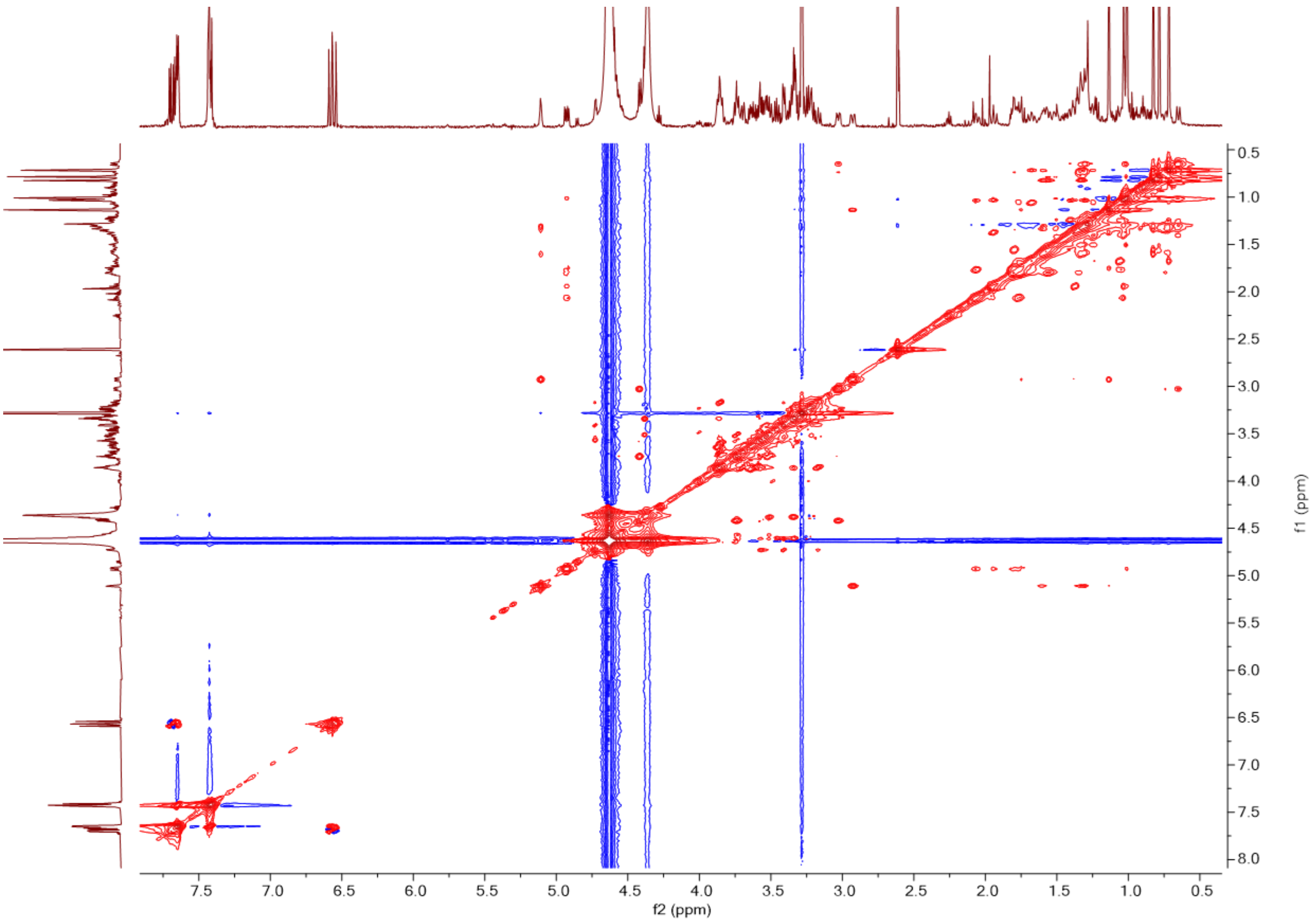

Figure S36. 2D NOESY NMR spectrum of **2** at 600 MHz in MeOD/DMSO-d_6 (8:2).

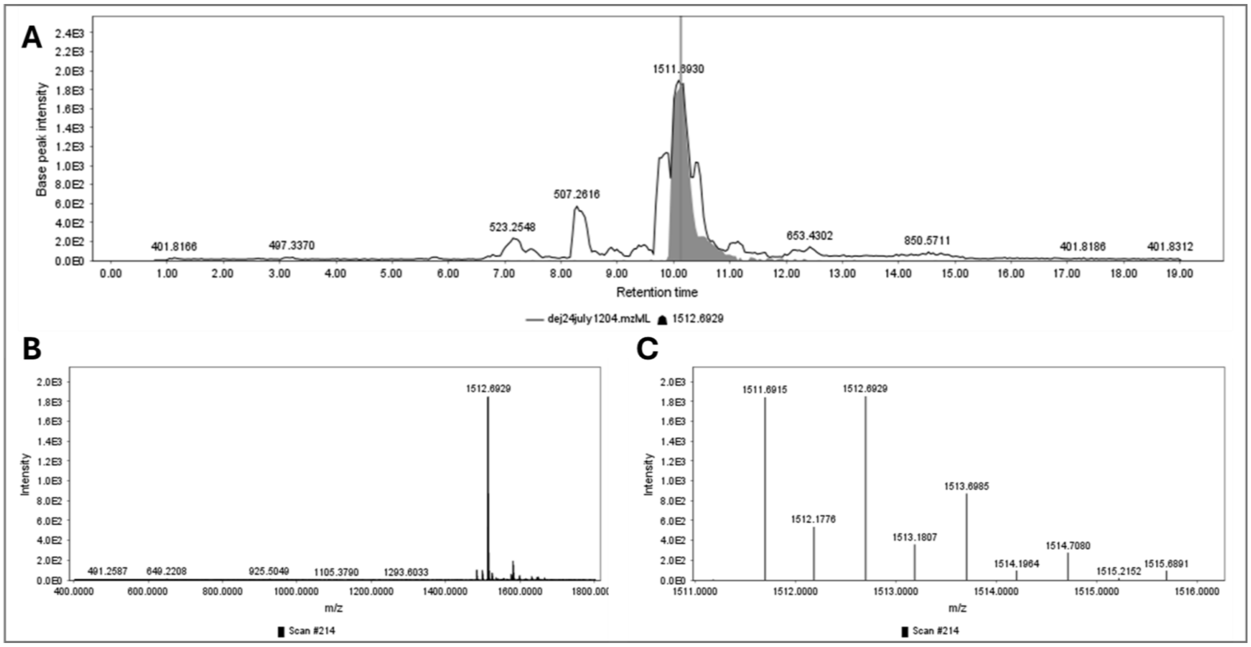

Figure S37. LC-MS chromatogram and MS spectra of fraction F23, containing compound **2** as the main compound. **A.** Base peak intensity chromatogram. Column: XBridge C18, 2.5 µm, 2.0 x 50 mm. Elution gradient: 15:85 ACN (0.1% FA)-H_2O (0.1% FA) to 100 ACN (0.1% FA) in 12 min, flow 0.2mL/min. **B.** High–resolution MS data in negative mode of the peak at t_R= 10.13 min, corresponding to compound **2**. **C.** Expansion of MS data in the mass range of interest.

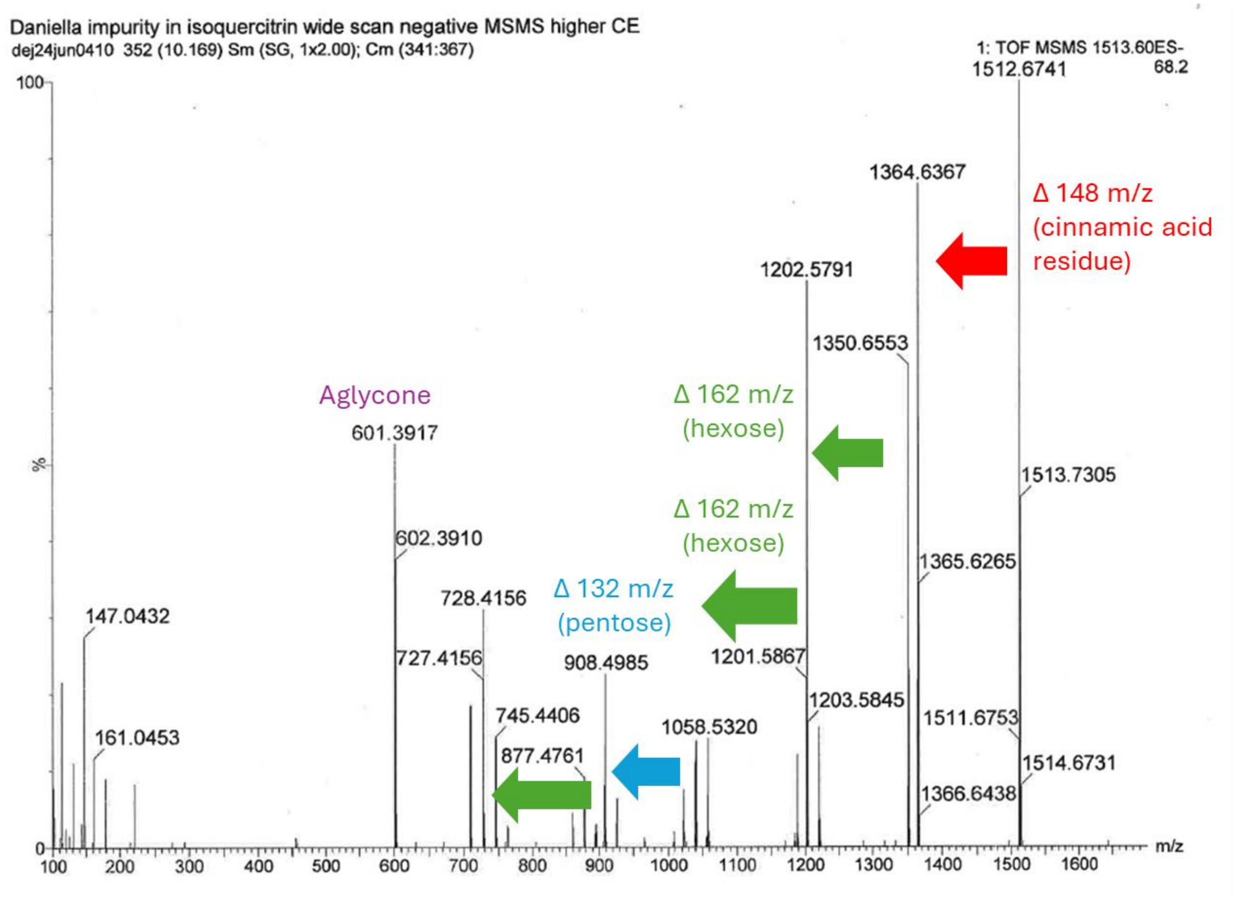

Figure S38. MS/MS fragmentation patterns for compound **2**.

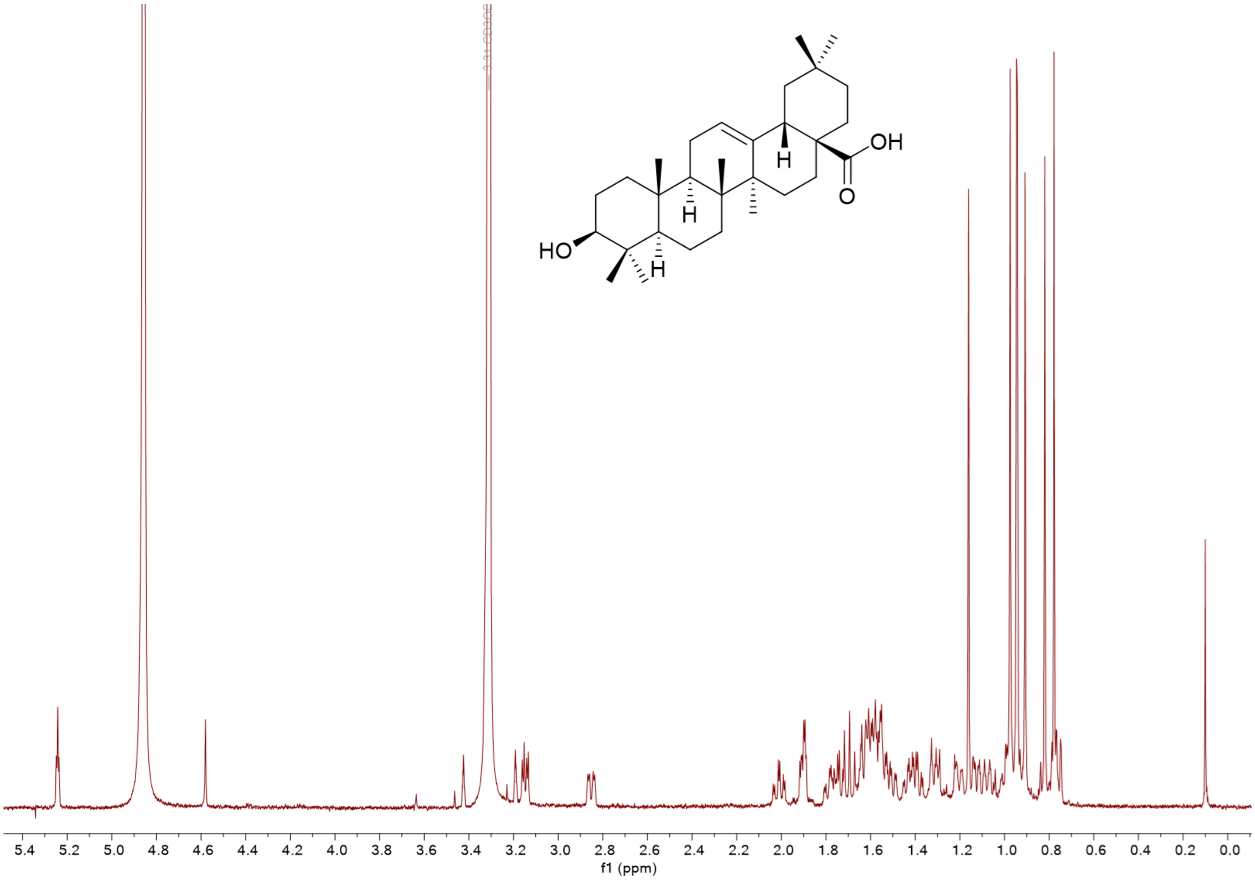

Figure S39. ^{1}H NMR spectrum of oleanolic acid at 600 MHz in MeOD.

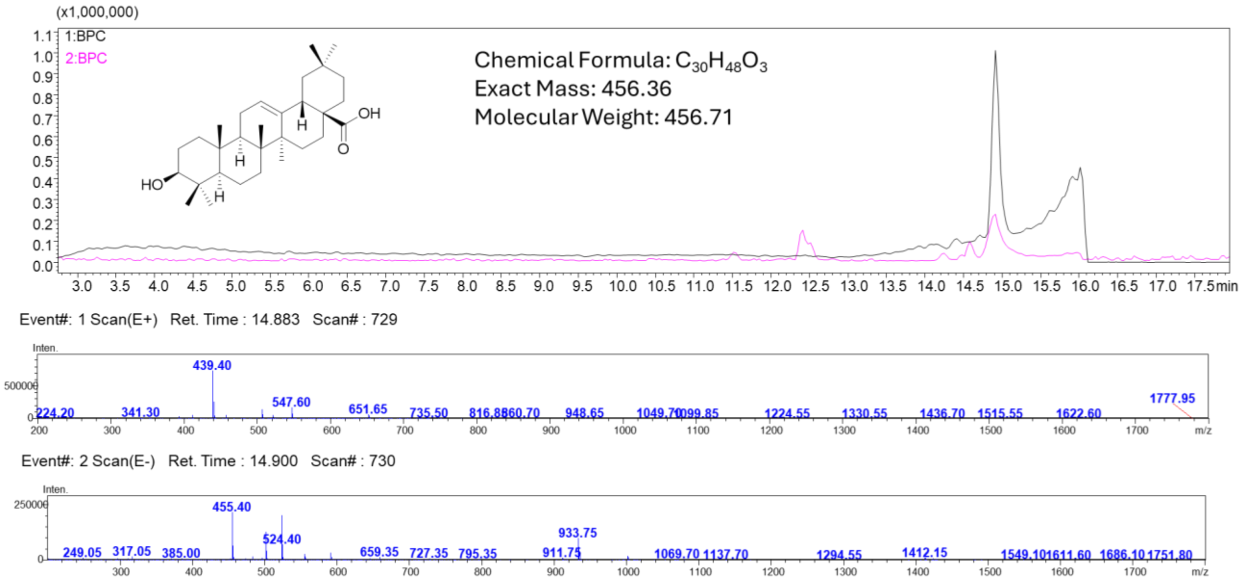

Figure S40. LC-MS chromatogram and MS spectra of oleanolic acid. Base peak intensity chromatogram and low–low-resolution MS data in positive and negative mode of the peak at t_R= 14.9 min. Column: YMC Pack ODS-AQ (C18) 120 Å, 3 µm (150 x 2.0 mm). Elution gradient: 5:95 ACN (0.1% FA)-H_2O (0.1% FA) to 100:0 ACN (0.1% FA) in 13.5 min, flow 0.3 mL/min.

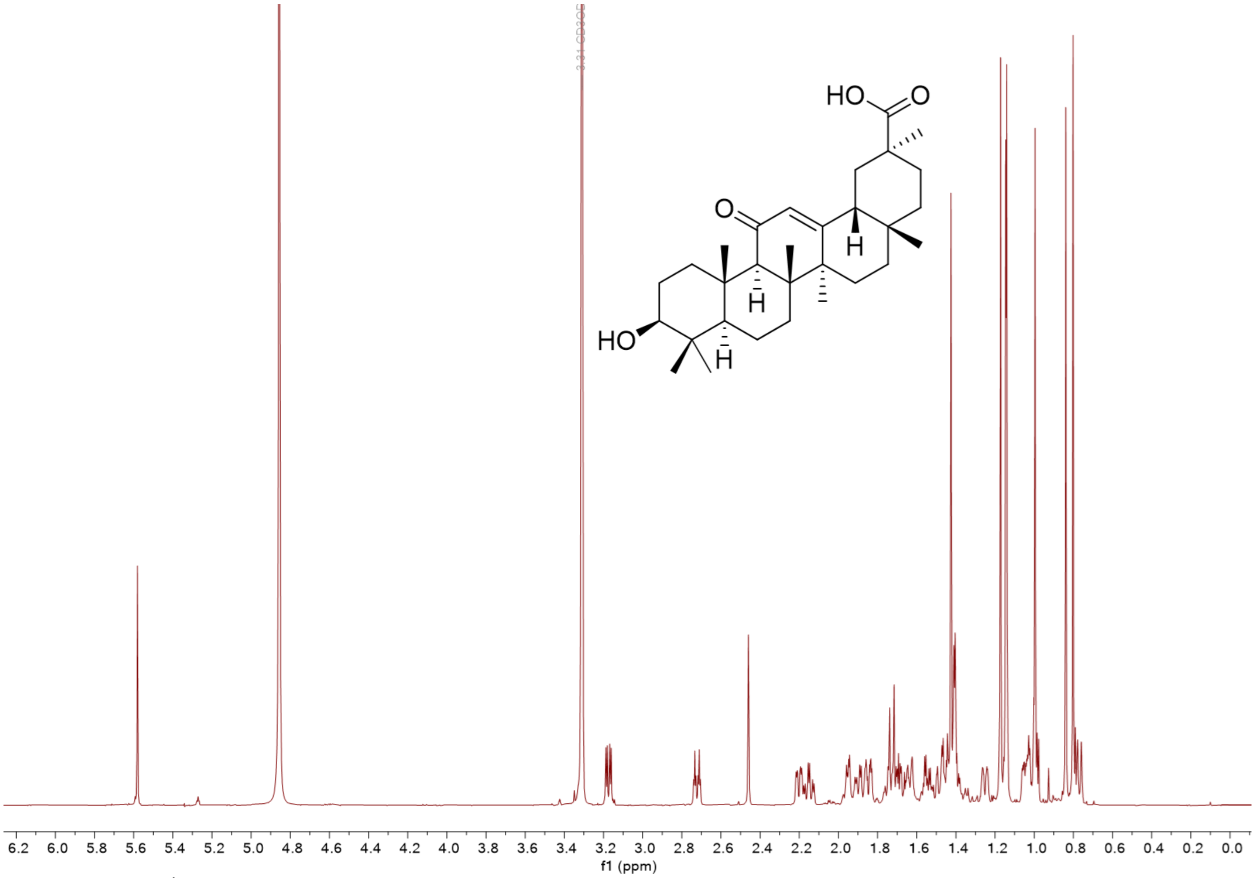

Figure S41. ^{1}H NMR spectrum of glycyrrhetinic acid at 600 MHz in MeOD.

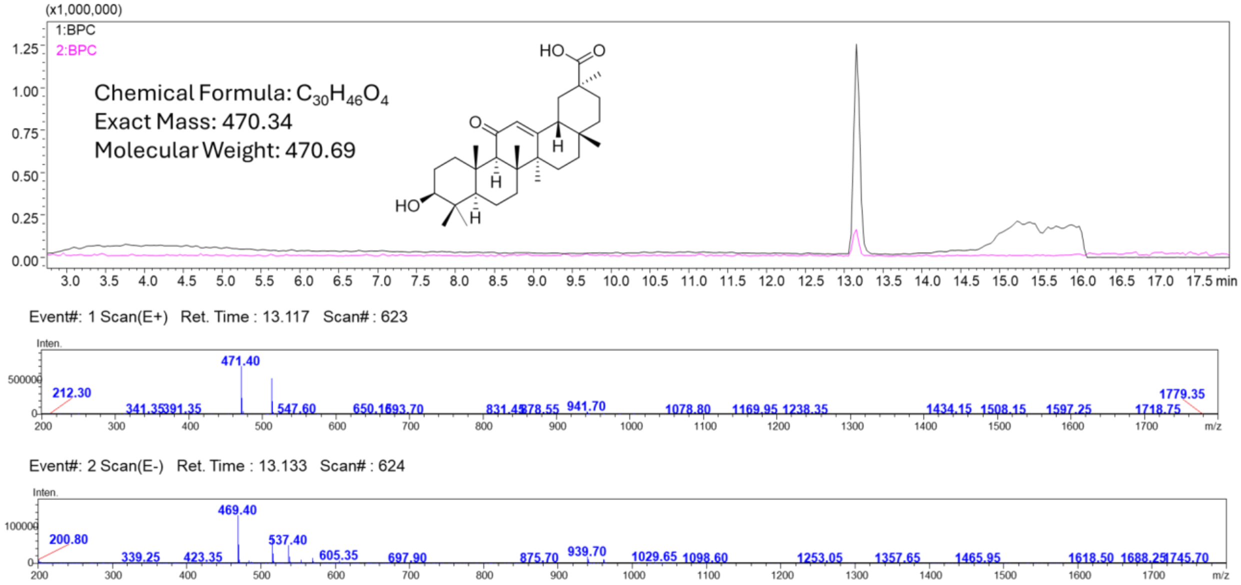

Figure S42. LC-MS chromatogram and MS spectra of glycyrrhetinic acid. Base peak intensity chromatogram and low–low-resolution MS data in positive and negative mode of the peak at t_R= 13.1 min. Column: YMC Pack ODS-AQ (C18) 120 Å, 3 μm (150 x 2.0 mm). Elution gradient: 5:95 ACN (0.1% FA)-H_2O (0.1% FA) to 100:0 ACN (0.1% FA) in 13.5 min, flow 0.3 mL/min.LCMS chromatogram for glycyrrhetinic acid.

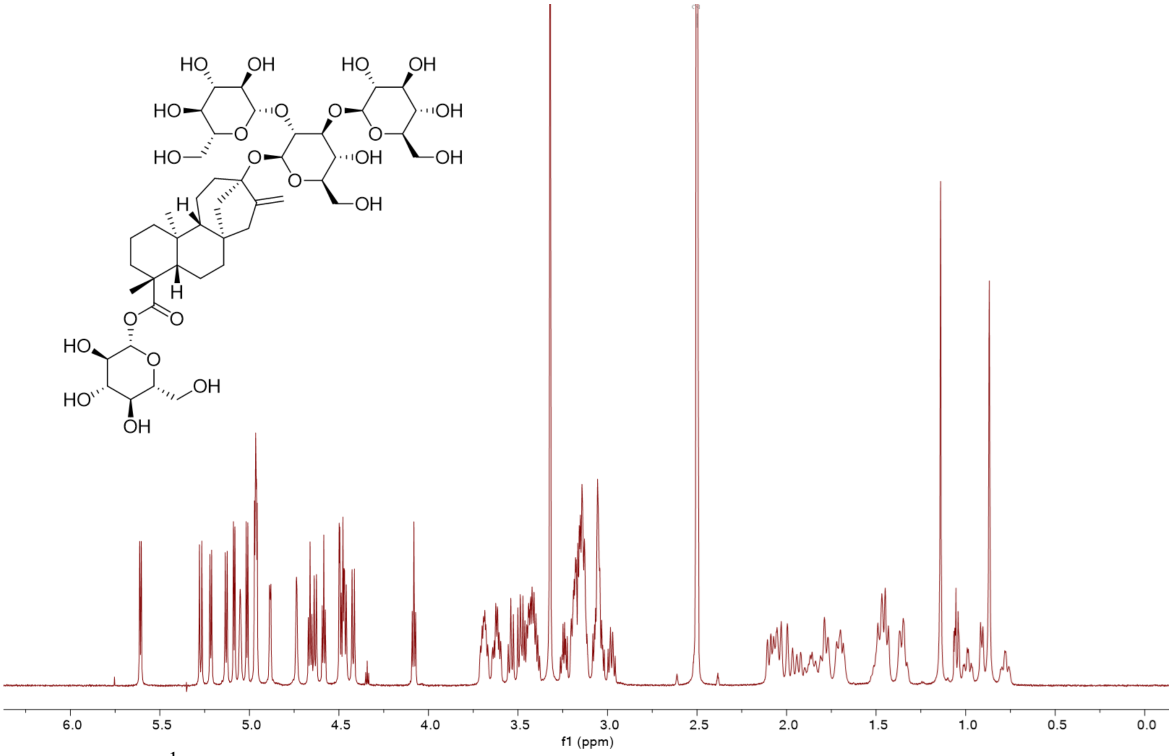

Figure S43. ^{1}H NMR spectrum of rebaudioside A at 600 MHz in DMSO-d_6.

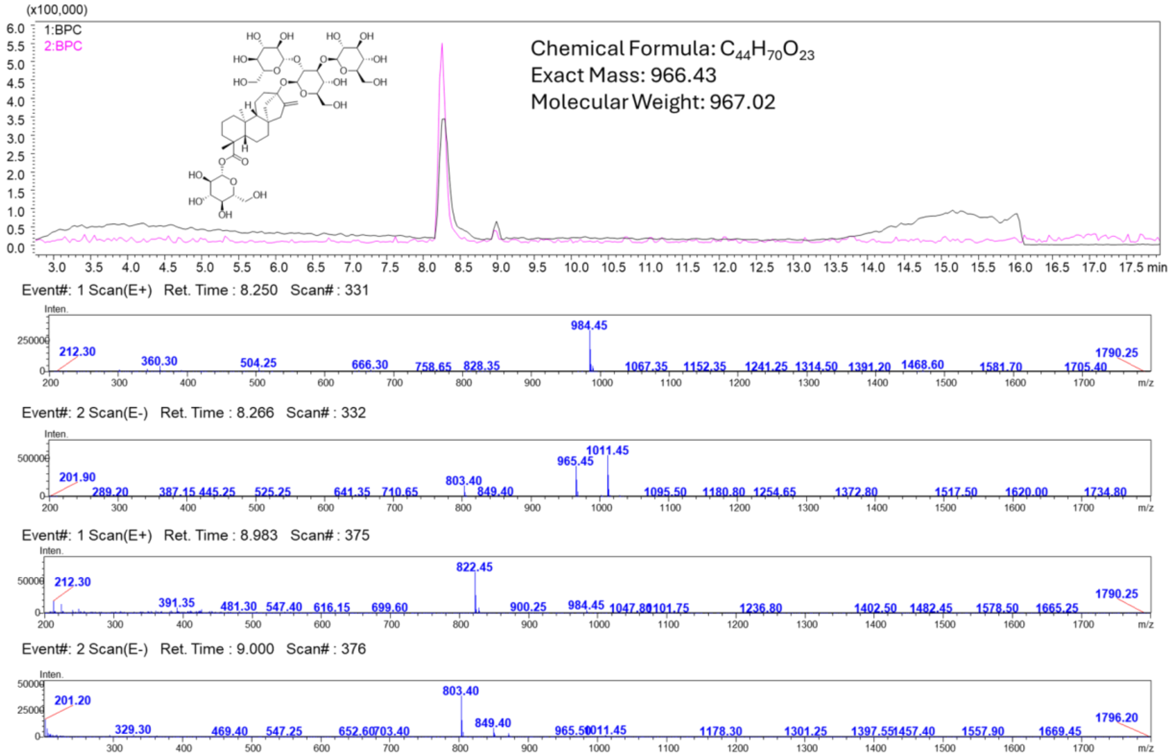

Figure S44. LC-MS chromatogram and MS spectra of rebaudioside A. Base peak intensity chromatogram and low–low-resolution MS data in positive and negative mode of the peaks at t_R= 8.3 and 8.9 min. Column: YMC Pack ODS-AQ (C18) 120 Å, 3 µm (150 x 2.0 mm). Elution gradient: 5:95 ACN (0.1% FA)-H_2O (0.1% FA) to 100:0 ACN (0.1% FA) in 13.5 min, flow 0.3 mL/min.LCMS chromatogram for glycyrrhetinic acid.

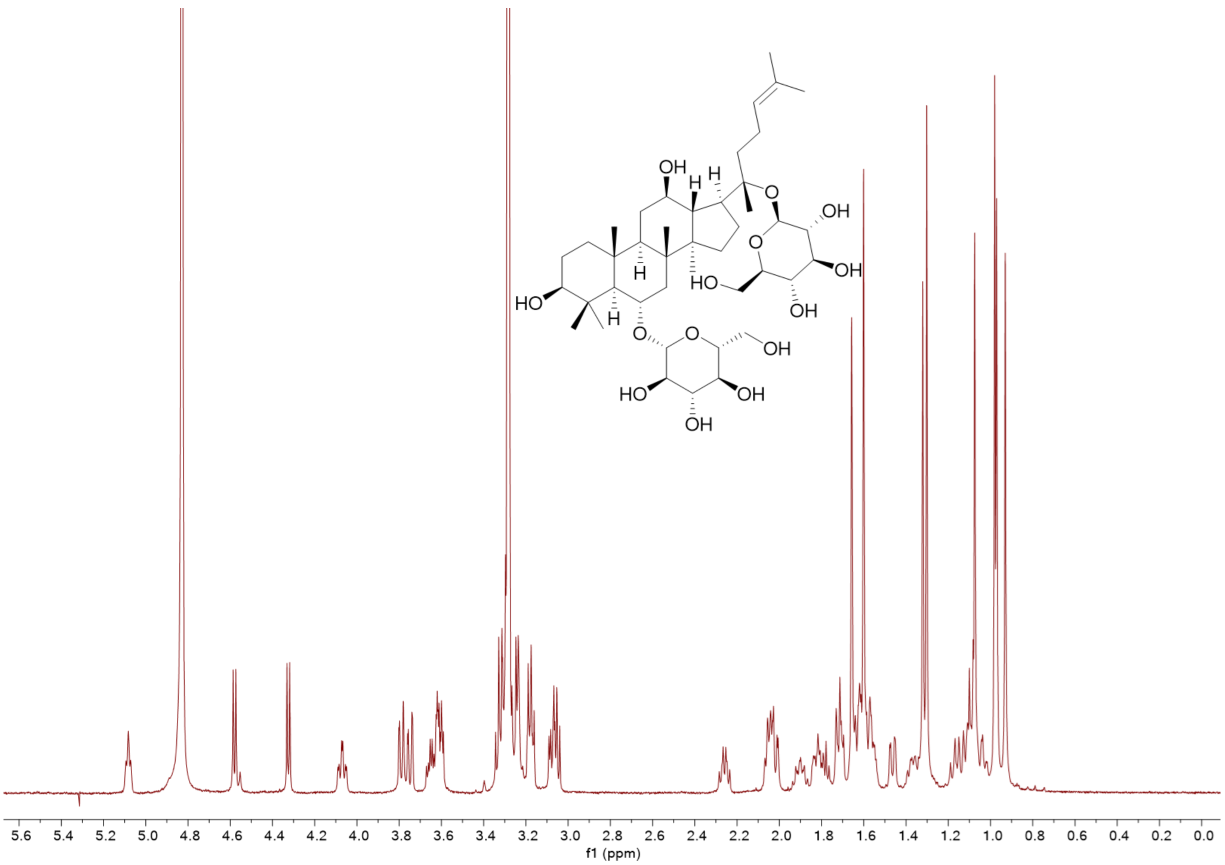

Figure S45. ^{1}H NMR spectrum of ginsenoside Rg1 at 600 MHz in MeOD.

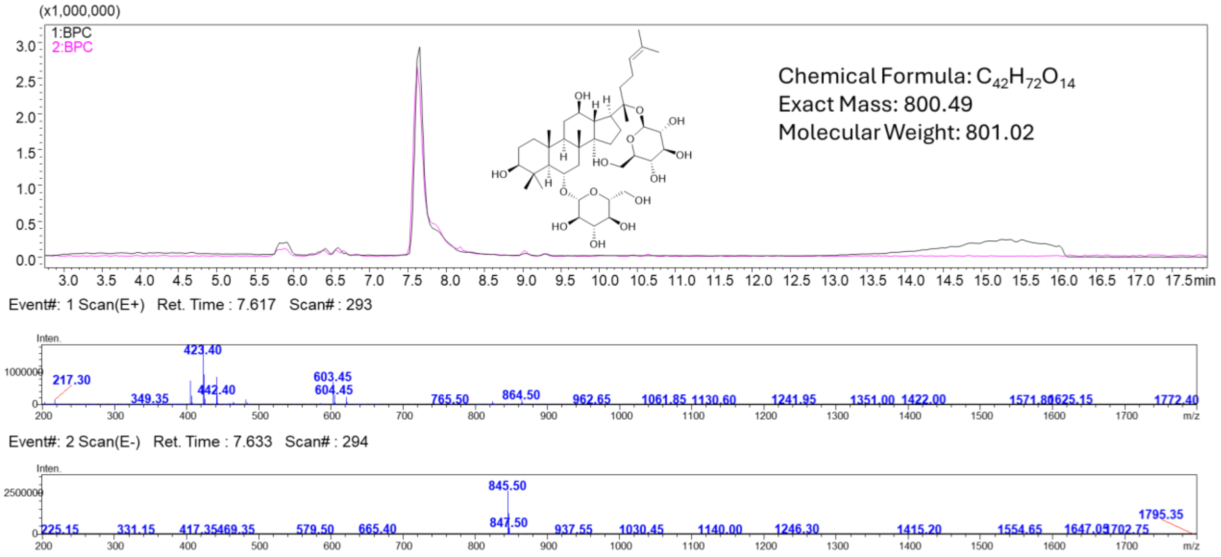

Figure S46. LC-MS chromatogram and MS spectra of ginsenoside Rg1. Base peak intensity chromatogram and low–low-resolution MS data in positive and negative mode of the peak at t_R= 7.6 min. Column: YMC Pack ODS-AQ (C18) 120 Å, 3 µm (150 x 2.0 mm). Elution gradient: 5:95 ACN (0.1% FA)-H_2O (0.1% FA) to 100:0 ACN (0.1% FA) in 13.5 min, flow 0.3 mL/min.LCMS chromatogram for glycyrrhetinic acid.

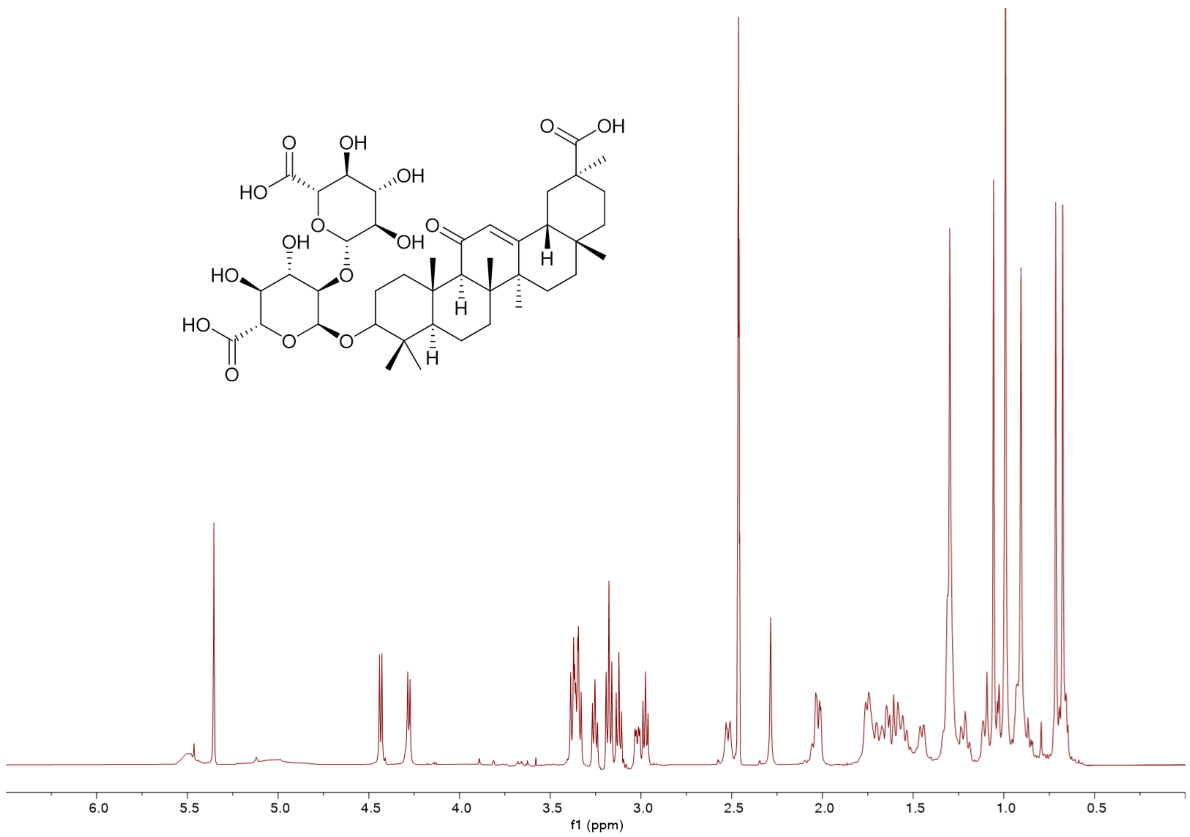

Figure S47. ^1H NMR spectrum of glycyrrhizin at 600 MHz in DMSO-d_6.

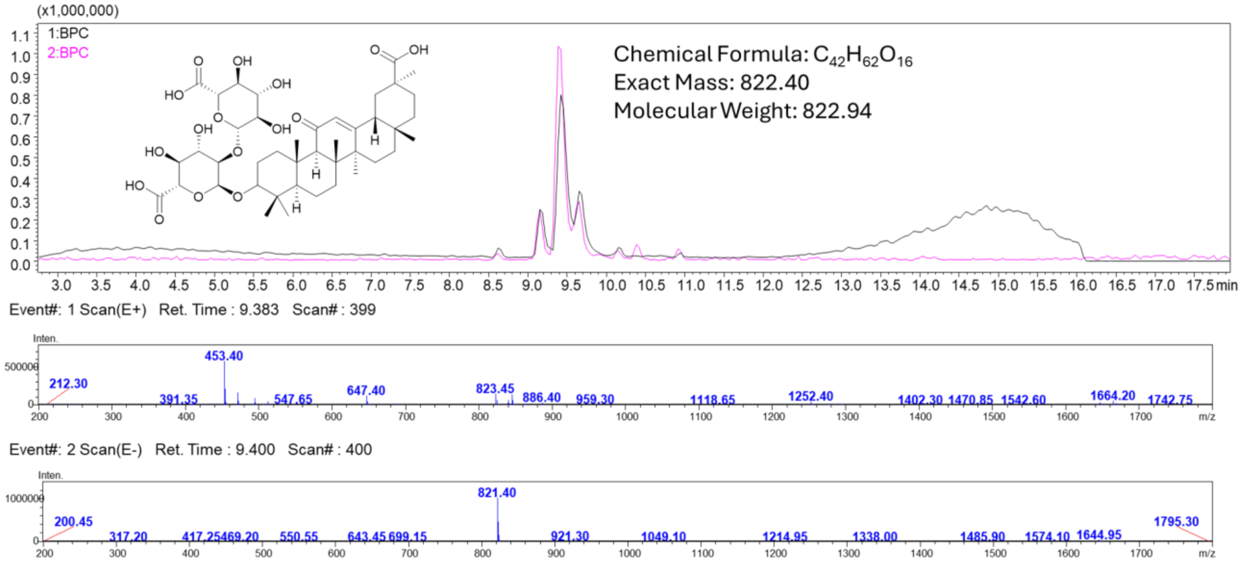

Figure S48. LC-MS chromatogram and MS spectra of glycyrrhizin. Base peak intensity chromatogram and low–low-resolution MS data in positive and negative mode of the peak at t_R= 9.4 min. Column: YMC Pack ODS-AQ (C18) 120 Å, 3 µm (150 x 2.0 mm). Elution gradient: 5:95 ACN (0.1% FA)-H_2O (0.1% FA) to 100:0 ACN (0.1% FA) in 13.5 min, flow 0.3 mL/min.

Table S1. UHPLC-based purities of the isolates of **1** and **2** in the subfractions of T22.

	**Percentage of components (%)**		
**Compounds**	T22-II	T22-III	T22-IV
Peak A	1.8	-	-
**1**	84.7	5.9	-
**2**	13.5	94.1	32.0
Peak D	-	-	68.0

Table S2. Yields of the antiviral active fractions, T18 to T31.

**Tube**	**Weight in each replicate for bioassay (mg)**	**Total weight of the sample (mg)**	**Total weight by compounds (mg)**
18		0.7556	
19		0.4889	
20	0.176	1.0111	Compound **1** 5.765 (0.19% of IQC90)
21		2.5111	
22	0.338	4.4857	
23		2.2778	Compound **2** 5.6762 (0.19% of IQC90)
24	0.095	1.1556	
25		0.7444	
26		0.6143	
27		0.2000	
28		0.2000	
29		0.0429	
30		0.2143	
**Total**	**0.609**	**14.7016 (0.49% of IQC90)**	
